# Unraveling Solvent-Independent Excited State Proton Transfer Dynamics in Sterically Substituted Photoactive Systems

**DOI:** 10.1021/acs.jpclett.6c01594

**Published:** 2026-06-13

**Authors:** Zofia Majewska, Jacob Eller, Kexin Pan, Jack Dalton, Bernd Herzog, Nicholas D. M. Hine, Vasilios G. Stavros

**Affiliations:** † School of Chemistry, 1724 University of Birmingham, Edgbaston, Birmingham B15 2TT, United Kingdom; ‡ Department of Physics, 2707 University of Warwick, Coventry CV4 7AL, United Kingdom; § Department of Pharmaceutical Sciences, 27209 University of Basel, 4056 Basel, Switzerland

## Abstract

Excited state proton transfer (ESPT) plays a central role in the function of many photoactive molecules; however, it can be strongly influenced by environment polarity. Here, we investigate the excited state photodynamics of two structurally complex triazine- and benzotriazole-based molecules that bear sterically bulky substituents around the proton transfer unit: bis-ethyl­hexyl­oxy­phenol methoxyphenyl triazine (BEMT) and methylene bis-benzo­tri­azolyl tetra­methyl­butyl­phenol (MBBT). Using femtosecond transient electronic absorption spectroscopy in solvents of contrasting polarity and hydrogen-bonding character, supported by mixed-reference spin-flip time-dependent density functional theory calculations, we show that ultrafast relaxation in both systems is governed by a largely solvent-insensitive intramolecular ESPT. We demonstrate that the solvent-independent behavior primarily arises from intrinsic, electronic properties of BEMT and MBBT rather than steric substitution. While bulky substituents are not required for efficient intramolecular ESPT, they offer enhanced solvent-invariant ESPT dynamics. This highlights potential design elements for next-generation photoactive systems for tuning excited state behavior.

## Graphical abstract

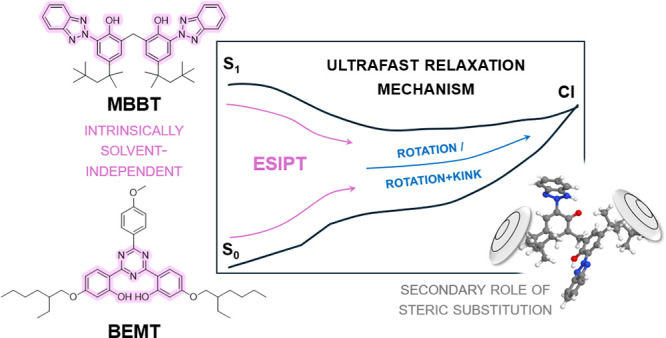

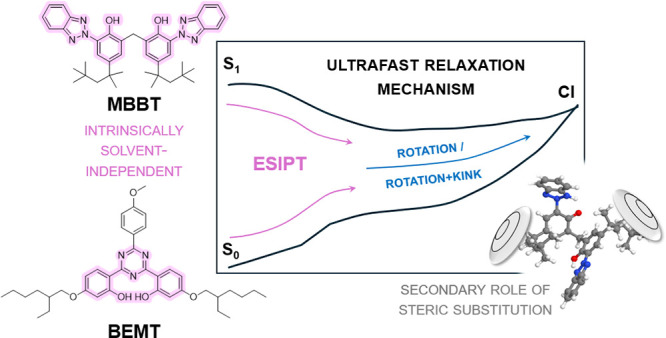

Excited state proton transfer (ESPT) is a ubiquitous photochemical process underpinning nonradiative excited state relaxation and related molecular functions across a wide range of systems,^1–4^ including biological chromophores,^5–8^ fluorescent sensors and bioimaging probes,^9–13^ photostabilizers,^14–16^ and optoelectronic materials such as organic light-emitting diodes.^17–20^ ESPT can proceed either intramolecularly (excited state intramolecular proton transfer, ESIPT) between a proton donor (commonly an −OH group) and a proton acceptor (typically N or S atoms)^21^ or through interactions with the surrounding medium, with solvent polarity and hydrogen-bonding capability frequently invoked as key factors governing its efficiency.^22–28^ In molecules that undergo ESIPT, an intramolecular hydrogen-bonding (IHB) motif defines the proton transfer coordinate in the excited state, enabling rapid and efficient population of the tautomeric configuration (typically less than a 150 fs time scale).^29,30^ However, in polar or protic environments, competitive hydrogen bonding with the solvent can disrupt IHB and suppress the proton transfer-mediated relaxation, often causing undesired modifications to the fluorescence signal.^31–35^ Thus far, substituent choice in ESIPT systems has focused predominantly on tuning the electronic structure of the chromophore to control proton transfer.^30,36–38^ Recent approaches include the addition of electric field generating charged groups,^39^ donor–acceptor modulation of charge transfer,^40^ or the incorporation of “tail” groups controlling protonation and hydrogen-bonding ability.^41^ While effective, such electronic tuning inherently couples ESIPT to the environment, posing challenges where a more consistent response across diverse environments is desired. This motivates the question of whether steric shielding from the external perturbations can provide a more robust, environment-independent performance.^42–44^

Specifically, two photoactive systems, bis-ethyl­hexyl­oxy­phenol methoxyphenyl triazine (BEMT) and methylene bis-benzo­tri­azolyl tetra­methyl­butyl­phenol (MBBT) (chemical structures shown in Figure 1a,b), have been hypothesized to undergo ESIPT.^45–47^ Both are important active ingredients in sunscreens due to their beneficial physicochemical properties, including excellent photostability and broad-spectrum ultraviolet (UV) absorption.^47^ We speculate that ESIPT within these molecules is facilitated by a nonplanar conformation arising from the presence of sterically bulky substituents around their central units. Due to the large size and chemical complexity of BEMT and MBBT, their excited state relaxation mechanisms remain only inferred rather than directly elucidated, with current expectations based solely on studies of simpler core motifs, namely 2-(2′-hydroxy­phenyl)-1,3,5-triazine and 2-(2′-hydroxy­phenyl)­benzo­triazole.^48–55^ Simulations by Paterson et al. demonstrated that ESIPT plays a central role in accessing a conical intersection (CI) in both core motifs.^53,54^ However, it would be an oversimplification to assume that the relaxation mechanisms of BEMT and MBBT are identical to those of their smaller counterparts. These larger molecules have higher degrees of structural symmetry, transition densities that spread across the entire length of the molecules (see Figure 1c–f), and increased potential for intramolecular steric hindrance, all of which could significantly affect the character of occupied electronic states or the relaxation trajectories. Thus, a unified experimental and computational investigation of intact molecules is critical to definitively elucidate their behavior.

**Figure 1:**
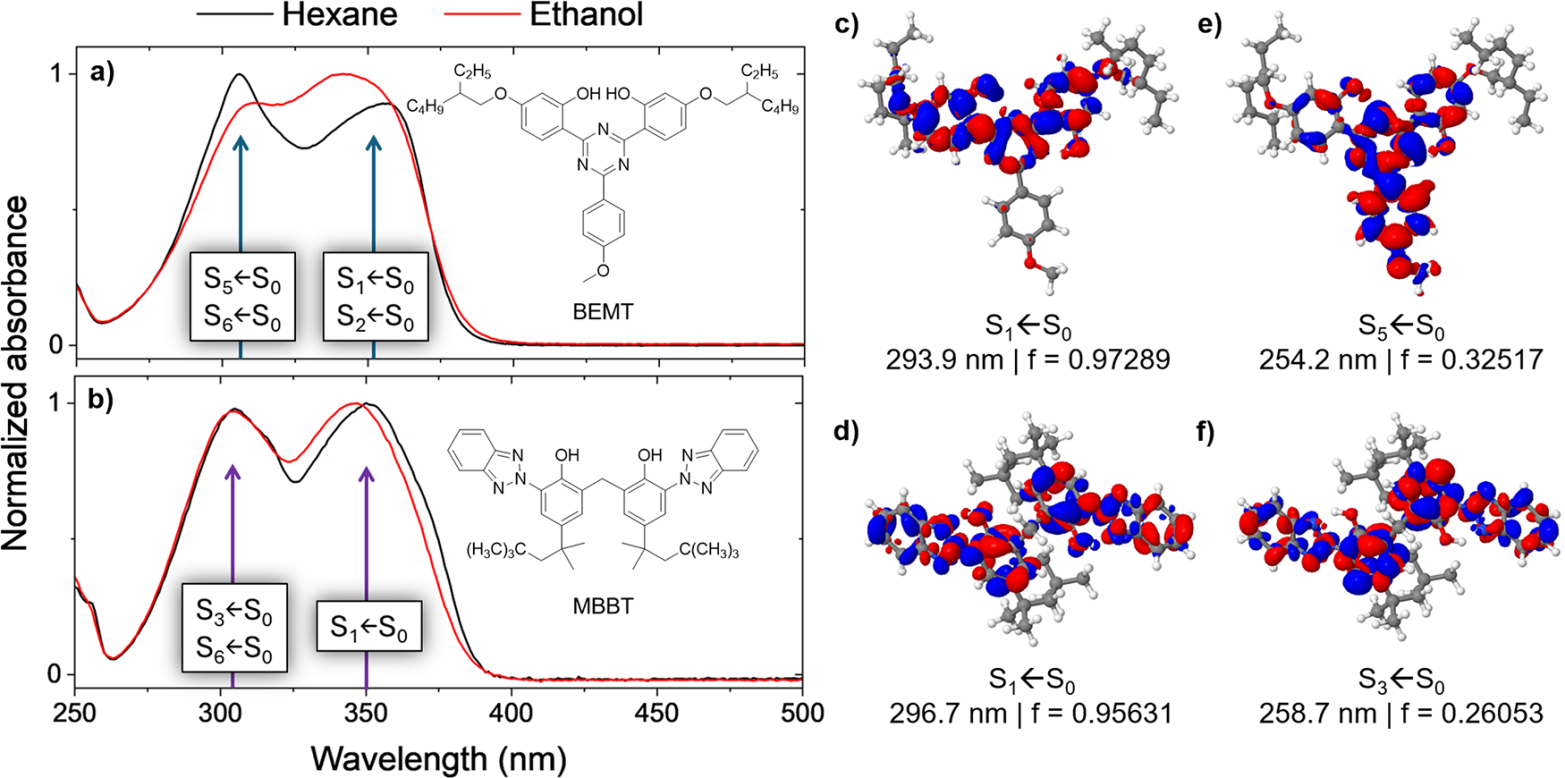
Absorption spectra with labels of all transitions with non-negligible contributions to each spectral peak for (a) BEMT and (b) MBBT in hexane (black line) and ethanol (red line). Molecular structures and transitions contributing to each experimental band are displayed in the graph insets. Transition densities as predicted by ωB97X for the transitions with the strongest calculated contributions to each experimental band for (c) BEMT S_1_ ← S_0_ for the UVA peak, (d) MBBT S_1_ ← S_0_ for the UVA peak, (e) S_5_ ← S_0_ in BEMT for the UVB peak, and (f) S_3_ ← S_0_ in MBBT for the UVB peak.

To assess the plausibility of systems accessing specific CI geometries, theoretical methods are used to trace energy surfaces along paths that connect Franck–Condon (FC) geometries to minimum energy CIs (MECIs). Hence, the chosen level of theory must accurately and equivalently describe ground and excited state potential energy surfaces (PESs) along and around their seams of degeneracy. Historically, the most common approach to accurately capture CIs has been to use multireference wave function methods, such as CASSCF/CASPT2.^56–59^ However, these methods are computationally intense, with their expense scaling significantly with system size and the size of the selected active space of molecular orbitals. Thus, these methods are unsuitable for elucidating the excited state behavior of large molecules like BEMT and MBBT. Despite linear response (LR-) time-dependent density functional theory (TDDFT) offering much more favorable scaling than multireference wave function methods, it has well-documented systematic weaknesses in the calculation of PESs in the vicinity of S_*n*_–S_0_ (*n* > 0) CIs.^60–62^ Spin-flip TDDFT (SF-TDDFT) also offers relatively efficient scaling and improves the treatment of CIs; however, it introduces a new issue where the desired singlet state is significantly contaminated by the character of a triplet state.^63^ In recent years, mixed-reference spin-flip TDDFT (MRSF-TDDFT) has been developed: this advanced technique accurately captures ground and excited state PESs in the vicinity of CIs with minimal spin contamination.^63–66^ Although it is computationally more demanding than LR-TDDFT and SF-TDDFT, MRSF-TDDFT scales to significantly larger systems than multireference wave function methods while retaining competitive accuracy. Despite the usefulness of MRSF-TDDFT in elucidating nonradiative relaxation mechanisms in large molecular systems, it remains relatively underutilized compared to other methodologies. For further discussion of available methods, see Supporting Information (SI), section 2.1.

In this study, we explore the influence of solvent environment on a pair of structurally complex ESIPT-active systems, BEMT and MBBT, that extend far beyond the regime of previously studied core motifs. The aim of this work is to elucidate (i) what role proton transfer has in the excited state relaxation pathway, (ii) whether this process is intrinsic to the molecular framework or mediated by solvent interactions, and (iii) what impact the sterically bulky substitution has on maintaining this mechanism across solvents of contrasting polarity and hydrogen-bonding character. By combining femtosecond transient electronic absorption spectroscopy (fs-TEAS) and calculations at the MRSF-TDDFT level of theory, we show that ESIPT governs the ultrafast relaxation of BEMT and MBBT following UV excitation. Notably, experimental data show that the associated dynamics are preserved across solvents of contrasting polarity, which indicates that ESIPT operates as a robust and largely self-contained relaxation pathway. Theoretical results fully elucidate these excited state relaxation mechanisms, demonstrating their energetic feasibility by tracing corresponding PESs. Comparison of MBBT with its simpler analogue shows that the solvent-independent behavior is primarily governed by the intrinsic properties of the benzotriazole scaffold, which preserve efficient deactivation irrespective of substitution. Bulky substituents play a secondary role, helping to maintain a geometry favorable for ESIPT and mitigating disruption of the proton transfer coordinate in polar environments. Collectively, these results establish benzotriazole (and by extension triazine) motifs as powerful building blocks with intrinsically solvent-resilient ESIPT, whose performance can be further stabilized through judicious functionalization. Understanding the ESIPT-mediated relaxation investigated here may be transferable to other complex chromophores, offering guidance for the design and optimization of functional materials in photoprotection and related photochemical applications.

## Steady-State Absorption and Emission

To test whether proton transfer in BEMT and MBBT is independent of solvent environment, we examined their photochemical properties in a protic polar solvent (ethanol) and a nonpolar aprotic solvent (hexane) to provide media of differing hydrogen-bonding capability. In both solvents, the absorption spectrum for each molecule exhibits two distinct bands spanning the UVA (320–400 nm) and UVB (280–320 nm) regions^67^ (Figure 1a,b). For MBBT, the UVB absorption maximum remains essentially unchanged between hexane and ethanol, whereas BEMT exhibits a modest 6 nm red shift in ethanol. This behavior is consistent with those bands arising from local ππ* transitions, which involve minimal changes in transition dipole moment and are therefore relatively insensitive to solvent polarity; computational results confirm that these are ππ* transitions, as discussed below. In contrast, both molecules show a blue shift in their UVA bands upon moving from hexane to ethanol, with the effect being slight for MBBT but more pronounced for BEMT. This suggests more solvent sensitivity for the molecule with a triazine-based core (BEMT) than that with a benzotriazole core (MBBT), contrary to what was found for smaller versions of those molecules.^51^ Previous experimental research by Sohn et al. reported similar trends for BEMT, explained by the IHB disruption caused by polar solvents.^68^ Both systems display remarkable photostability following irradiation (Figure S2, SI), consistent with efficient ESIPT mechanisms.^47^ Emission measurements reveal negligible radiative decay following excitation of the UVA and UVB peaks (Figure S3, SI), indicating that ultrafast nonradiative processes dominate the relaxation of both molecules.

Gas phase LR-TDDFT calculations for BEMT assign the S_1_ ← S_0_ and S_2_ ← S_0_ transitions to the UVA peak, and the S_5_ ← S_0_ and S_6/7_ ← S_0_ (ωB97X/CAM-B3LYP) transitions to the UVB peak (see Tables S8 and S9, SI). For gas phase MBBT, the UVA peak corresponds almost purely to the S_1_ ← S_0_ transition, and the UVB peak results from the S_3_ ← S_0_ and S_6_ ← S_0_ transitions (see Tables S11, S12, S14, and S15, SI). For both systems, all other S_*n*_ ← S_0_ transitions (up to *n* = 10) were found to be optically dark. The computed transition densities associated with all these transitions display significant ππ* character. Performing calculations for both systems in implicit solvent representing hexane and ethanol had negligible impact on the calculated absorption wavelengths, oscillator strengths, and transition densities associated with the S_1_ ← S_0_ transitions (see section 2.3.1, Figures S21–S26, and Tables S2–S7 of the SI). All calculated vertical excitations and corresponding transition densities as predicted by ωB97X, CAM-B3LYP, and B3LYP functionals are available in Tables S8–S16, as well as Figures 1c–f and S27–S35, SI. Complementary discussion of the results and reasoning behind the use of the CAM-B3LYP and ωB97X functionals is also provided (section 2.3.2.2, SI).

## Femtosecond Transient Electronic Absorption

fs-TEAS reveals that both BEMT and MBBT undergo ultrafast, largely wavelength-independent relaxation following UV excitation, indicating rapid internal conversion from higher-lying excited states to the lowest excited state (S_1_) within the minimum experimental time resolution (∼100 fs). Consequently, the data presented and discussed here follow excitation at the corresponding absorption maxima in the UVA region, with UVB excitation presented in Figure S5 and Table S1 of the SI. The molecules were studied in both ethanol and hexane (chosen as two solvent extremes, under the assumption that any solvent-induced differences in ESIPT dynamics would be most pronounced between such distinct environments), yielding nearly identical results. Accordingly, only the data obtained in hexane are presented here (Figure 2), while the ethanol data are provided in Figure S6 of the SI. For a further comparison, a polar aprotic solvent (acetonitrile) was used to decouple the effects of solvent polarity and proticity on the dynamics, producing near-identical results to those in ethanol and hexane (see Figure S7, SI).

**Figure 2:**
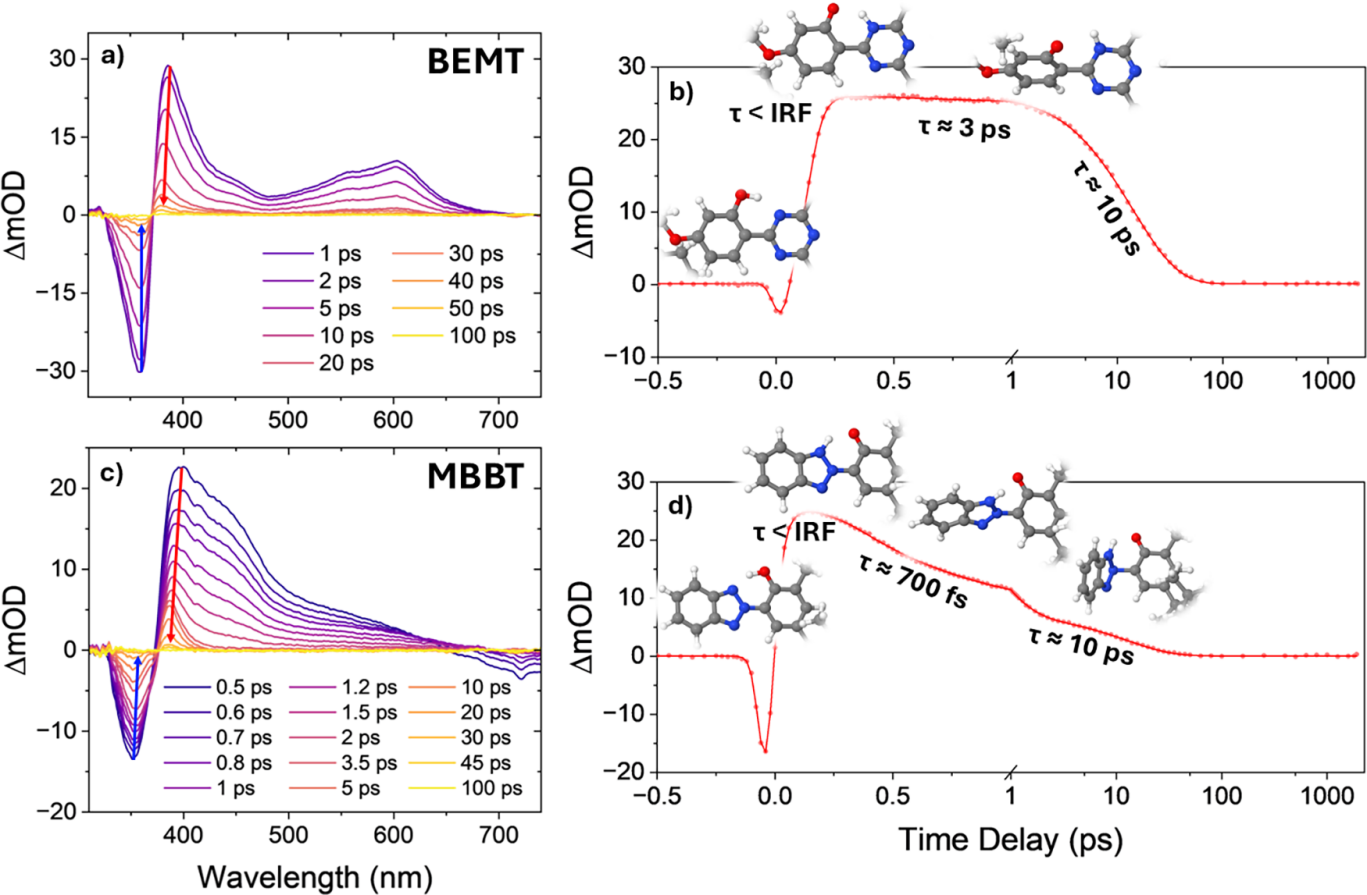
Transient absorption spectra for selected time delays for (a) BEMT in hexane and (c) MBBT in hexane photoexcited at their corresponding UVA maxima. Decay traces for a single wavelength, (b) 380 nm in BEMT and (d) 390 nm in MBBT, with snapshots of the calculated structures along the scanned relaxation trajectory and decay lifetimes extracted from the global sequential fitting. The time delay is linear up to 1 ps and logarithmic thereafter.

Both BEMT and MBBT exhibit similar transient absorption features, including a negative feature around 335–370 nm (BEMT) and 340–365 nm (MBBT) assigned to ground state bleach, and a broad positive feature near 390–650 nm assigned to excited state absorption. A short-lived (∼500 fs) negative feature centered at ∼700 nm is observed for MBBT in both hexane and ethanol, which is assigned to stimulated emission (SE), consistent with population of a keto tautomer following ESIPT.^50^ The significant red shift of SE from the ground state bleach at early times implies substantial ultrafast excited state structural reorganization, in line with the pronounced geometric relaxation accompanying formation of the proton-transferred configuration. This behavior suggests that the IHB is retained in both solvents, contrary to what is typically reported for most ESIPT-active molecules.^25,35,69,70^ By contrast, no comparably red shifted SE is detected for BEMT. Notably, this absence persists in both hexane and ethanol, ruling out solvent-induced disruption of the IHB as the dominant factor. Calculations (discussed in the next section) suggest a more red shifted keto state emission for BEMT than MBBT, although no SE was observed when the probe region was extended to 650–950 nm (see Figure S8, SI). Therefore, we conclude that SE from the keto state in BEMT is obscured by overlapping excited state absorption.

MBBT undergoes efficient relaxation, exhibiting complete recovery of the ground state bleach and no formation of long-lived photoproducts. In contrast, BEMT yields a very minor photoproduct (<1 ΔmOD), confirmed through a comparison of absorption spectra pre- and post-single wavelength irradiation measurement (see Figure S9, SI). Its formation depends strongly on both solvent and excitation energy. In ethanol, photoproduct formation is observed at both excitation wavelengths (UVA and UVB), whereas in hexane it occurs only under higher-energy excitation (UVB), consistent with population of higher-lying excited states. The increased yield at shorter wavelengths therefore suggests that the photoproduct most likely arises from a minor relaxation pathway accessed from higher energy states, rather than from dynamics on the primary S_1_ surface.

To attain a quantitative insight into the transient absorption features, the data is fitted using a global sequential fitting model.^71^ The lifetimes obtained from the analysis, corresponding to photoexcitation to the lowest-lying electronically excited state (in the UVA region), are presented in Table 1 and summarized in Figure 2b,d. For BEMT, there are two lifetimes present in both solvents, the first lifetime, τ_1_, being on a time scale of ∼3 ps and the second lifetime, τ_2_, on a time scale of ∼10 ps. There is an additional third lifetime for this molecule in ethanol to capture the long-lived species lasting beyond the experimental time window (1.8 ns). For MBBT, there are three lifetimes in both solvents. The first lifetime, τ_1_, encapsulates the fast-decaying SE present at the early time delays. The remaining two lifetimes, τ_2_ and τ_3_, are ∼700 fs and ∼10 ps, suggesting the presence of two additional transient species in the overall deactivation mechanism. Evolution associated difference spectra (EADS) representing the evolving spectral features corresponding to each extracted lifetime are presented in Figures S5 and S6, SI.

**Table 1:** Results of the Global Sequential Fitting for the Data Obtained for BEMT and MBBT Photoexcited to the First Excited State (S_1_), Where λ_ext_ Refers to the Wavelength of Excitation Used in the fs-TEAS Measurement

BEMT		
Solvent/λ_ext_	Hexane/356 nm	Ethanol/343 nm
τ_1_/ps	3.38 ± 0.06	2.57 ± 0.06
τ_2_/ps	13.1 ± 0.08	6.56 ± 0.07
τ_3_/ps	-	>1800

MBBT		
Solvent/λ_ext_	Hexane/350 nm	Ethanol/345 nm
τ_1_/ps	0.23 ± 0.06	0.12 ± 0.06
τ_2_/ps	0.74 ± 0.06	0.68 ± 0.06
τ_3_/ps	10.8 ± 0.20	10.7 ± 0.30

Upon photoexcitation of BEMT, τ_1_ represents the initial evolution from the FC geometry, including ultrafast ESIPT that occurs within the experimental time resolution and is therefore not resolved as a separate step (see next section for further discussion). τ_2_ corresponds to an internal conversion to the S_0_ electronic ground state of the keto form, followed by ground state intramolecular proton transfer (GSIPT) to re-establish the enol form and vibrational cooling from the higher- to lower-lying vibrational levels within the S_0_ state. All excited state population decays on a few picoseconds time scale, indicating highly efficient relaxation of BEMT. For MBBT, τ_1_ reflects both relaxation from the FC geometry via ESIPT, leading to the keto tautomer (and associated SE), and further evolution resulting in decay of the SE. τ_2_ is attributed to internal conversion to S_0_ within the keto geometry. Finally, τ_3_ encompasses GSIPT back to the enol form and vibrational cooling within the S_0_ state. MBBT reaches full recovery of the ground state population, indicating exceptionally high efficiency.

Historically, simple ESIPT-active chromophores were found to be highly sensitive to solvent environment, with polar or polar protic media often suppressing proton transfer through disruption of the IHB between proton donor and acceptor.^35,44,48,49,72–76^ Contrary to this, we report that both complex systems investigated here dissipate excitation energy with comparable efficiency in polar protic and nonpolar aprotic environments, indicating that proton transfer proceeds through a predominantly unperturbed intramolecular pathway.

To decouple steric effects potentially causing this anomaly, from structure-intrinsic behavior, we compared MBBT to its simpler analogue, 2-(2′-hydroxy-5′-methyl­phenyl)­benzo­tri­azole (see Figure S10, SI, for molecular structure and absorption spectra), measured in both hexane and ethanol. It was selected as the reference system, as it is a widely used polymer photoprotectant that is readily available and has been extensively characterized in the literature. It displays spectral features closely resembling those of MBBT, including the characteristic appearance of the SE from the keto state, indicating that ESIPT remains within our instrument response (see Figure S11, SI). The overall dynamics are largely unchanged between the solvents, with only subtle differences such as the elongation of the spectral features assigned to vibrational cooling and GSIPT in ethanol versus hexane. While this indicates that the simpler molecule experiences some perturbations from the polar environment in the final steps of the relaxation, the early dynamics appear to remain unaffected.

Radial distribution functions of ethanolic hydroxyl hydrogens around the acceptor nitrogen atoms in MBBT and its smaller counterpart, obtained from 1000 ps explicit solvent molecular dynamics simulations, provide an explanation for these slight differences (see Figures S36 and S37, SI). Ethanol is more readily able to closely interact with the nitrogen atom of the smaller analogue than with that in MBBT. Additionally, throughout the 1000 ps simulations, the hydroxyl proton within the smaller analogue showed a much greater tendency for rotating to face away from the acceptor nitrogen than the equivalent proton in MBBT (see Figures S38 and S39, SI). This suggests that the proton in MBBT spends more time in the optimal orientation for ESIPT than the proton in the smaller analogue, which appears to be more readily displaced by polar protic ethanol.

Despite these results, we observe efficient ESIPT-driven dynamics in both simple and complex benzotriazole systems, indicating that, while rich substitution helps maintain the proton transfer coordinate, its role is only secondary and is not essential to achieve solvent-invariant ESIPT. Instead, solvent independence is an intrinsic property of benzotriazole systems. Theory indicates that the proton transfer step in both MBBT (see next section) and its smaller analogue^55^ is effectively barrierless; therefore, solvent polarity is unlikely to introduce a significant activation barrier to the excited state landscape that would alter the dominant relaxation pathway. Additionally, the predominantly local ππ* character of the photoexcitation in MBBT (see Figure 1d,f for transition densities) implies little dipole reorganization upon excitation, which minimizes the polar solvent stabilization of competing tautomeric excited states. We observe a similar favorable excited state landscape and equivalent electronic properties in BEMT, suggesting that the above conclusions can potentially also be extended to the triazine motif.

Together, these findings showcase the intrinsic ultrafast dynamics of benzotriazole-based (and by extension triazine-based) ESIPT motifs across various environments, which can be further improved through bulky functionalization to maintain optimal geometry for proton transfer, while also providing desired practical properties, such as improved oil solubility and higher molar mass, which are both desirable features of sunscreen UV filters.^77^

## Elucidation of Excited State Behavior

As previously noted, LR-TDDFT predicts very similar S_1_ ← S_0_ absorption energies, oscillator strengths, and transition densities for BEMT and MBBT in ethanol, hexane, and vacuum (see section 2.3.1, Figures S21–S26, and Tables S2–S7 of the SI). This indicates that the corresponding electronic transitions from ground to first excited states are very similar. Furthermore, scans along ESIPT reaction coordinates in implicit ethanol and hexane demonstrate that the gradients of the PES along this coordinate are largely solvent-independent (Figure S48, SI). Also, as discussed in the previous section, fs-TEAS experimental data (see sections 1.4.2.1 and 1.4.2.2, SI) show that the excited state behaviors of solvated BEMT and MBBT are very similar in hexane and ethanol (and acetonitrile), suggesting that it is unnecessary to account for the influence of solvent molecules either explicitly or implicitly in theory calculations. Additionally, OpenQP/1.0 (our chosen MRSF-TDDFT software) has not yet implemented implicit solvation, and the inclusion of explicit solvent molecules would significantly increase associated computational cost. Thus, the results of all TDDFT calculations discussed hereafter correspond to BEMT and MBBT simulated in vacuum.

To achieve the observed fast relaxation, BEMT and MBBT are expected to undergo nonadiabatic transitions through S_1_/S_0_ CIs; our computational results support this hypothesis with energetically accessible MECIs for both molecules. Since the identified relaxation mechanisms for BEMT and MBBT were very similar in calculations using ωB97X and CAM-B3LYP, energetic properties reported below solely correspond to MRSF-TDDFT evaluations using ωB97X. Energy surfaces calculated with CAM-B3LYP are displayed in Figures S43–S47, SI, and geometric properties (θ_1_, θ_2_, φ_S_, and φ_M_ in Figures 3 and 4) of the FC and CI geometries are available in Tables S17 and S18, SI. Two starting geometries of MBBT were used throughout this work; however, due to difficulties obtaining converged calculations for both conformers, results for only one of the conformers are presented in the main body (referred to as “MBBT 21”; see section 2.2, SI, for more details regarding the generation of each MBBT geometry). The calculations revealed that BEMT and MBBT have their own distinct relaxation mechanisms, summarized in Figures 3 and 4, respectively, which explain the experimentally observed slight differences in spectral features and their evolution time scales between the two systems.

**Figure 3:**
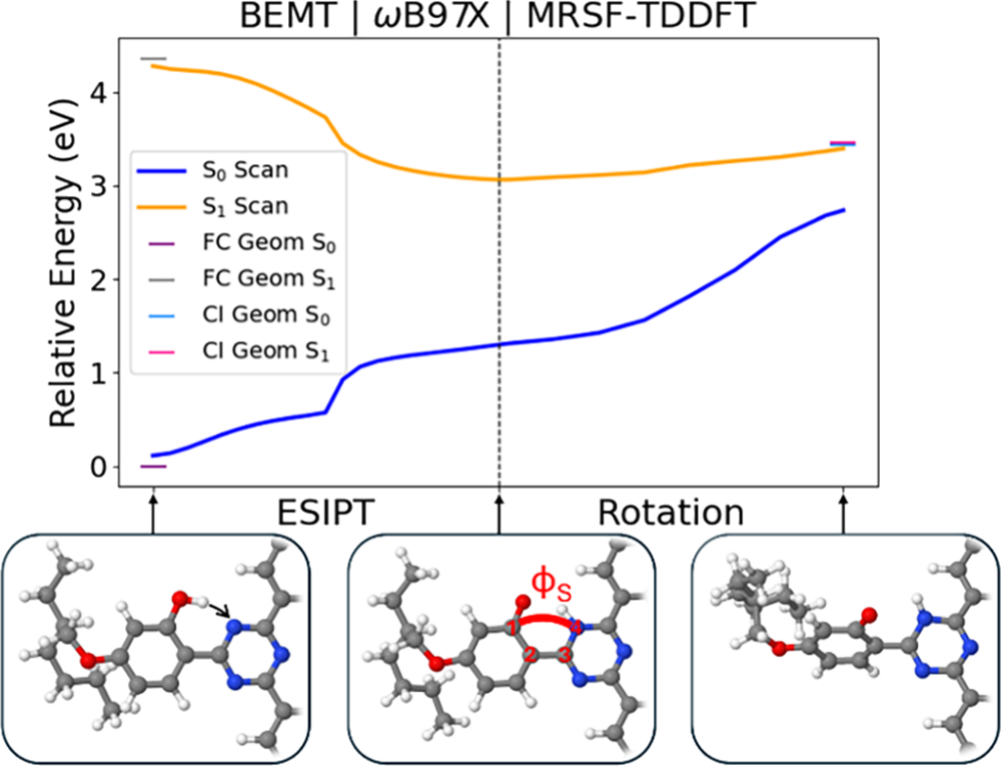
Energy surfaces associated with the approximated relaxation trajectories of BEMT. φ_S_ refers to the dihedral angle that was iteratively adjusted to approximate the relaxation trajectories. All energies were calculated using MRSF-TDDFT with the ωB97X functional. Plots of identical surfaces with explicitly marked data points can be seen in Figure S43b, SI.

**Figure 4:**
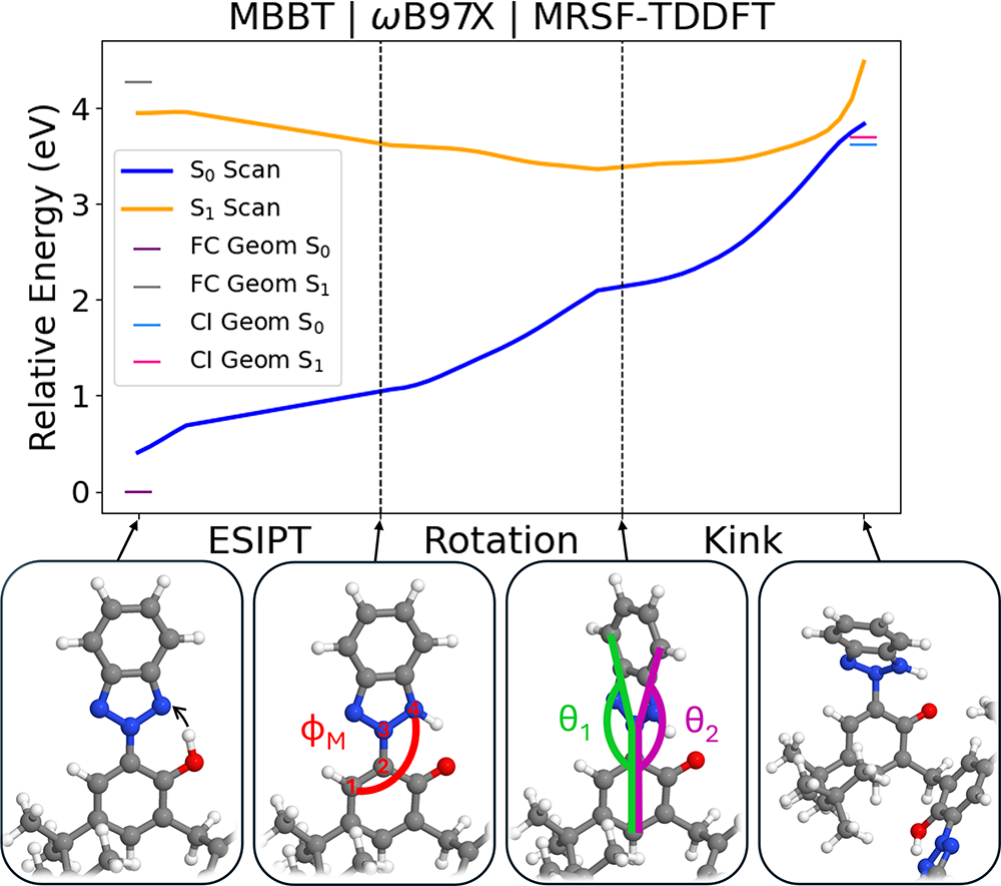
Energy surfaces associated with the approximated relaxation trajectories of MBBT. θ_1_, θ_2_, and φ_M_ refer to bonds (θ) and dihedral angles (φ) that were iteratively adjusted to approximate the relaxation trajectories. All energies were calculated using MRSF-TDDFT with the ωB97X functional. Plots of identical surfaces with explicitly marked data points can be seen in Figure S46b, SI.

Upon excitation to S_1_, both BEMT and MBBT undergo ultrafast proton transfer of a phenolic proton to an acceptor nitrogen: in BEMT, this nitrogen is situated on the triazine ring between two large substituents; in MBBT, it is located on the benzotriazole moiety. ESIPT lowers the S_1_ energies of BEMT and MBBT by 1.30 and 0.66 eV, respectively, while simultaneously raising S_0_ energies and reducing S_1_–S_0_ gaps by 2.60 eV (BEMT) and 1.73 eV (MBBT) (Tables S19 and S20, SI). This large reduction in energy gap explains the pronounced, red shifted emission of the MBBT keto tautomer and predicts an even more pronounced red shift for BEMT, into the near-infrared region. For BEMT, the proton transfer is unambiguously energetically downhill from the FC point, whereas the scanned trajectory for MBBT displays a small energy barrier in the initial stages of ESIPT before becoming energetically downhill. Deuteration experiments ruled out quantum tunneling, as no kinetic isotope effect was observed and tautomerization remained complete within the instrument response (<100 fs, Figure S12, SI). This confirms barrierless, ultrafast proton transfer for both molecules, meaning the barrier observed in Figure 4 originates from a slight mismatch in geometries between the physical and scanned trajectories.

Following ESIPT, BEMT moves toward the S_1_/S_0_ CI via a dihedral rotation (φ_S_, Figure 3), which increases S_1_ by 0.404 eV and reduces the S_1_–S_0_ gap by 0.981 eV. Minor atomic rearrangements then lead to the CI, 0.898 eV below the FC geometry, with a 0.019 eV S_1_–S_0_ gap. MBBT undergoes dihedral rotation (φ_M_) and benzotriazole kinking (θ_1_/θ_2_, Figure 4). Applying the kinking distortion in isolation yields a structure only 0.028 eV below the FC geometry. Though the rotation alone stabilizes S_1_ by 0.248 eV and narrows the gap by 1.280 eV, subsequent kinking initially raises S_1_ but rapidly contracts the S_1_–S_0_ gap. The final CI lies 0.575 eV below the FC geometry with an S_1_–S_0_ gap of 0.077 eV. Constrained suboptimal geometries cause apparent S_1_ spikes, but smooth CI approaches are expected to be consistent with our CAM-B3LYP MRSF-TDDFT results (Figure S44d, SI). Post-internal conversion, LR-TDDFT confirms facile GSIPT for both molecules, consistent with low photodegradation.

Although calculations treat motions independently, realistic trajectories involve coupled motions. Early dynamics are dominated by ESIPT, which redistributes energy to drive subsequent rotations. Proton transfer to the nitrogen of MBBT weakens conjugation across the phenolic and benzotriazole units, prompting out-of-plane rotation that reduces steric hindrance and orbital overlap. Figure S46, SI, shows that this ESIPT-triggered rotation permits the MBBT system to reach the S_1_/S_0_ CI without additional energy contributions from the environment, contrary to the barriered pathway found for the “kink then rotate” trajectory. ESIPT thus indirectly drives rotation and pyramidalization, both essential for MECI formation. This agrees with prior work on 2-(2′-hydroxy-5′-methyl­phenyl)­benzo­tri­azole,^55^ though in MBBT the keto structure is not higher in energy than the MECI, likely due to *tert*-butyl steric effects during kinking.

In this work, fs-TEAS supported by MRSF-TDDFT calculations shows that, following photoexcitation, ESIPT governs the ultrafast relaxation of two structurally complex photoactive systems, BEMT and MBBT. Surprisingly, spectral and kinetic features are preserved across solvents of varying polarity and hydrogen-bonding character, contrary to the solvent-sensitive behavior typically reported for other ESIPT-active systems. Comparison with a simpler analogue of MBBT indicates that this environmental independence is largely inherent to the benzotriazole ESIPT motif rather than directly related to steric shielding caused by rich substitution. The bulky groups on MBBT are found to stabilize the proton transfer coordinate by reducing solvent access and preserving optimal donor–acceptor geometry. This, however, serves only as an auxiliary refinement, enhancing the dynamics that are already highly efficient rather than enabling them in the first place.

Our computational results predict that both BEMT and MBBT undergo ultrafast (<100 fs), barrierless ESIPT, which redistributes excitation energy into vibrational modes and guides the molecules toward S_1_/S_0_ CIs without accessing higher energy regions of the PES. ESIPT plays multiple key roles: lowering S_1_, raising S_0_, narrowing S_1_–S_0_ gaps, and facilitating structural rearrangements in MBBT. Subsequent GSIPT completes a barrierless nonradiative pathway, allowing continuous UV absorption with minimal fluorescence.

This favorable excited state landscape, alongside little dipole reorganization induced by predominantly local excitation, helps rationalize the intrinsic solvent-independent behavior. These findings place benzotriazole (and by extension triazine) chromophores as robust building blocks that can be further functionalized to tune, for example, formulation-relevant properties in the context of UV filters, while preserving the ultrafast energy dissipation across various environments. Together, the present study provides insights that can inform the design of next-generation photoprotective and photostabilizing materials.

While this work focuses on post-ESIPT relaxation dynamics, measurements with improved time resolution would allow one to directly probe solvent effects on the sub-100 fs ESIPT step and would therefore be of considerable interest.

## Supplementary Material



## Data Availability

Computational data for this letter are available via the Warwick Research Archive Portal (WRAP) at https://wrap.warwick.ac.uk/201298/. Raw experimental data are available upon request from the authors.

## Notes

Methods, UV irradiation measurements, fluorescence emission spectra and associated quantum yield, transient absorption heatmaps with associated fitting lifetimes and evolution difference spectra for UVA and UVB excitation in hexane and ethanol, photoproduct formation in BEMT, deuteration experiment for MBBT, results for the simpler analogue of MBBT, fitting residuals, instrument response function, and computational details (vertical excitations and transition densities, radial distribution functions, generation of trajectories, and potential energy surfaces) (PDF)
