# Supplementary material for: Unraveling Solvent-Independent Excited State Proton Transfer Dynamics in Sterically Substituted Photoactive Systems
Source: J Phys Chem Lett. 2026 Jun 13;17(25):7102–10. doi: 10.1021/acs.jpclett.6c01594 (PMC13312451; doi:10.1021/acs.jpclett.6c01594)
Supplement: Supplementary file 1 [file jz6c01594_si_001.pdf]

# Supporting Information:

# Unravelling solvent-independent excited state proton transfer dynamics in sterically substituted photoactive systems


Zofia Majewska [a§], Jacob Eller [b§], Kexin Pan [a], Jack Dalton [a], Bernd Herzog [c], Nicholas D. M. Hine [b] and Vasilios G. Stavros [a*]

[a] School of Chemistry, University of Birmingham, Edgbaston, Birmingham, UK

[b] Department of Physics, University of Warwick, Coventry, UK

[c] Department of Pharmaceutical Sciences, Universität Basel, Basel, Switzerland

* Email: v.stavros@bham.ac.uk

§ *Z.M. and J.E. contributed equally to this work*

## 1. Experimental

### 1.1. Methods

**Sample preparation**. Tinosorb® S (>98% BEMT) and Tinosorb® M (48.0 - 52.0% MBBT, 36.9 - 45.7% Water, 6.0 - 10.0% Decyl Glucoside, 0.2 - 0.6% Propylene Glycol, 0.1 - 0.5% Xanthan Gum by weight) were provided by BASF (Ludwigshafen, Germany) and used without further purification. Tinuvin® P (97% 2-(2'-hydroxy-5'-methylphenyl)benzotriazole, referred to as Tinuvin P hereafter) was purchased from Sigma-Aldrich and used without further purification. The stock solutions were made to ~100 µM in ethanol (absolute, Fisher Scientific) and hexane (>= 95%, Fisher Scientific) and sonicated for > 1 hour to ensure sufficient solvation. The samples were checked for presence of aggregates using a dynamic light scattering (DLS) experiments (see Figures S15 and S16).

**Steady-state absorption and emission**. The samples were prepared by diluting the stock solution to achieve 0.2 absorbance in a 1 cm path-length quartz cuvette (Hellma). The absorption spectra of BEMT and MBBT solutions in hexane and ethanol were recorded using a Cary 60 spectrometer (Agilent Technologies). The emission spectra and quantum yield were measured using an Edinburgh Instruments FS5 spectrofluorometer with the SC-30 integrating sphere module. The excitation wavelengths were set to the absorption maximum ($\lambda_{max}$) and a 1 cm pathlength quartz cuvette was used. Experiments were carried out both in air and under nitrogen, achieved by bubbling nitrogen gas through the sample for 10 minutes.

**Femtosecond transient electronic absorption spectroscopy**. The samples were flowed through a demountable liquid cell (Harrick Scientific Products Inc.) consisting of two 25 mm $CaF_2$ windows (thickness: 1 mm front, 2 mm back) separated by two poly(tetrafluoroethylene) spacers, which defined optical path length of the sample. The thickness of the spacers was selected to give an optical density (OD) of less than 0.5 through the flow cell (750 µm was selected for MBBT/hexane, and 950 µm for MBBT/ethanol, BEMT/hexane and BEMT/ethanol). The solutions were recirculated from a 25 mL reservoir using a peristaltic pump (Masterflex® MFLX07528-20-UK) with PTFE tubing throughout, and the flow cell was continuously translated in the plane perpendicular to the incoming beams to ensure that fresh sample is investigated with each pump−probe pulse pair.

A commercially available Ti-sapphire regenerative amplified laser system (Spectra-Physics, Ascend 60 pumped Spitfire Ace) seeded by a Mai Tai (Spectra-Physics) produced a 7 W beam centred at 800 nm with a 1 kHz repetition rate and ∼35 fs pulse width. The 7W beam was split into multiple lower power beams, two ∼1 W beams were used to generate the pump and probe beams for these experiments. One of these seeds an optical parametric amplifier (Light Conversion, TOPAS-C) to produce the pump beam, set to the $\lambda_{max}$ of the samples in both UVA and UVB regions.

Every other pump pulse was blocked using a chopper to achieve a ‘pump on’ and ‘pump off’ signal, from where the change in OD of the probe can be measured (Equation S2). The power of the pump pulse at the sample was selected, using a neutral density filter, to be ~0.25 mW. The pump beam is focused ~5-10 cm behind the sample by a $CaF_2$ lens ($f$ = 500 mm) to give a beam diameter of ~300-500 μm at the sample region and to ensure diameter of the pump beam is greater than that of the probe.

The other 1 W beam is further split into 0.95 and 0.05 W beams using a beam splitter, and the 0.05 W fraction is used in the experimental probe line. Pump–probe delays ranging from −1 ps to 1.8 ns are achieved by translating a hollow gold retroreflector (Edmund Optics) on a motorized optical delay line (Physik-Instrumente, M-531.DD) in the 0.05 W 800 nm beam path. The relative polarisation of the 800 nm probe pulses is adjusted to be at ‘magic’ angle (54.7°) with respect to the pump beam using a half-wave plate to avoid the dynamical consequences arising from the molecular reorientation.[1] The beam is attenuated using a neutral density filter and an iris, and focused using a bi-convex lens ($f$ = 5 cm) into a $CaF_2$ window (Eksma Optics, 25.4 mm diameter, 2mm thickness, orientation [001]) which is constantly translated vertically using a motorised stage (Newport, MFA-PPD) to generate the white light continuum (WLC, 320-740 nm) probe pulse. The $CaF_2$ window was replaced with a sapphire window (Eksma Optics, 25.4 nm diameter, 2 mm thickness) to generate a 650-950 nm probe for the red-shifted probe measurements.

The WLC is collimated using a UV-enhanced aluminium concave mirror ($f$ = 10 mm) and then focused into the sample by a second concave mirror ($f$ = 10 cm). The WLC transmitted through the sample is collimated again using a lens ($f$ = 10 cm), and the residual 800 nm fundamental beam is removed using an 800 nm notch filter (replaced with a long pass filter for red-shifted probe measurements; Thorlabs, 25 mm diameter, KG3 Coloured Glass Filter). The beam then passes through a wire-grid polariser to filter through the residual pump beam, reducing pump scatter in the data. The WLC beam is attenuated using a neutral density filter to not saturate the detector. Finally, the light is focused into a fibre optic cable using a focusing lens ($f$ = 45 mm). Changes in optical density (Equation S2) are registered using a fibre-coupled spectrometer (Avantes Ltd, dual channel Avaspec-Fast, ULS1650F-2-USB2). Control of $CaF_2$ and sample translation stages, data acquisition and the calculation of difference spectra are performed using a custom-built LabVIEW program.

**Data processing**. The transient absorption spectra were fitted with a sequential global fitting model in the software package Glotaran.[2] The lifetimes obtained correspond to evolution-associated difference spectra (EADS) that represent the evolving spectral features; fitting residuals assess the quality of the fit (Figure S17, SI). The instrument response was estimated from solvent scans, whereby an averaged

kinetic trace at a particular wavelength range was fitted using a basic or modified Gaussian function (see Section 1.4.5. for further details), where the full-width half-max (FWHM) corresponds to the shortest timescale that can be resolved (Figures S18, S19 and S20, SI). The transient electronic absorption heatmaps were chirp corrected using the software package KOALA, which models the dispersion curve as a third order polynomial.[3]

### 1.2. UV irradiation measurements

Steady-state solar irradiation measurements are used to monitor the long-term photostability and efficiency of BEMT and MBBT by simulating sunlight exposure that would be experienced in practical use.[4] The sample was irradiated for up to 2 hours by a solar simulator (Oriel LCS-100, Spectra-Physics spectrum shown in Figure S1) with the sample positioned such that the irradiance was equal to approximately 1000 W/m$^2$ (corresponding to the irradiance on the surface of the Earth on a clear summer day). Absorption spectra of a system (UV filter dissolved in solvent) were recorded using a Cary 60 spectrometer (Agilent Technologies) at various time intervals to monitor the photodegradation of the sample.

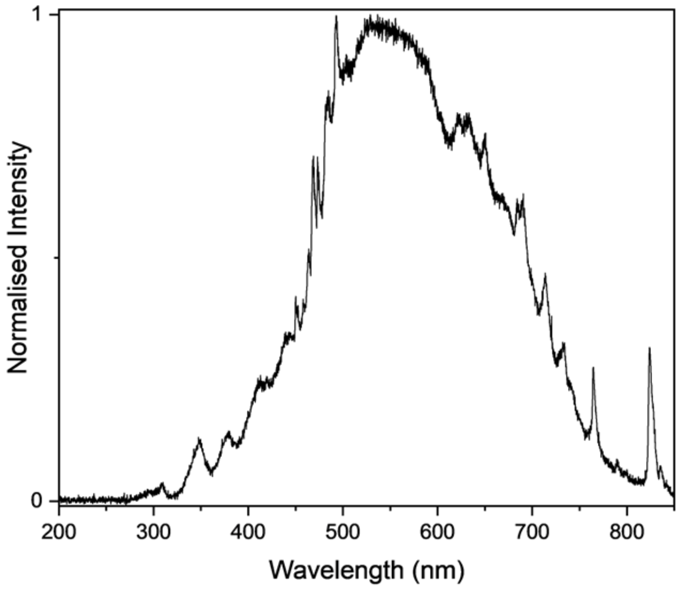


**Figure S1.** Spectrum of light produced by the solar simulator lamp (Oriel LCS-100, Spectra-Physics).

Area under the curve index (AUCI) is the metric commonly used for quantifying the photostability of UV filters.[4] It is defined as the ratio of AUC of the absorption spectrum after and before 2 hours' solar irradiation[5] over a specified wavelength range:

$$\mathrm{AUCI} = \frac{\mathrm{AUC}_{\mathrm{after}}}{\mathrm{AUC}_{\mathrm{before}}} \qquad \textbf{Equation S1.}$$

As an industry standard, a sunscreen is considered photostable if the AUCI is higher than 0.80 between 290 and 400 nm.[4]

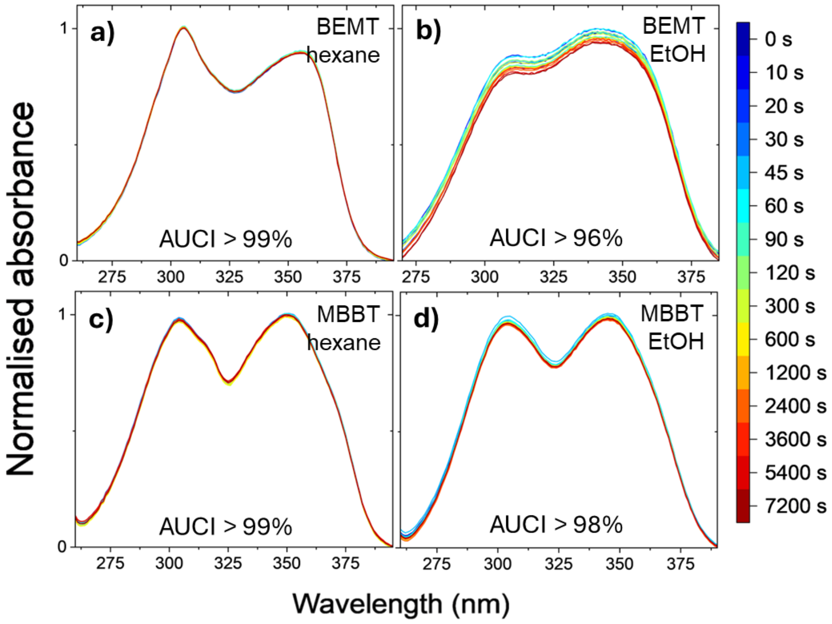


**Figure S2.** Absorption spectra for **a)** BEMT in hexane, **b)** BEMT in ethanol, **c)** MBBT in hexane and **d)** MBBT in ethanol. Corresponding AUCI values are labelled.

The corresponding AUCI values are calculated for both molecules in hexane and ethanol. Figure S2 shows the absorption spectra throughout irradiation. Minimal photodegradation is observed for both molecules maintaining >99% absorbance (between 290-400 nm) in hexane, following 2 hours of irradiation. The same measurement in ethanol yields 98% retention for MBBT and 96% for BEMT. On that account, they compete with other highly photostable sunscreen molecules on the market[6] and surpass many natural UV protectants, such as mycosporine-like amino acids.[7]

### 1.3. Fluorescence emission spectra and fluorescence quantum yield measurements

Steady-state fluorescence emission data was collected for MBBT and BEMT in air and under nitrogen in a 1 cm pathlength quartz cuvette (Figure S3). Nitrogen was bubbled through the system to remove oxygen, reducing the triplet oxygen available that quenches triplet states, causing potential phosphorescence to be more observable (if present). In each case the spectra look very similar in both conditions, implying there is no phosphorescence (i.e., no triplet states are populated). Samples bubbled with nitrogen showed higher emission counts, however that might be because upon bubbling with nitrogen, some of the solvent evaporated, therefore the concentration of the compound was higher, which resulted in higher counts. The emission spectra in hexane show more distinct features comprising of the two bands (when the Raman scatter peak is ignored), closely mirroring the corresponding absorption spectra of BEMT and MBBT. No such features are observed in ethanol. A fluorescence quantum yield was measured for each sample in air and under nitrogen. In each case, it was close to 0%, therefore negligible.

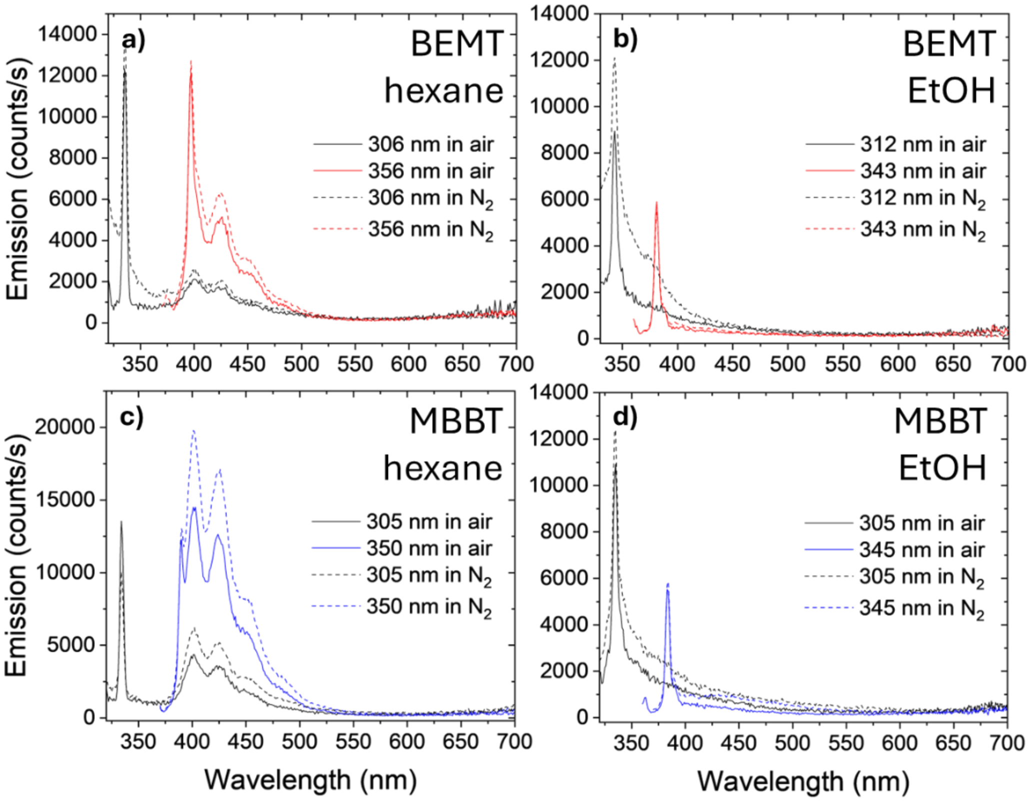


**Figure S3.** Fluorescence emission spectra collected for a) BEMT in hexane, b) BEMT in ethanol (EtOH), c) MBBT in hexane and d) MBBT in EtOH.

### 1.4. Femtosecond Transient Electronic Absorption Spectroscopy (fs-TEAS)

#### 1.4.1. Principles of the technique

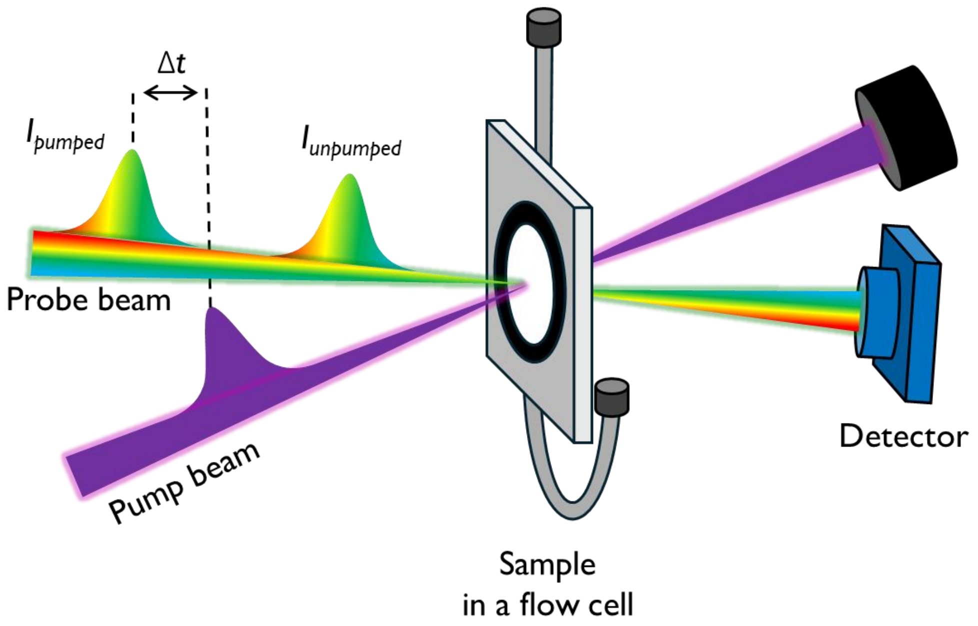


**Figure S4.** Schematic diagram of the fs-TEAS set-up.

The pump-probe setup involves two laser pulses; the probe pulse is a white light continuum spanning from ~320 to 720 nm and the pump pulse is set to $\lambda_{max}$ of the molecule (Figure S4). A single measurement starts with a probe pulse, $I_{unpumped}$, which effectively takes the absorption spectrum of a sample before photoexcitation. The pump pulse then photoexcites the molecule, mimicking the effect of UV light exposure, and a second probe pulse, $I_{pumped}$, measures how the molecule's absorption spectrum changes over time. The moment when both laser pulses arrive at the same time marks the beginning of molecular dynamics and is referred to as time zero, $\Delta t = 0$. By varying the time delay, $\Delta t$, between the pump and probe pulse, it is possible to monitor how the molecule evolves after photoexcitation. The results of such measurements are reported as the change in optical density, ΔOD, defined by the following equation:

$$\Delta \mathrm{OD}(\lambda, \Delta t) = \log_{10}\left(\frac{I_0(\lambda)}{I_t(\lambda, \Delta t)}\right), \qquad \textbf{Equation S2.}$$

where $\lambda$ is the probe wavelength, $\Delta t$ is the pump-probe time delay, $I_0$ is the transmitted intensity of the probe pulse before photoexcitation with the pump, and $I_t$ is the transmitted intensity of the probe pulse following photoexcitation with the pump.

### 1.4.2. Additional results

#### 1.4.2.1. UVB excitation fs-TEAS

fs-TEAS data for each sample was collected at both $\lambda_{max}$ wavelength values. Figure S5 shows the results for the measurements collected for the pump wavelengths in the UVB region, with the corresponding evolution associated difference spectra (EADS) extracted from the global sequential fitting of the data. Table S1 contains the lifetimes extracted from the global sequential fitting of the data collected for BEMT and MBBT pumped at UVB wavelengths. Figure S6 shows the results for the measurements collected for the pump wavelengths in the UVA region, presented as a heatmap, with the corresponding EADS extracted from the fitting.

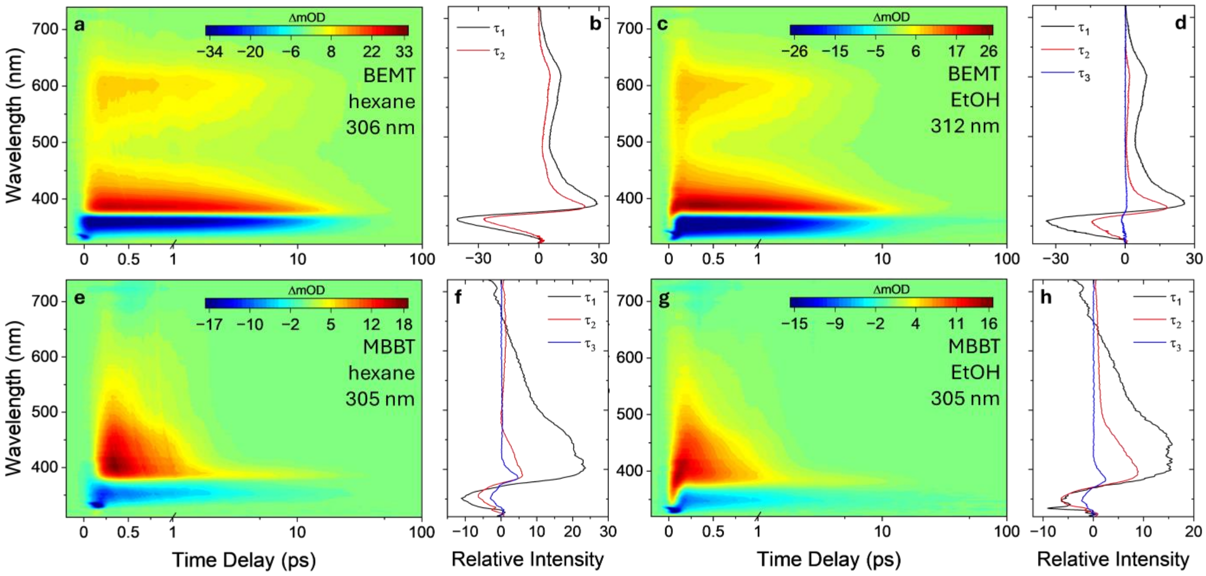


**Figure S5.** False colour heatmaps, showing collected fs-TEAS data for **a)** BEMT in hexane and **c)** BEMT in ethanol, and **e)** MBBT in hexane, and **g)** MBBT in ethanol (photoexcited at their corresponding UVB excitation wavelengths). Results of the global fit performed on the data, presented as EADS, for **b)** BEMT in hexane and **d)** BEMT in ethanol, and **f)** MBBT in hexane, and **h)** MBBT in ethanol (corresponding lifetimes are presented in Table S1 below). Time delay is linear up to 1 ps and logarithmic thereafter.

**Table S1.** Results of the global sequential fitting for BEMT and MBBT pumped at UVB wavelengths.

| Molecule | BEMT | | MBBT | |
|---|---|---|---|---|
| Solvent/Wavelength | **Hexane/306 nm** | **Ethanol/312 nm** | **Hexane/305 nm** | **Ethanol/305 nm** |
| $\tau_1$ / ps | 2.63 ± 0.06 | 2.26 ± 0.06 | 0.23 ± 0.06 | 0.25 ± 0.07 |
| $\tau_2$ / ps | 13.2 ± 0.07 | 6.69 ± 0.09 | 0.74 ± 0.06 | 0.73 ± 0.07 |
| $\tau_3$ / ps | | > 1 800 | 10.8 ± 0.2 | 12.2 ± 0.4 |

### 1.4.2.2. UVA excitation fs-TEAS

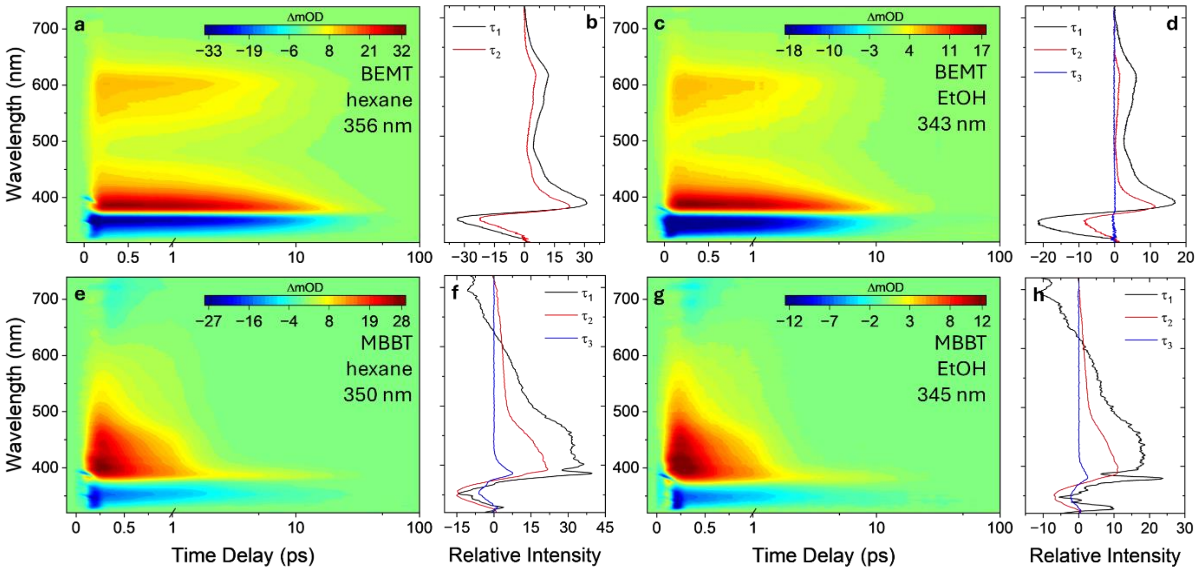


**Figure S6.** False colour heatmaps, showing collected fs-TEAS data for **a)** BEMT in hexane, **c)** BEMT in ethanol, **e)** MBBT in hexane, and **g)** MBBT in ethanol (photoexcited at their corresponding UVA excitation wavelengths). Results of the global fit performed on the data, presented as EADS, for **b)** BEMT in hexane, **d)** BEMT in ethanol, **f)** MBBT in hexane, and **h)** MBBT in ethanol (corresponding lifetimes are presented in Table 1 the main manuscript).

### 1.4.2.3. Measurements in acetonitrile.

To decouple the effect of solvent polarity and proticity on the dynamics of MBBT and BEMT, a third, polar but aprotic, solvent (acetonitrile) was chosen for further controlled comparison. Despite the experimental challenges associated with solubility, the dynamics observed in acetonitrile closely resemble those in ethanol and hexane (Figure S7), indicating that proticity does not significantly affect the ESIPT-related relaxation. Similarly to the data collected initially, there is a minor photoproduct present for BEMT in acetonitrile at the shorter pump wavelength (Figure S7d), supporting our conclusions that its formation is strongly excitation energy dependent.

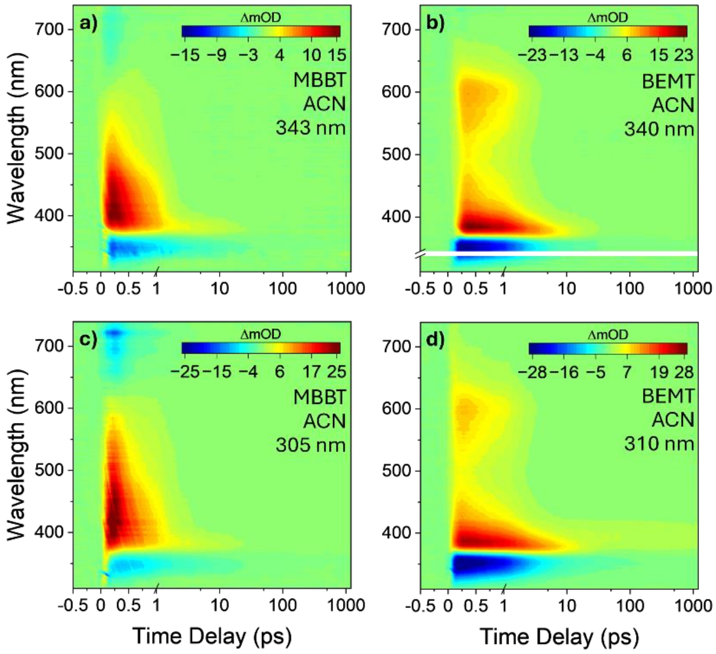


**Figure S7.** False colour heatmaps, showing collected fs-TEAS data in acetonitrile for **a)** MBBT and **b)** BEMT photoexcited at their corresponding UVA excitation wavelengths, and **c)** MBBT and **d)** BEMT photoexcited at their corresponding UVB excitation wavelengths. Time delay is linear up to 1 ps and logarithmic thereafter.

### 1.4.2.4. Red-shifted probe fs-TEAS measurement

To investigate the presence of keto stimulated emission in BEMT, the experimental setup was altered to generate a probe wavelength range of 650-950 nm. The data reveals excited state absorption spanning a broad wavelength range (Figure S8), which likely obscured any potential stimulated emission.

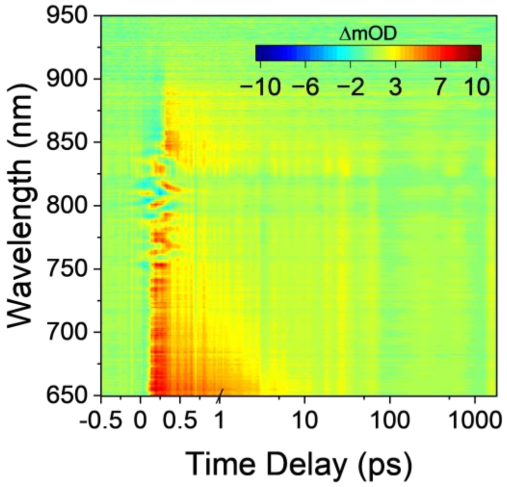


**Figure S8.** False colour heatmap, showing collected fs-TEAS data for BEMT in hexane photoexcited at 306 nm using the red-shifted probe setup.

### 1.4.2.5. Photoproduct formation in BEMT

BEMT in hexane pumped at UVB $\lambda_{max}$ and BEMT in ethanol pumped at both UVA and UVB $\lambda_{max}$'s exhibit a long-lived feature lasting beyond the experimental time window. To investigate the nature of this feature, a ground state absorption spectrum of the measured solution was taken before and after single wavelength irradiation (SWI) with a pump beam used in a fs-TEAS measurement and the sample was translated in a 1 mm cuvette. The difference between the two was then calculated by subtracting the absorption spectrum taken before the measurement from that taken after the measurement. This revealed a positive absorption feature around 400 nm, which was then compared with the final time delays of the fs-TEAS scan (Figure S9). There was good agreement between this difference spectrum and the final time delay, indicating formation of a photoproduct.

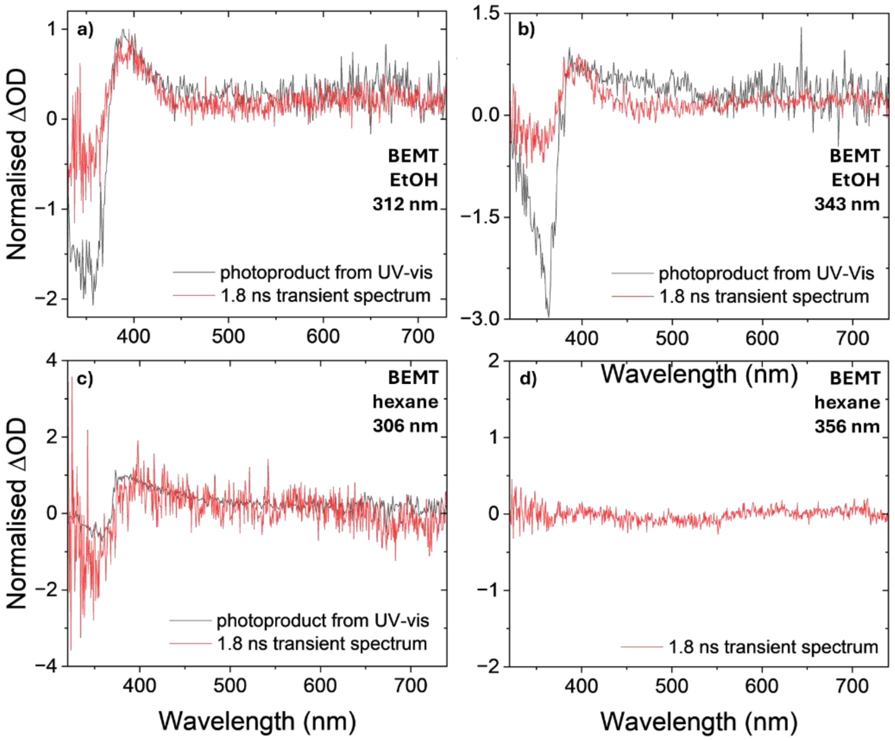


**Figure S9.** Photoproduct identification for BEMT. Final time delay (1.8 ns) transients collected for BEMT in **a)** ethanol photoexcited at 312 nm, **b)** ethanol photoexcited at 343 nm, **c)** hexane photoexcited at 306 nm, and **d)** hexane photoexcited at 356 nm. Transients a, b and c are superimposed on the corresponding difference absorption spectrum of the sample pre- and post- SWI measurement.

Formation of this photoproduct is excitation-wavelength dependent. It was observed in both solvents following photoexcitation with the higher energy pump (UVB). Following photoexcitation with the lower energy pump (UVA), the photoproduct was formed only in ethanol where the UVA and UVB peaks are closer together (31 nm separation *versus* 50 nm in hexane). This suggests that this photoproduct originates from a minor relaxation pathway accessed from higher-lying states rather than from dynamics on the primary $S_1$ surface.

### 1.4.2.6. Data collected for Tinuvin P

Tinuvin P was investigated to assess if the presence of bulky substituents is necessary for achieving efficient, solvent independent ESIPT. Additionally, the fs-TEAS study of Tinuvin P was used to confirm that the dynamics observed for MBBT were not affected by the presence of additives in the solution during the measurement. Since the spectral features of both MBBT and Tinuvin P are near-identical, we conclude that the presence of additives does not disrupt the dynamics.

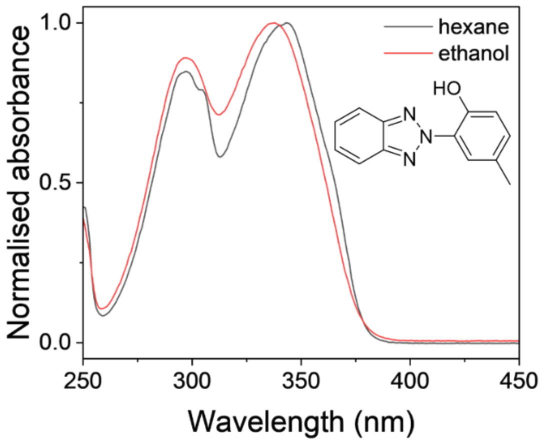


**Figure S10.** Absorption spectra of Tinuvin P in hexane (black line) and ethanol (red line), with the molecular structure displayed in the graph inset.

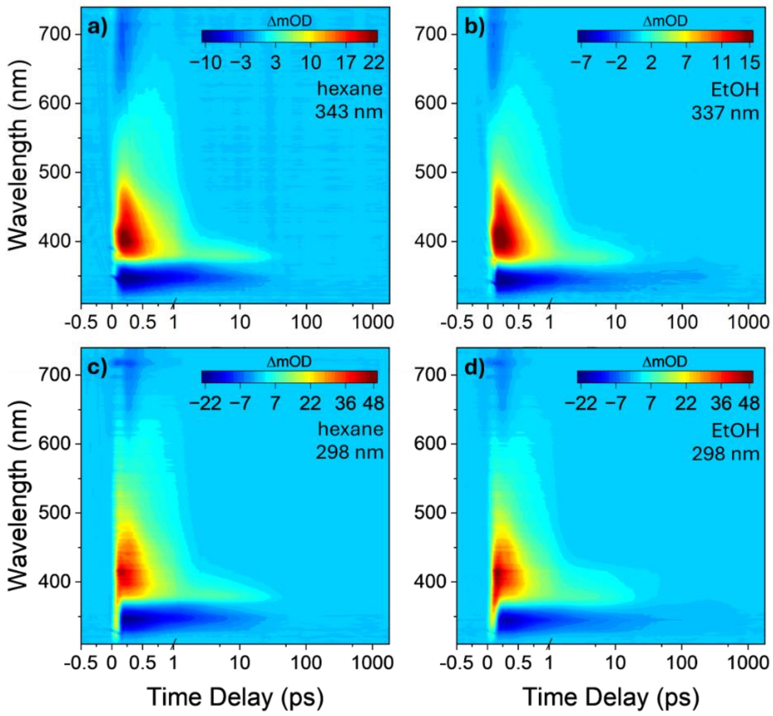


**Figure S11**. False colour heatmaps, showing collected fs-TEAS data for Tinuvin P in **a)** hexane and **b)** ethanol photoexcited at their corresponding UVA excitation wavelengths, and **c)** hexane and **d)** ethanol photoexcited at their corresponding UVB excitation wavelengths. Time delay is linear up to 1 ps and logarithmic thereafter.

### 1.4.2.7. MBBT energy barrier in the excited state proton transfer step

Proton transfer often proceeds via tunnelling of the proton through a barrier. Since the proton transfer in MBBT had to be forced in the initial TDDFT calculations, it was thought that it might have a barrier. Therefore, deuteration of the transferred proton should prolong the lifetime of the enol to keto tautomerisation due to the kinetic isotope effect.

MBBT was deuterated to see whether we could decelerate the proton transfer step and make it observable in the fs-TEAS experiment. First, the sample was dried in the oven to evaporate any water that was present. Next, it was dissolved in 9 mL of deuterated chloroform ($CDCl_3$) and sonicated to ensure complete solvation. 1 mL of deuterated water ($D_2O$) was added to this solution to provide a source of labile deuterium to be exchanged with the proton on the -OH group. Deuteration was confirmed by NMR spectroscopy (see Figure S13 and S14).

Deuterated MBBT in $CDCl_3$ was measured in fs-TEAS, photoexcited at its $\lambda_{max}$ (348 nm) and 390 nm (to minimise excess energy given to the molecule), and compared against the data of MBBT in $CHCl_3$. For both wavelengths, we did not observe any isotope effect (see Figure S12). The tautomerisation step was completed within the instrument response time (Figure S20). This suggests that the barrier is negligible (in keeping with our calculations, see Section 2.5.).

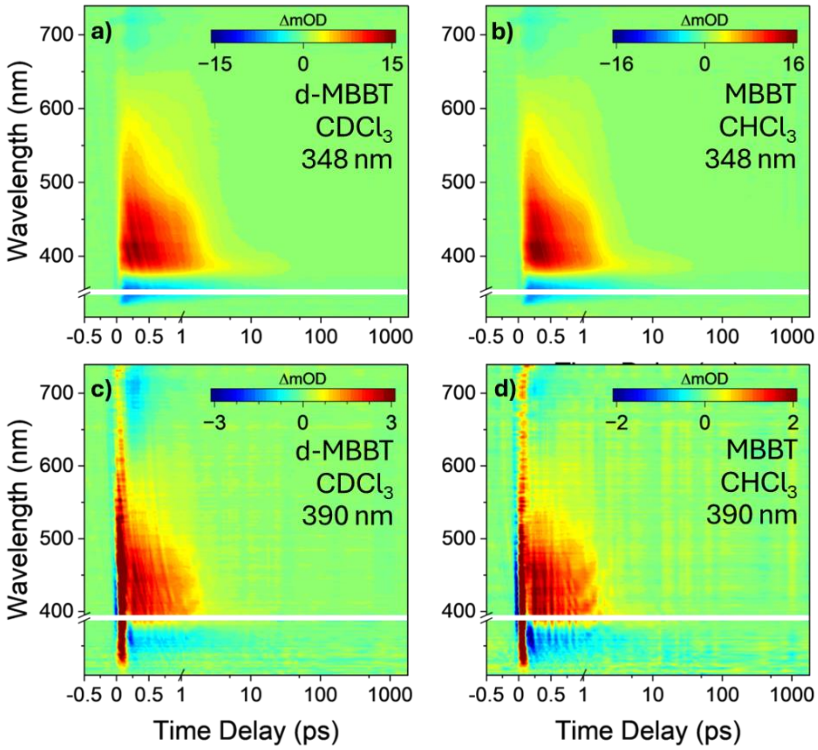


**Figure S12**. False colour heatmaps, showing collected fs-TEAS data for **a)** deuterated MBBT in $CDCl_3$ and **b)** MBBT in $CHCl_3$ (control) photoexcited at 348 nm, and **c)** deuterated MBBT in $CDCl_3$ and **d)** MBBT in $CHCl_3$ (control) photoexcited at 390 nm. Time delay is linear up to 1 ps and logarithmic thereafter.

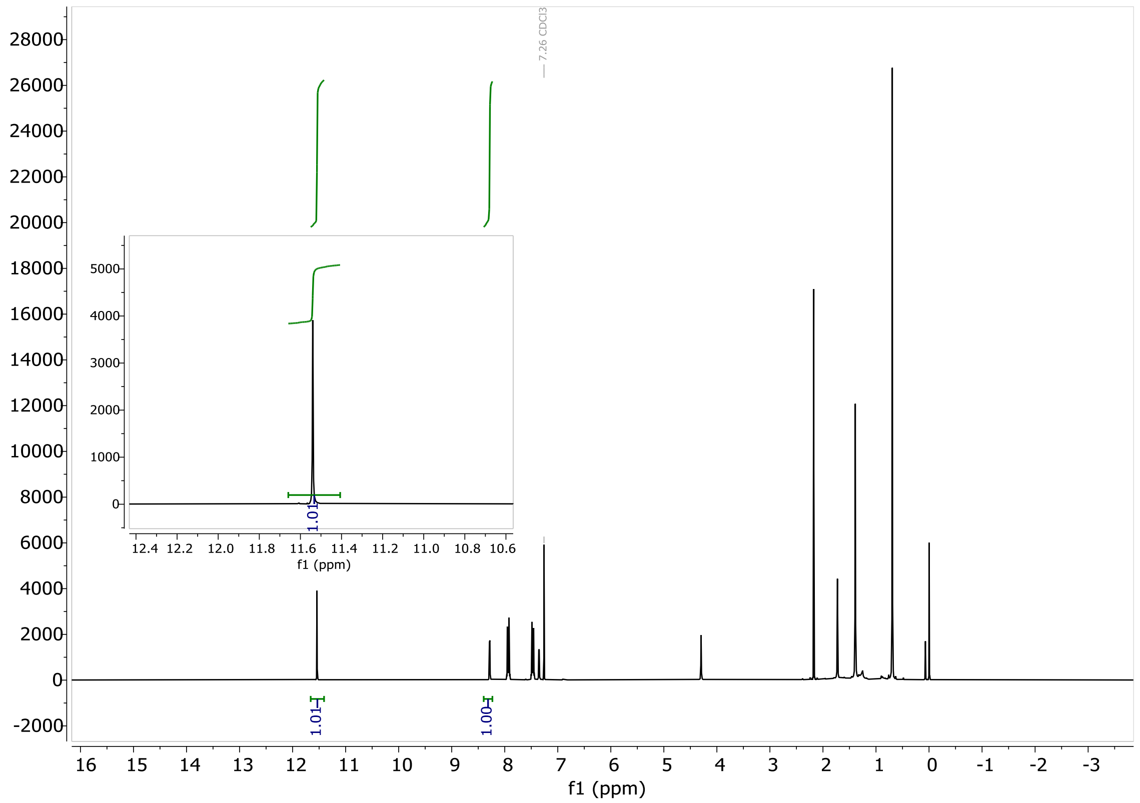


**Figure S13.** $^{1}$H-NMR spectrum of MBBT pre-deuteration (300 MHz, $CDCl_3$). The spectrum inset shows the hydroxyl proton at 11.55 ppm.

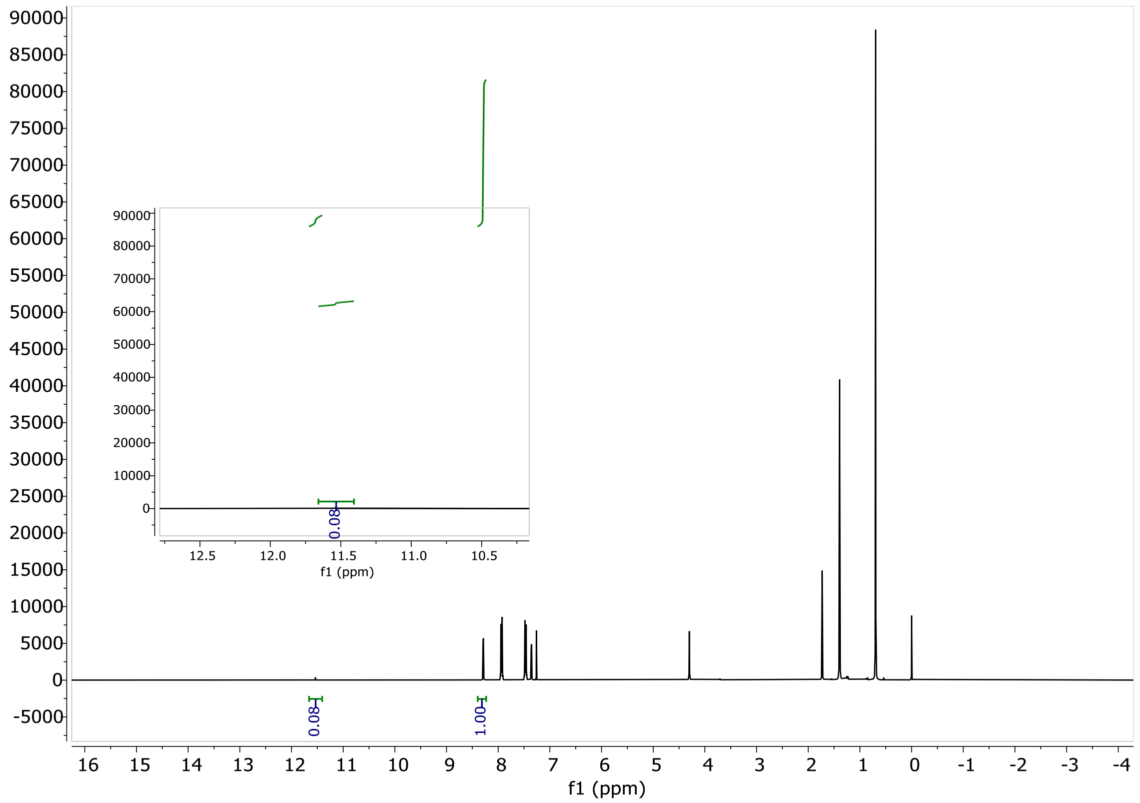


**Figure S14.** $^{1}$H-NMR spectrum of MBBT post-deuteration (300 MHz, $CDCl_3$). The spectrum inset shows near complete disappearance of the hydroxyl proton at 11.55 ppm.

### 1.4.3. Checking for aggregates

Due to poor solubility of MBBT and BEMT in ethanol, the samples were assessed for presence of large aggregates or nanoparticles in solution. A dynamic light scattering (DLS) measurement was performed on solutions of MBBT and BEMT in ethanol. The data was compared against the data of MBBT in water where it is known to form nanoparticles (Figure S15).[8] As expected, data for MBBT in water showed presence of nanoparticles of varying sizes, mostly 102 ± 27 nm and 290 ± 120 nm (values comparable with literature), whereas the peak ~4871 nm corresponds to dust particles in solution. Data for MBBT in ethanol and BEMT in ethanol confirmed absence of any aggregates or nanoparticles (Figure S16).

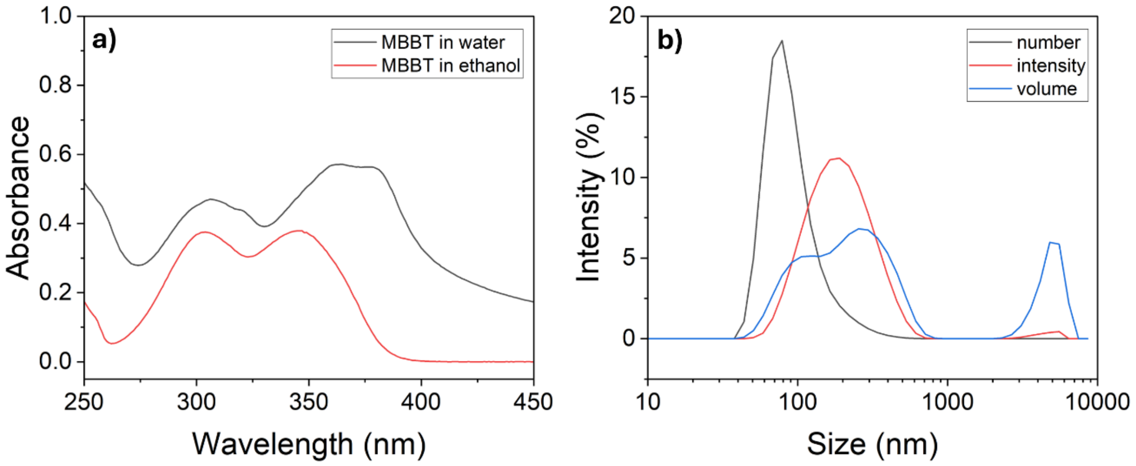


**Figure S15.** Data for nanoparticles of MBBT in water: **a)** absorption spectra of MBBT in water (black line) and ethanol (red line) - note the change in shape; **b)** DLS results confirming the presence of nanoparticles of MBBT in water.

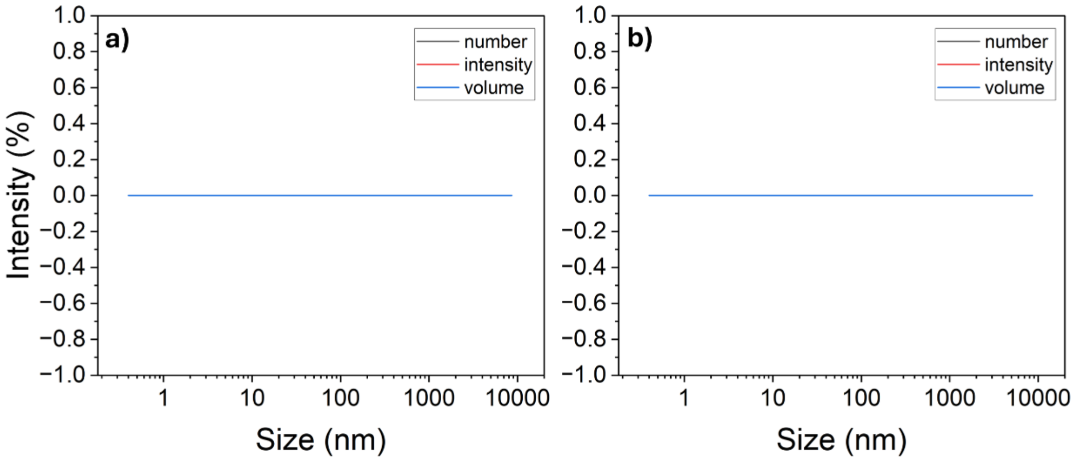


**Figure S16.** DLS results for **a)** MBBT in ethanol, **b)** BEMT in ethanol. Measurement showed no particles.

### 1.4.4. Fitting residuals

The quality of the fitting was determined by comparison of the residuals between the raw data and the fit, where residuals close to 0 correspond to a good quality fit. The fitting for BEMT was performed using the data prior to chirp correction (Figure S17a-d). However, for MBBT that could not be achieved as some spectral features were not being captured in the fitting. Therefore, chirp corrected data was fitted instead (Figure S17e-h). To assess if such approach introduces any bias to the fitting, a dataset for chirp corrected BEMT data was fitting and compared to the non-chirp corrected results. Both methods of fitting gave very similar results.

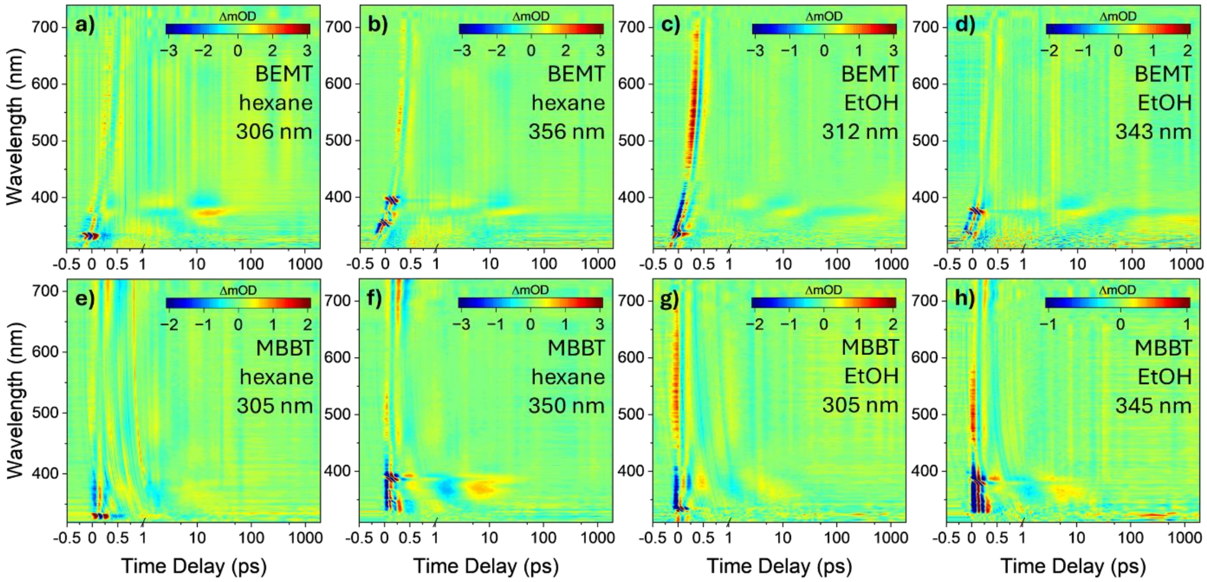


**Figure S17.** Residuals of the global sequential fitting for: **a)** BEMT in hexane at 306 nm, **b)** BEMT in hexane at 356 nm, **c)** BEMT in ethanol at 312 nm, **d)** BEMT in ethanol at 343 nm, **e)** MBBT in hexane at 305 nm, **f)** MBBT in hexane at 350 nm, **g)** MBBT in ethanol at 305 nm, and **h)** MBBT in ethanol at 345 nm.

### 1.4.5. Instrument response

To obtain the instrument response function (IRF), a fs-TEAS measurement was carried out for every solvent and for every pump wavelength used in each measurement. An averaged kinetic trace at a wavelength region where dynamics are evident is then plotted, and the dataset is fitted using a basic Gaussian function ($g(x)$, Equation S3, where $B = 0$ and $C = 0$) or modified Gaussian function ($g(x)$, Equation S3, where $B \neq 0$ and $C \neq 0$), as specified on Figures S18, S19 and S20. The value of the FWHM is quoted as the instrument response and corresponds to the fastest possible dynamical lifetime observable. Half FWHM is then quoted as the smallest possible error in fitting lifetimes.

$$g(x) = y_0 + (A + Bx + Cx^2) \times \exp\left(-\left(\frac{(x - x_0)}{\frac{\text{FWHM}}{1.67}}\right)^2\right) \qquad \textbf{Equation S3.}$$

In Equitation S3, $A$, $B$ and $C$ are coefficients of the polynomial, FWHM is the full-width half-max of the Gaussian peak, $y_0$ is the y-offset and $x_0$ is the centre of the Gaussian peak.

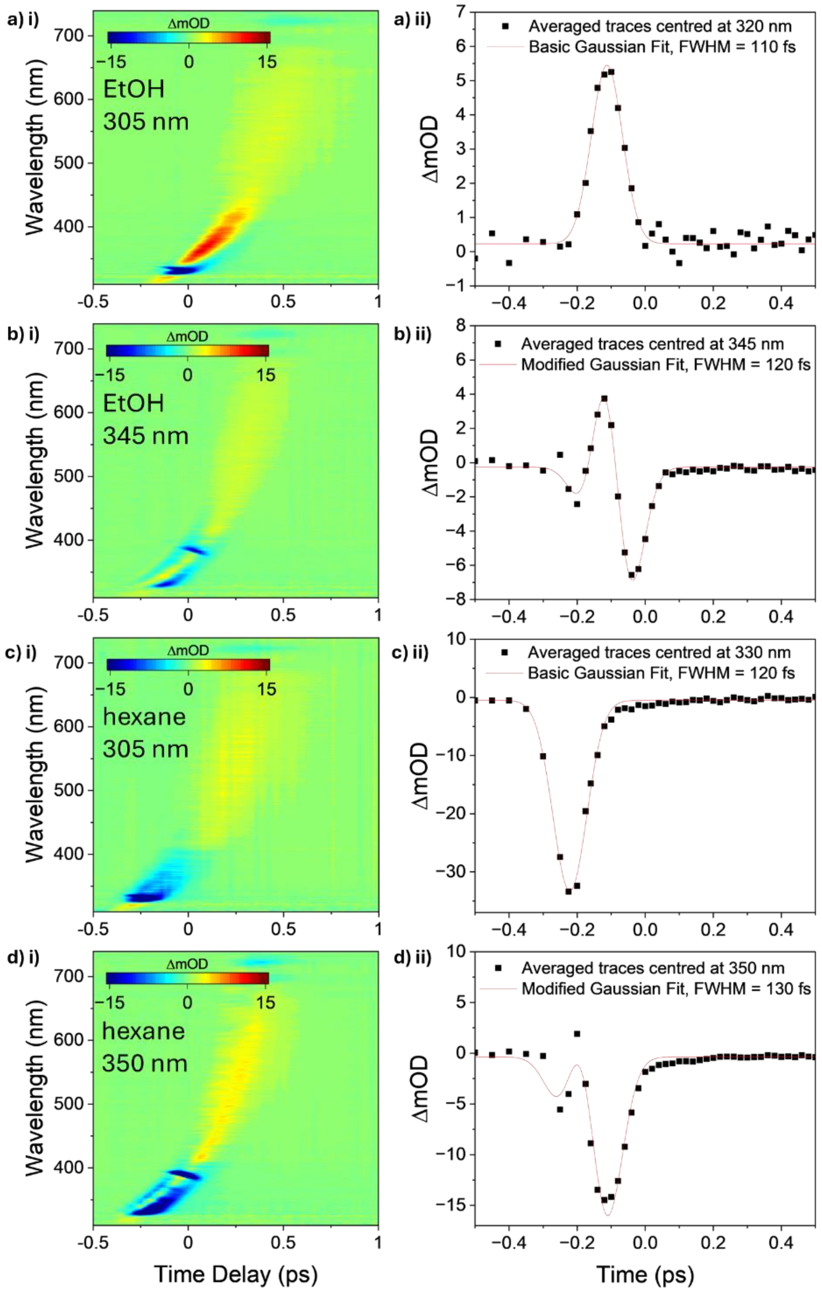


**Figure S18.** fs-TEAS heatmaps of pure solvent **(i)** and the corresponding averaged traces at the selected probe wavelength of the solvent-only time-zero response probed at specified excitation wavelengths (**ii**, black squares) for: **a)** ethanol at 305 nm, **b)** ethanol at 345 nm, **c)** hexane at 305 nm, **d)** hexane at 350 nm. All transients are fitted with Equation S3 (**ii**, red line) to determine the instrument response, where the FWHM is quoted as the IRF.

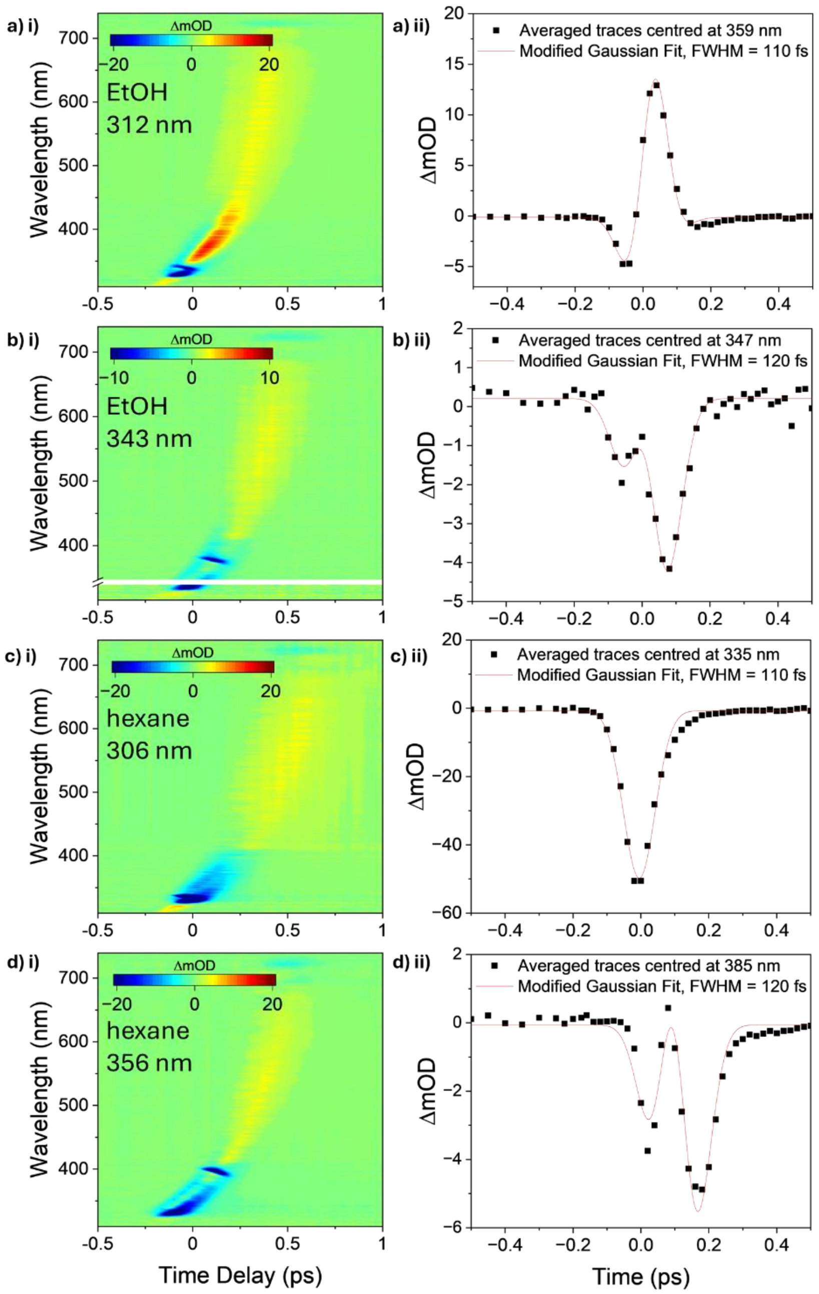


**Figure S19.** fs-TEAS heatmaps of pure solvent **(i)** and the corresponding averaged traces at the selected probe wavelength of the solvent-only time-zero response probed at specified excitation wavelengths (**ii**, black squares) for: **a)** ethanol at 312 nm, **b)** ethanol at 343 nm, **c)** hexane at 306 nm, **d)** hexane at 356 nm. All transients are fitted with Equation S3 (**ii**, red line) to determine the instrument response, where the FWHM is quoted as the IRF.

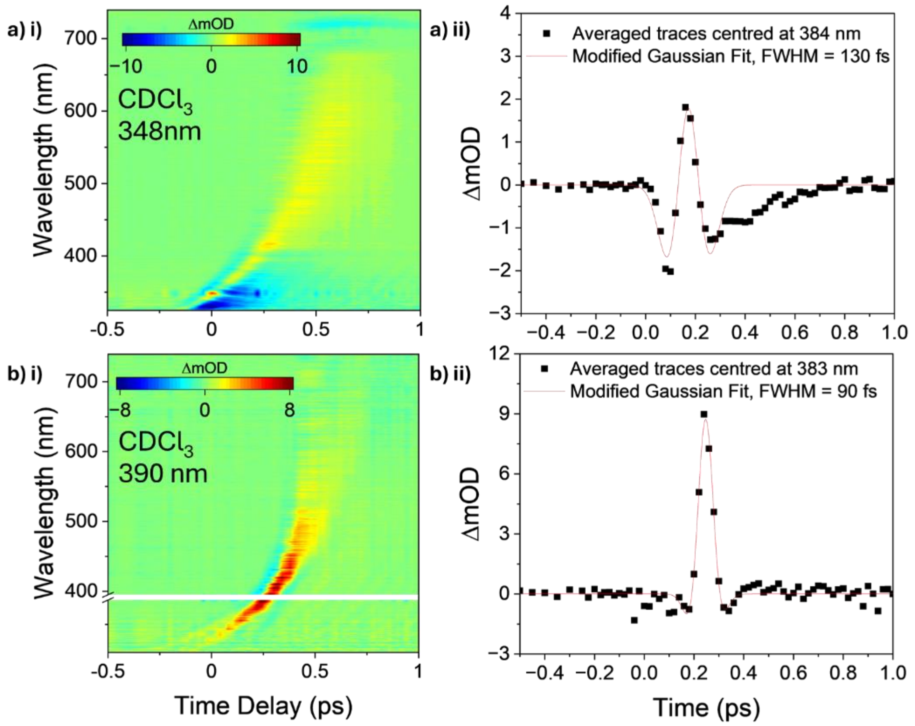


**Figure S20.** fs-TEAS heatmaps of pure solvent **(i)** and the corresponding averaged traces at the selected probe wavelength of the solvent-only time-zero response probed at specified excitation wavelengths (**ii**, black squares) for: **a)** $CDCl_3$ at 348 nm, **b)** $CDCl_3$ at 390 nm. All transients are fitted with Equation S3 (**ii**, red line) to determine the instrument response, where the FWHM is quoted as the IRF.

## 2. Computational results

### 2.1. Review of available methodologies in the vicinity of conical seams

Conical seams are *F*-2 dimensional (where *F* is the number of vibrational degrees of freedom) regions of degeneracy between potential energy surfaces (PESs) associated with different adiabatic states—each point within a conical seam is a conical intersection (CI).[9] Following electronic excitation, if a system can access $S_n/S_0$ CI geometries, internal conversion between adiabatic states can occur, facilitating non-radiative excited state relaxation.

Many methods suffer from the fact that they treat ground and excited states differently—in the case of LR-TDDFT, the Kohn-Sham ground state is calculated variationally, while excited states are obtained by solving the Casida equation.[9-11] This means that calculated ground states and excited states do not share a common variational principle; the inconsistent treatment of ground and excited states does not provide the correct derivative (nonadiabatic) coupling and cannot reproduce the correct topology of a CI. This results in a systematic error where the conical seam is incorrectly calculated to be *F*-1 dimensional and often culminates in the unphysical ordering of state energies, with the required ground to excited state degeneracy not observed. Historically, the most common approach to improve the treatment of CIs has been to use multi-reference wavefunction methods such as CASSCF/CASPT2, where the wavefunctions for adiabatic states are represented as linear combinations of multiple Slater determinants/electron configurations.[52-55] However, these methods have two major limitations: i) they require the selection of an active space of electron configurations, which is difficult to carry out effectively for a system that will be accessing multiple regions of phase space with differing character of the electronic states, and ii) they are very computationally expensive, with this expense scaling significantly with system size and the size of the active space. Thus, these methods are unsuitable for elucidating the excited state behaviour of large molecules such as BEMT and MBBT. Instead, we look to adapted forms of TDDFT, which generally scale much more favourably with system size than wavefunction methods. Spin-flip (SF-) TDDFT is an approach that treats ground and excited singlet states on equal footing.[56] It does this by calculating all adiabatic state properties via single electron spin-flipped excitations from an open-shell triplet sacrificial reference state, which is obtained by augmenting the closed-shell ground state singlet electron configuration by moving one spin-flipped highest occupied molecular orbital (HOMO) electron to the HOMO+1 Kohn Sham orbital. While SF-TDDFT results in more realistic coupling between the calculated ground and excited singlet states, which largely remedies the discussed problems with conical seam dimensionality and energetic state ordering, it does introduce a new issue: spin contamination. Depending upon which electron is flipped to generate the reference state, one of two overlapping yet discrete sets of excited state configurations can be accessed through single electron spin flip excitations, so the excitation manifold is not spin-

complete. In some regions of phase space, this can cause SF-TDDFT to calculate properties for non-physical spin-impure states, where the desired singlet state is significantly contaminated by the character of a triplet state. A recent further advance on this method, mixed-reference spin flip (MRSF-) TDDFT, is able to mostly remove this contamination by using a non-physical reference triplet state which is an equally weighted combination of the two possible high-spin triplet states, permitting the inclusion of both sets of excited electron configurations.[57,58] The computational cost associated with MRSF-TDDFT is greater than SF-TDDFT, but still scales much more efficiently with system size than multireference wavefunction methods. Hence, in this work SF-TDDFT is used to carry out initial minimum energy CI (MECI) searches, while MRSF-TDDFT is used more sparingly to validate and finetune MECIs and improve the reliability of calculated energy surfaces.

### 2.2. Methods

Throughout this work, all density functional theory (DFT), linear response time dependent DFT (LR-TDDFT) and spin-flip time dependent DFT (SF-TDDFT) calculations were performed using ORCA/6.0.0, a Gaussian basis ab initio quantum chemistry package.[12, 13] The Conductor-like Polarizable Continuum Model was used to implicitly solvate solute molecules where required.[14] All mixed-reference spin-flip time dependent DFT (MRSF-TDDFT) calculations were carried out using OpenQP/1.0.[15]

Initial 3D conformers of BEMT and MBBT were generated from their Simplified Molecular-Input Line-Entry System codes using Open Babel.[16, 17] Both resulting geometries underwent a ground state relaxation in the gas phase using DFT with the PBE0 exchange correlation functional,[18] def2-TZVP basis set,[19, 20] and D3BJ dispersion correction.[21] Using the resulting geometries as inputs, the Conformer-Rotamer Ensemble Sampling Tool/3.0.2 generated conformational ensembles for each molecule, evaluating corresponding energies at the GFN2-xTB level of theory.[22-24] All conformers within 0.1 eV of the lowest energy conformer were visually assessed for their geometric similarity to the lowest energy structures: all low energy conformers are thermally populated, so any significantly different geometries must be considered. For BEMT, all identified geometries within the 0.1 eV window were structurally very similar. However, for MBBT, the 21$^{st}$ lowest energy conformer featured a different orientation of the alkyl chain attached to one of the benzene rings. Simulations stemming from the most stable and 21$^{st}$ lowest energy conformers of MBBT will be referred to as the MBBT 1 and MBBT 21 systems, respectively. The resulting two conformers of MBBT and the most stable conformer of BEMT were the starting geometries of subsequent calculations. The 'Solutes' procedure from the Explicit Solvent Toolkit for Electronic Excitations of Molecules (ESTEEM) was then used to perform LR-TDDFT calculations of the electronic transitions contributing to absorption spectra for each of the three systems.[25-27] Three different DFT and LR-TDDFT setups were used to separately carry out the procedure

on each system: i) CAM-B3LYP functional, def2-TZVP basis set, and D3BJ dispersion correction;[28] ii) ωB97X-D3BJ functional and def2-TZVP basis set;[29] and iii) B3LYP functional, def2-TZVP basis set, and D3BJ dispersion correction.[30] The BEMT, MBBT 1, and MBBT 21 geometries underwent a gas phase geometry relaxation in $S_0$—the resulting geometries are the FC geometries. Following this, the FC geometries were relaxed in implicit hexane and ethanol with the system in $S_0$. The ten lowest energy singlet-singlet transitions were then computed for the geometries relaxed in the gas phase, in implicit ethanol and in implicit hexane. The gas phase relaxed geometries were then obtained with the system in $S_1$.

The resulting geometries were relaxed in implicit hexane and ethanol in $S_1$. The FC geometry of MBBT 21 was then used to generate a geometry of 2-(2'-hydroxy-5'-methylphenyl)benzotriazole (referred to as Tinuvin P hereafter) by deleting extra atoms and capping the molecule with a hydrogen atom. The resulting Tinuvin P was then relaxed in $S_0$ in the gas phase using the ωB97X-D3BJ setup discussed above to generate its FC geometry. The FC geometries of MBBT 21 and Tinuvin P were then surrounded by boxes of solvent, heated to 300k, and allowed to equilibrate by following the procedure outlined in the 'Solvate' task in ESTEEM, using AMBER23.[25, 31] Then 1000 ps of molecular dynamics were propagated with 2 fs steps for both solvated systems, with snapshots being stored at 200-step intervals, resulting in trajectories of 2500 snapshots.

Those geometries obtained from implicit hexane geometry optimizations in the $S_1$ state using CAM-B3LYP and ωB97X-D3BJ were then used as starting geometries for MECI optimizations, looking for $S_1/S_0$ CIs. MECI optimizations were performed in implicit solvent by SF-TDDFT for each input geometry using the same sets of functionals, basis sets and dispersion corrections as were used to obtain these starting geometries. Experimental fs-TEAS spectra obtained for BEMT and MBBT in hexane and ethanol suggest that the relaxation mechanism is largely independent of the solvent environment. Hence, consideration in parallel of multiple solvents is not expected to qualitatively affect seam topology, and these CI optimizations were carried out in implicit hexane. Monitoring of $\langle S^2 \rangle$ indicated that our systems were traversing potential energy surfaces associated with spin-contaminated states; thus, the resulting geometries could not be regarded as reliable CIs. Instead, the geometries with the lowest SF-TDDFT energy gap for each exchange correlational functional were used as input geometries for MRSF-TDDFT MECI searches. For all MECI searches, the maximum number of self-consistent field iterations was set to 1000, the convergence threshold for the Direct Inversion of Iterative Subspace Error was set to $5 \times 10^{-6}$, and the def2-TZVP basis set was used. Simulations starting from geometries obtained by SF-TDDFT using CAM-B3LYP and ωB97X-D3BJ used the CAM-B3LYP and ωB97X functionals, respectively. OpenQP/1.0 does not support dispersion corrections or implicit solvation, so these effects were neglected.

Geometric differences between the FC and CI geometries of the three systems were identified and decomposed into a series of motions. For BEMT, the overall motion was broken up into an ESIPT of a phenolic hydrogen to an accepting nitrogen, and a subsequent rotation of the resulting phenyl-alkyl appendage ($\phi_S$ in Figure 3 of the main text). MBBT 1 and MBBT 21 exhibited almost identical changes in geometry, which we decomposed into three motions: an initial ESIPT of a phenolic proton to an accepting nitrogen, and a subsequent rotation ($\phi_M$ in Figure 4 of the main text) and pyramidalisation of the benzotriazole moiety ($\theta_1$ and $\theta_2$ in Figure 4 of the main text). To validate that these CI geometries were energetically accessible, we carried out relaxed scans along these motions. The procedures used to generate the corresponding starting geometries are detailed in SI, Section 2.5.1. Constrained geometry relaxations—fixing those bond lengths, dihedral angles, and angles corresponding to their positions along the collective variables—were used to identify low energy pathways towards the CIs. These relaxations were carried out using LR-TDDFT (with the same settings as used in earlier CAM-B3LYP and ωB97X-D3BJ calculations), and the final geometries were also evaluated with MRSF-TDDFT. Finally, geometries identified as MECIs were relaxed in vacuum in the ground state using the same CAM-B3LYP and ωB97X-D3BJ setups with DFT to assess whether the systems were able to return to their FC geometries following internal conversion.

## 2.3. Excitation calculations for Franck-Condon geometries

### 2.3.1. Implicit solvent effects

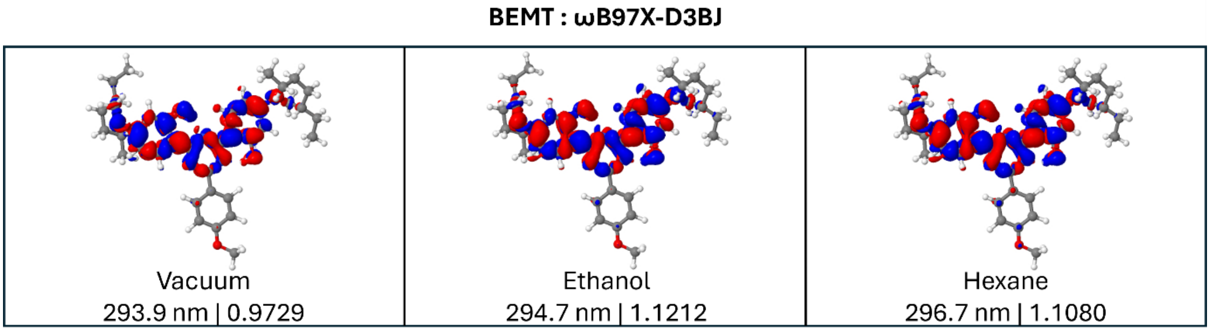


**Figure S21.** Transition densities for the $S_1 \leftarrow S_0$ transition calculated for the geometries of BEMT obtained through implicit solvent and vacuum relaxations in the ground state. Two implicit solvent environments were tested: hexane and ethanol. LR-TDDFT with the ωB97X-D3BJ functional, def2-TZVP basis set and CPCM implicit solvent model.

**Table S2.** Wavelengths (nm) and oscillator strengths (wavelength/oscillator strength) corresponding to the ten lowest energy singlet-singlet transitions for geometries of BEMT obtained through implicit solvent and vacuum relaxations in the ground state. Two implicit solvent environments were tested: hexane and ethanol. LR-TDDFT with the ωB97X-D3BJ functional, def2-TZVP basis set and CPCM implicit solvent model.

| Transition | Vacuum | Ethanol | Hexane |
|---|---|---|---|
| $S_1 \leftarrow S_0$ | 293.9/0.9729 | 294.7/1.1212 | 296.7/1.1080 |
| $S_2 \leftarrow S_0$ | 282.7/0.5362 | 284.7/0.7475 | 285.7/0.6768 |
| $S_3 \leftarrow S_0$ | 262.4/0.1254 | 263.7/0.2993 | 263.6/0.2689 |
| $S_4 \leftarrow S_0$ | 256.4/0.0544 | 256.7/0.0892 | 257.2/0.1646 |
| $S_5 \leftarrow S_0$ | 254.2/0.3252 | 253.1/0.0376 | 254.9/0.0633 |
| $S_6 \leftarrow S_0$ | 249.5/0.2100 | 250.3/0.2056 | 250.3/0.2137 |
| $S_7 \leftarrow S_0$ | 244.9/0.0108 | 246.6/0.0202 | 246.4/0.0201 |
| $S_8 \leftarrow S_0$ | 243.5/0.0097 | 244.3/0.0167 | 244.1/0.0154 |
| $S_9 \leftarrow S_0$ | 242.5/0.0045 | 242.2/0.0031 | 242.7/0.0038 |
| $S_{10} \leftarrow S_0$ | 236.6/0.0272 | 235.6/0.0015 | 236.2/0.02168 |

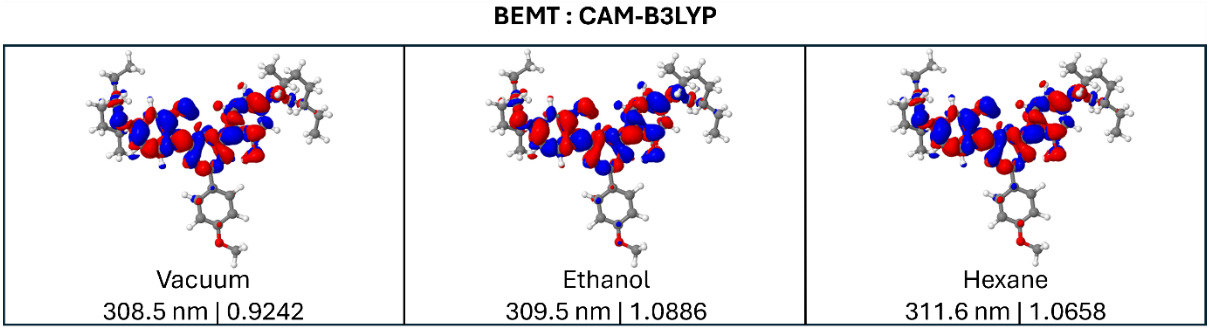


**Figure S22.** Transition densities for the $S_1 \leftarrow S_0$ transition calculated for the geometries of BEMT obtained through implicit solvent and vacuum relaxations in the ground state. Two implicit solvent environments were tested: hexane and ethanol. LR-TDDFT with the CAM-B3LYP functional, def2-TZVP basis set, D3BJ dispersion corrections, and CPCM implicit solvent model.

**Table S3**. Wavelengths (nm) and oscillator strengths (wavelength/oscillator strength) corresponding to the ten lowest energy singlet-singlet transitions for geometries of BEMT obtained through implicit solvent and vacuum relaxations in the ground state. Two implicit solvent environments were tested: hexane and ethanol. LR-TDDFT with the CAM-B3LYP functional, def2-TZVP basis set, D3BJ dispersion corrections, and CPCM implicit solvent model.

| Transition | Vacuum | Ethanol | Hexane |
|---|---|---|---|
| $S_1 \leftarrow S_0$ | 308.5/0.9242 | 309.5/1.0886 | 311.6/1.0658 |
| $S_2 \leftarrow S_0$ | 296.3/0.5192 | 298.8/0.7759 | 299.5/0.6773 |
| $S_3 \leftarrow S_0$ | 288.8/0.0111 | 285.8/0.0151 | 287.7/0.0130 |
| $S_4 \leftarrow S_0$ | 273.1/0.0755 | 274.2/0.3420 | 273.5/0.3216 |
| $S_5 \leftarrow S_0$ | 268.6/0.3723 | 269.8/0.0458 | 271.1/0.1625 |
| $S_6 \leftarrow S_0$ | 265.6/0.0935 | 263.0/0.0225 | 265.0/0.0268 |
| $S_7 \leftarrow S_0$ | 260.0/0.2528 | 260.5/0.2202 | 260.9/0.2449 |
| $S_8 \leftarrow S_0$ | 256.0/0.0110 | 258.2/0.0256 | 257.9/0.0273 |
| $S_9 \leftarrow S_0$ | 254.1/0.0013 | 256.1/0.0040 | 254.6/0.0026 |
| $S_{10} \leftarrow S_0$ | 252.0/0.0028 | 252.4/0.0027 | 252.7/0.0034 |

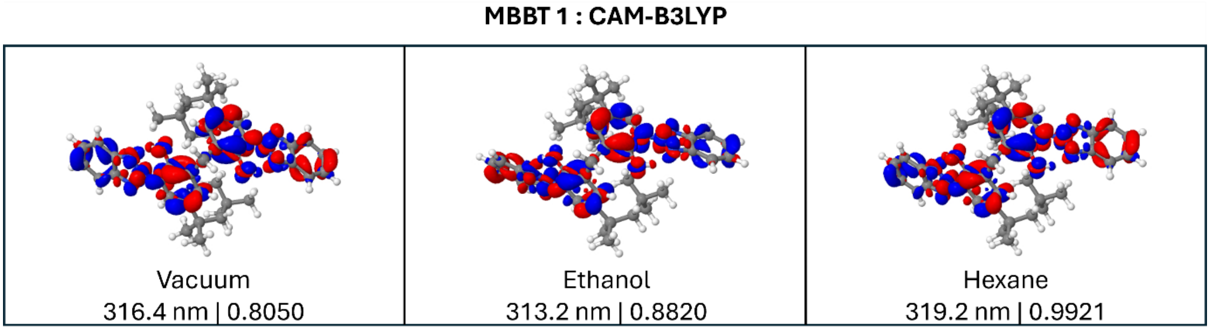


**Figure S23.** Transition densities for the $S_1 \leftarrow S_0$ transition calculated for the geometries of MBBT 1 obtained through implicit solvent and vacuum relaxations in the ground state. Two implicit solvent environments were tested: hexane and ethanol. LR-TDDFT with the ωB97X-D3BJ functional, def2-TZVP basis set and CPCM implicit solvent model.

**Table S4.** Wavelengths (nm) and oscillator strengths (wavelength/oscillator strength) corresponding to the ten lowest energy singlet-singlet transitions for geometries of MBBT 1 obtained through implicit solvent and vacuum relaxations in the ground state. Two implicit solvent environments were tested: hexane and ethanol. LR-TDDFT with the ωB97X-D3BJ functional, def2-TZVP basis set and CPCM implicit solvent model.

| Transition | Vacuum | Ethanol | Hexane |
|---|---|---|---|
| $S_1 \leftarrow S_0$ | 296.7/0.9563 | 297.8/1.1494 | 299.4/1.1512 |
| $S_2 \leftarrow S_0$ | 290.8/0.0728 | 292.6/0.1099 | 294.2/0.1066 |
| $S_3 \leftarrow S_0$ | 258.7/0.2605 | 259.5/0.1891 | 260.0/0.2496 |
| $S_4 \leftarrow S_0$ | 258.5/0.0031 | 258.9/0.01337 | 259.7/0.0052 |
| $S_5 \leftarrow S_0$ | 253.2/0.0320 | 256.5/0.02672 | 256.0/0.04308 |
| $S_6 \leftarrow S_0$ | 252.9/0.1533 | 256.2/0.3004 | 255.8/0.2470 |
| $S_7 \leftarrow S_0$ | 218.6/0.0687 | 218.6/0.1000 | 219.0/0.1001 |
| $S_8 \leftarrow S_0$ | 214.6/0.0015 | 215.0/0.0032 | 215.2/0.0027 |
| $S_9 \leftarrow S_0$ | 212.4/0.0016 | 211.9/0.0026 | 212.7/0.0030 |
| $S_{10} \leftarrow S_0$ | 210.7/0.0040 | 209.4/0.2531 | 210.4/0.0455 |

**MBBT 1 : CAM-B3LYP**

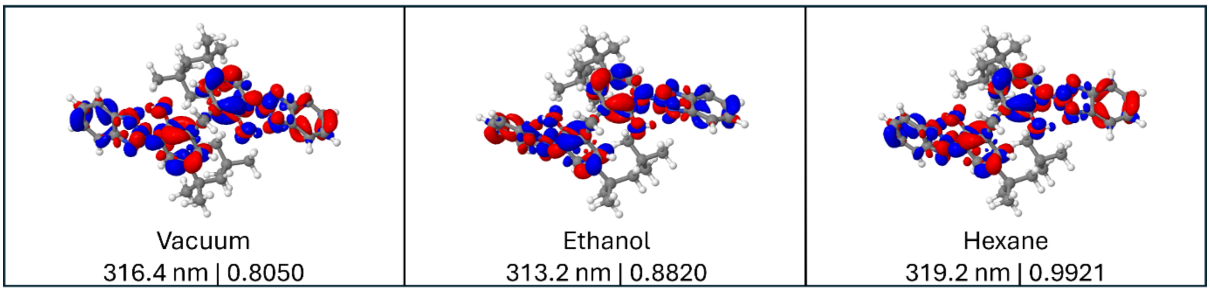


**Figure S24.** Transition densities for the $S_1 \leftarrow S_0$ transition calculated for the geometries of MBBT 1 obtained through implicit solvent and vacuum relaxations in the ground state. Two implicit solvent environments were tested: hexane and ethanol. LR-TDDFT with the CAM-B3LYP functional, def2-TZVP basis set, D3BJ dispersion corrections, and CPCM implicit solvent model.

**Table S5.** Wavelengths (nm) and oscillator strengths (wavelength/oscillator strength) corresponding to the ten lowest energy singlet-singlet transitions for geometries of MBBT 1 obtained through implicit solvent and vacuum relaxations in the ground state. Two implicit solvent environments were tested: hexane and ethanol. LR-TDDFT with the CAM-B3LYP functional, def2-TZVP basis set, D3BJ dispersion corrections, and CPCM implicit solvent model.

| Transition | Vacuum | Ethanol | Hexane |
|---|---|---|---|
| $S_1 \leftarrow S_0$ | 316.4/0.8050 | 313.2/0.8820 | 319.2/0.9921 |
| $S_2 \leftarrow S_0$ | 309.8/0.0708 | 307.9/0.1173 | 313.1/0.10428 |
| $S_3 \leftarrow S_0$ | 274.3/0.0098 | 273.1/0.0238 | 275.7/0.01068 |
| $S_4 \leftarrow S_0$ | 273.8/0.4028 | 272.2/0.3754 | 275.3/0.4179 |
| $S_5 \leftarrow S_0$ | 264.1/0.0345 | 267.8/0.09662 | 267.0/0.0501 |
| $S_6 \leftarrow S_0$ | 263.8/0.1166 | 267.8/0.1386 | 266.8/0.1783 |
| $S_7 \leftarrow S_0$ | 248.0/0.0000 | 244.5/0.0000 | 247.4/0.0000 |
| $S_8 \leftarrow S_0$ | 247.3/0.0356 | 244.2/0.0672 | 246.7/0.0416 |
| $S_9 \leftarrow S_0$ | 225.8/0.0191 | 225.6/0.1421 | 225.7/0.0322 |
| $S_{10} \leftarrow S_0$ | 224.0/0.1190 | 224.7/0.0650 | 224.3/0.1609 |

**MBBT 21 : ωB97X-D3BJ**

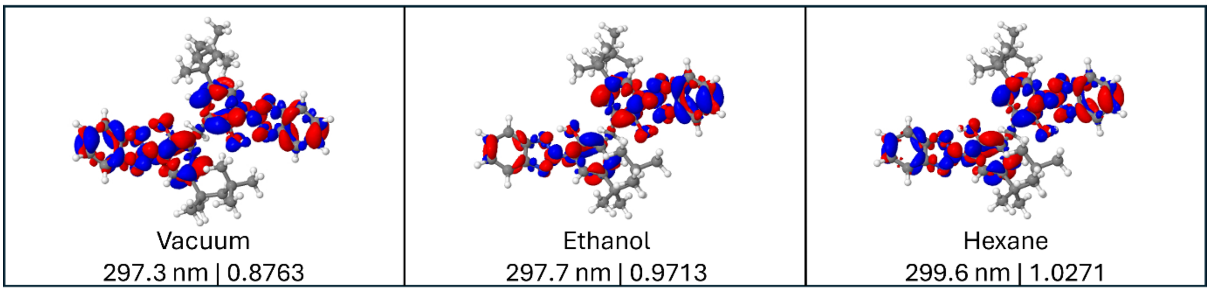


**Figure S25.** Transition densities for the $S_1 \leftarrow S_0$ transition calculated for the geometries of MBBT 21 obtained through implicit solvent and vacuum relaxations in the ground state. Two implicit solvent environments were tested: hexane and ethanol. LR-TDDFT with the ωB97X-D3BJ functional, def2-TZVP basis set and CPCM implicit solvent model.

**Table S6.** Wavelengths (nm) and oscillator strengths (wavelength/oscillator strength) corresponding to the ten lowest energy singlet-singlet transitions for geometries of MBBT 21 obtained through implicit solvent and vacuum relaxations in the ground state. Two implicit solvent environments were tested: hexane and ethanol. LR-TDDFT with the ωB97X-D3BJ functional, def2-TZVP basis set and CPCM implicit solvent model.

| Transition | Vacuum | Ethanol | Hexane |
| --- | --- | --- | --- |
| $S_1 \leftarrow S_0$ | 297.3/0.8763 | 297.7/0.9713 | 299.6/1.0271 |
| $S_2 \leftarrow S_0$ | 290.5/0.1607 | 291.2/0.2763 | 293.6/0.2267 |
| $S_3 \leftarrow S_0$ | 258.0/0.2420 | 258.8/0.1500 | 259.2/0.2258 |
| $S_4 \leftarrow S_0$ | 256.5/0.0244 | 257.2/0.0490 | 257.4/0.0365 |
| $S_5 \leftarrow S_0$ | 253.0/0.0658 | 256.0/0.2547 | 255.8/0.1040 |
| $S_6 \leftarrow S_0$ | 252.4/0.1354 | 255.4/0.1000 | 255.3/0.2031 |
| $S_7 \leftarrow S_0$ | 217.1/0.0392 | 217.3/0.0651 | 217.6/0.0625 |
| $S_8 \leftarrow S_0$ | 214.1/0.0007 | 214.6/0.0005 | 214.7/0.0006 |
| $S_9 \leftarrow S_0$ | 211.8/0.0037 | 211.0/0.0101 | 212.0/0.0072 |
| $S_{10} \leftarrow S_0$ | 210.5/0.0040 | 209.2/0.3098 | 210.2/0.0487 |

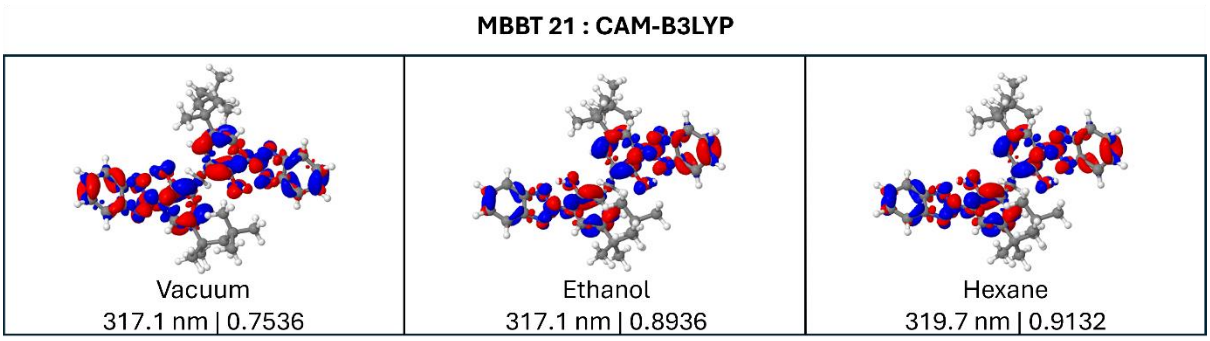


**Figure S26.** Transition densities for the $S_1 \leftarrow S_0$ transition calculated for the geometries of MBBT 21 obtained through implicit solvent and vacuum relaxations in the ground state. Two implicit solvent environments were tested: hexane and ethanol. LR-TDDFT with the CAM-B3LYP functional, def2-TZVP basis set, D3BJ dispersion corrections, and CPCM implicit solvent model.

**Table S7.** Wavelengths (nm) and oscillator strengths (wavelength/oscillator strength) corresponding to the ten lowest energy singlet-singlet transitions for geometries of MBBT 21 obtained through implicit solvent and vacuum relaxations in the ground state. Two implicit solvent environments were tested: hexane and ethanol. LR-TDDFT with the CAM-B3LYP functional, def2-TZVP basis set, D3BJ dispersion corrections, and CPCM implicit solvent model.

| Transition | Vacuum | Ethanol | Hexane |
|---|---|---|---|
| $S_1 \leftarrow S_0$ | 317.1/0.7536 | 317.1/0.8936 | 319.7/0.9132 |
| $S_2 \leftarrow S_0$ | 309.0/0.1430 | 309.8/0.2176 | 312.5/0.1983 |
| $S_3 \leftarrow S_0$ | 272.3/0.3756 | 272.6/0.3118 | 273.8/0.3916 |
| $S_4 \leftarrow S_0$ | 271.3/0.0483 | 271.1/0.0709 | 272.6/0.0485 |
| $S_5 \leftarrow S_0$ | 263.8/.0577 | 267.7/0.0731 | 266.6/0.0777 |
| $S_6 \leftarrow S_0$ | 263.0/0.1153 | 267.1/0.2225 | 266.0/0.1787 |
| $S_7 \leftarrow S_0$ | 249.0/0.0041 | 245.4/0.0017 | 247.9/0.0036 |
| $S_8 \leftarrow S_0$ | 244.9/0.0076 | 243.0/0.0093 | 244.5/0.0092 |
| $S_9 \leftarrow S_0$ | 224.4/0.0252 | 223.7/0.1439 | 224.2/0.0885 |
| $S_{10} \leftarrow S_0$ | 223.8/0.0868 | 222.9/0.0235 | 223.8/0.0729 |

### 2.3.2. Gas phase calculations

#### 2.3.2.1. Vertical excitations and transition densities

**Table S8.** Wavelengths, energies and oscillator strengths corresponding to the ten lowest energy singlet-singlet transitions for the Franck-Condon (FC) geometry of BEMT. These were calculated in vacuum using LR-TDDFT with the CAM-B3LYP functional, def2-TZVP basis set, and D3BJ dispersion corrections.

| Transition | Excitation Wavelength (nm) | Excitation Energy (eV) | Oscillator Strength |
|---|---|---|---|
| $S_1 \leftarrow S_0$ | 308.5 | 4.018683 | 0.924150975 |
| $S_2 \leftarrow S_0$ | 296.3 | 4.184866 | 0.519246816 |
| $S_3 \leftarrow S_0$ | 288.8 | 4.293530 | 0.011109444 |
| $S_4 \leftarrow S_0$ | 273.1 | 4.539115 | 0.075487349 |
| $S_5 \leftarrow S_0$ | 268.6 | 4.616344 | 0.372305783 |
| $S_6 \leftarrow S_0$ | 265.6 | 4.667730 | 0.093454011 |
| $S_7 \leftarrow S_0$ | 260.0 | 4.767903 | 0.252784849 |
| $S_8 \leftarrow S_0$ | 256.0 | 4.843436 | 0.011004049 |
| $S_9 \leftarrow S_0$ | 254.1 | 4.879891 | 0.001320000 |
| $S_{10} \leftarrow S_0$ | 252.0 | 4.919195 | 0.002817063 |

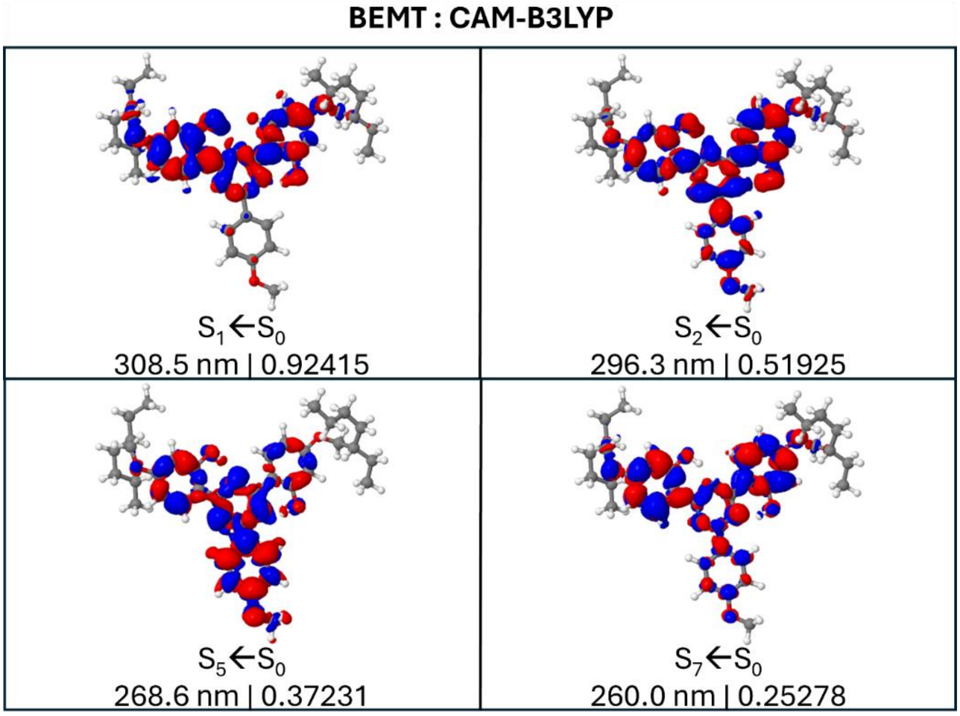


**Figure S27.** Transition densities corresponding to all the non-negligible transitions (oscillator strength > 0.1) in the ten lowest energy singlet-singlet transitions for the FC geometry of BEMT, as predicted by LR-TDDFT with the CAM-B3LYP functional, def2-TZVP basis set, and D3BJ dispersion corrections. Below the transition densities are the corresponding absorption wavelengths and oscillator strengths.

**Table S9.** Wavelengths, energies and oscillator strengths corresponding to the ten lowest energy singlet-singlet transitions for a ground state relaxed geometry of BEMT. These were calculated in vacuum using LR-TDDFT with the ωB97X-D3BJ functional and def2-TZVP basis set.

| Transition | Excitation Wavelength (nm) | Excitation Energy (eV) | Oscillator Strength |
|---|---|---|---|
| $S_1 \leftarrow S_0$ | 293.9 | 4.218214 | 0.972892514 |
| $S_2 \leftarrow S_0$ | 282.7 | 4.386248 | 0.536182585 |
| $S_3 \leftarrow S_0$ | 262.4 | 4.724395 | 0.125412985 |
| $S_4 \leftarrow S_0$ | 256.4 | 4.836093 | 0.054436978 |
| $S_5 \leftarrow S_0$ | 254.2 | 4.877410 | 0.325172425 |
| $S_6 \leftarrow S_0$ | 249.5 | 4.968503 | 0.209974047 |
| $S_7 \leftarrow S_0$ | 244.9 | 5.061743 | 0.010758227 |
| $S_8 \leftarrow S_0$ | 243.5 | 5.091270 | 0.009676955 |
| $S_9 \leftarrow S_0$ | 242.5 | 5.112102 | 0.004463330 |
| $S_{10} \leftarrow S_0$ | 236.6 | 5.239703 | 0.027219099 |

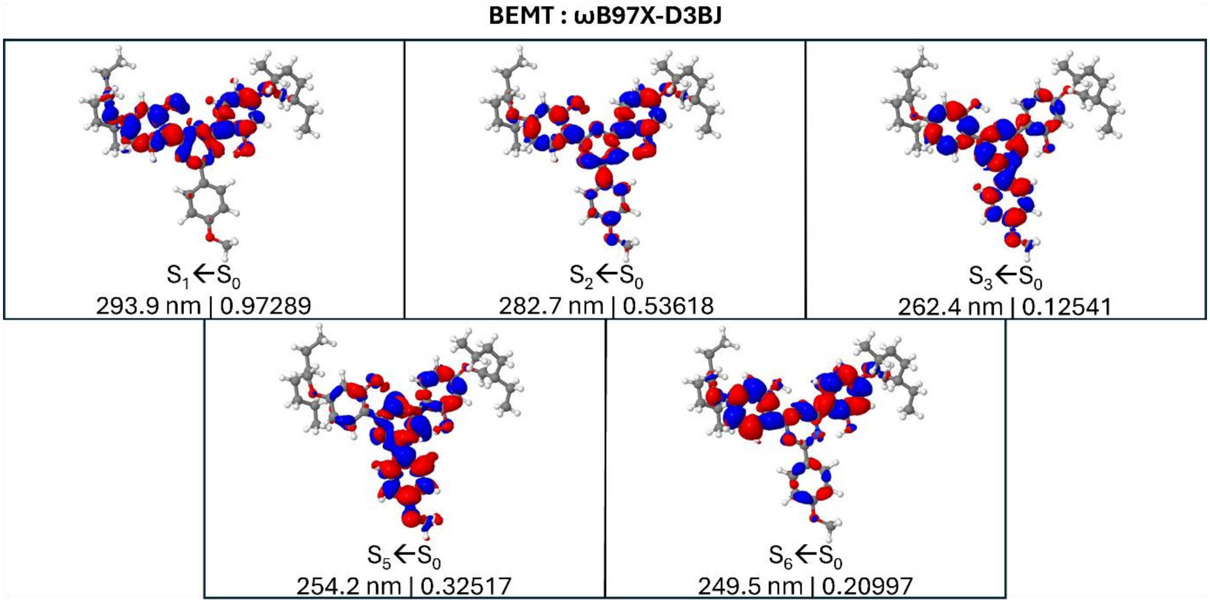


**Figure S28.** Transition densities corresponding to all the non-negligible transitions (oscillator strength > 0.1) in the ten lowest energy singlet-singlet transitions for the FC geometry of BEMT, as predicted by LR-TDDFT with the ωB97X-D3BJ functional, and def2-TZVP basis set. Below the transition densities are the corresponding absorption wavelengths and oscillator strengths.

**Table S10.** Wavelengths, energies and oscillator strengths corresponding to the ten lowest energy singlet-singlet transitions for a ground state relaxed geometry of BEMT. These were calculated in vacuum using the B3LYP functional, def2-TZVP basis set, and D3BJ dispersion correction.

| Transition | Excitation Wavelength (nm) | Excitation Energy (eV) | Oscillator Strength |
|---|---|---|---|
| $S_1 \leftarrow S_0$ | 366.9 | 3.379059 | 0.023989243 |
| $S_2 \leftarrow S_0$ | 357.8 | 3.465560 | 0.040861379 |
| $S_3 \leftarrow S_0$ | 347.5 | 3.567421 | 0.656504329 |
| $S_4 \leftarrow S_0$ | 333.8 | 3.714701 | 0.346539697 |
| $S_5 \leftarrow S_0$ | 312.9 | 3.962413 | 0.048636886 |
| $S_6 \leftarrow S_0$ | 309.3 | 4.008661 | 0.269012212 |
| $S_7 \leftarrow S_0$ | 306.4 | 4.046541 | 0.268311393 |
| $S_8 \leftarrow S_0$ | 304.1 | 4.076797 | 0.122402464 |
| $S_9 \leftarrow S_0$ | 299.5 | 4.139881 | 0.135694596 |
| $S_{10} \leftarrow S_0$ | 295.4 | 4.196621 | 0.011767446 |

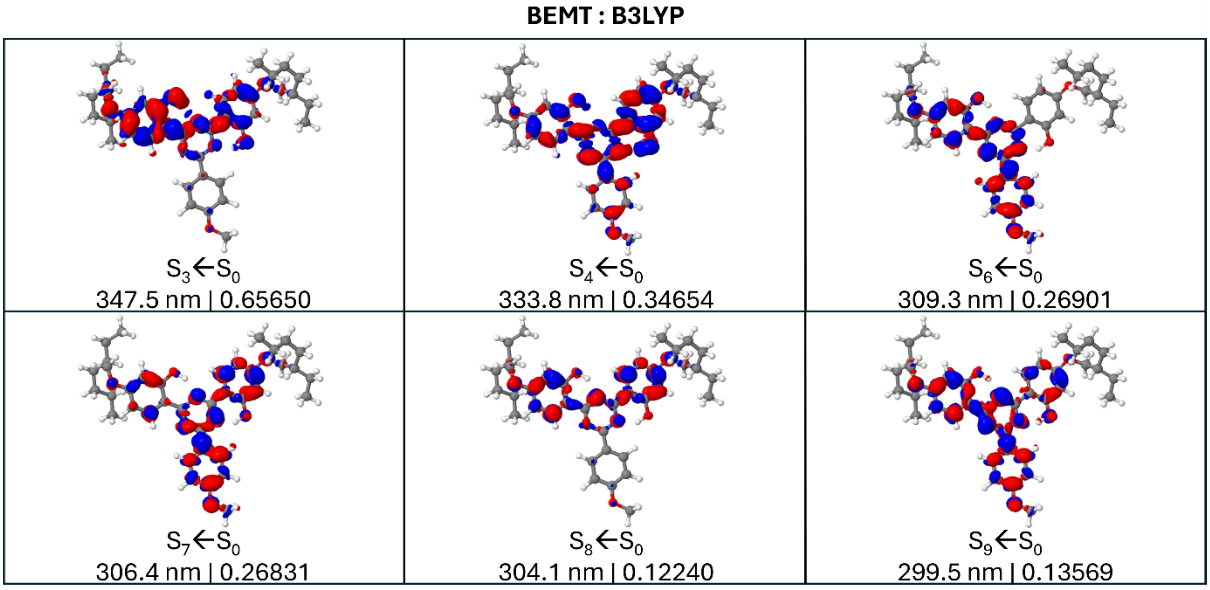


**Figure S29.** Transition densities corresponding to all the non-negligible transitions (oscillator strength > 0.1) in the ten lowest energy singlet-singlet transitions for the FC geometry of BEMT, as predicted by LR-TDDFT with the B3LYP functional, def2-TZVP basis set, and D3BJ dispersion corrections. Below the transition densities are the corresponding absorption wavelengths and oscillator strengths.

**Table S11.** Wavelengths, energies and oscillator strengths corresponding to the ten lowest energy singlet-singlet transitions for the ground state relaxed geometry of MBBT 1. These were calculated in vacuum using the CAM-B3LYP functional, def2-TZVP basis set, and D3BJ dispersion corrections.

| Transition | Excitation Wavelength (nm) | Excitation Energy (eV) | Oscillator Strength |
|---|---|---|---|
| $S_1 \leftarrow S_0$ | 316.4 | 3.918138 | 0.805018974 |
| $S_2 \leftarrow S_0$ | 309.8 | 4.002072 | 0.070777519 |
| $S_3 \leftarrow S_0$ | 274.3 | 4.520492 | 0.009780858 |
| $S_4 \leftarrow S_0$ | 273.8 | 4.528808 | 0.402771631 |
| $S_5 \leftarrow S_0$ | 264.1 | 4.695324 | 0.034460960 |
| $S_6 \leftarrow S_0$ | 263.8 | 4.699152 | 0.116555477 |
| $S_7 \leftarrow S_0$ | 248.0 | 4.999643 | 0.000003288 |
| $S_8 \leftarrow S_0$ | 247.3 | 5.014269 | 0.035584592 |
| $S_9 \leftarrow S_0$ | 225.8 | 5.491683 | 0.019124983 |
| $S_{10} \leftarrow S_0$ | 224.0 | 5.535597 | 0.118963267 |

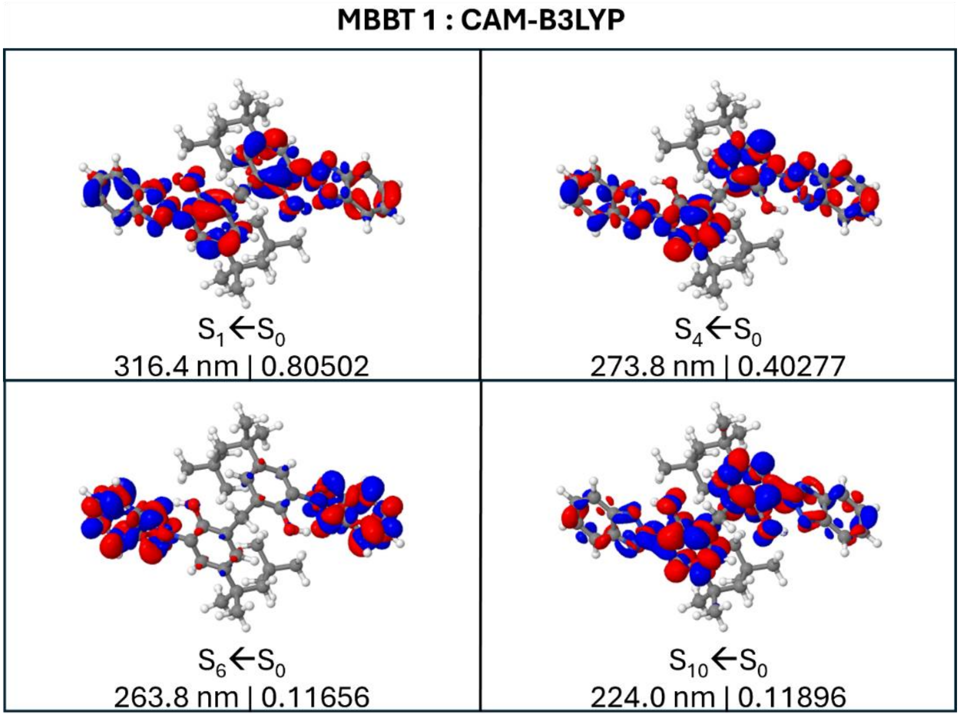


**Figure S30.** Transition densities corresponding to all the non-negligible transitions (oscillator strength > 0.1) in the ten lowest energy singlet-singlet transitions for the FC geometry of MBBT 1, as predicted by LR-TDDFT with the CAM-B3LYP functional, def2-TZVP basis set, and D3BJ dispersion corrections. Below the transition densities are the corresponding absorption wavelengths and oscillator strengths.

**Table S12.** Wavelengths, energies and oscillator strengths corresponding to the ten lowest energy singlet-singlet transitions for the ground state relaxed geometry of MBBT 1. These were calculated in vacuum using the ωB97X-D3BJ functional and def2-TZVP basis set.

| Transition | Excitation Wavelength (nm) | Excitation Energy (eV) | Oscillator Strength |
|---|---|---|---|
| $S_1 \leftarrow S_0$ | 296.7 | 4.179379 | 0.956308102 |
| $S_2 \leftarrow S_0$ | 290.8 | 4.263979 | 0.072816234 |
| $S_3 \leftarrow S_0$ | 258.7 | 4.793006 | 0.260531821 |
| $S_4 \leftarrow S_0$ | 258.5 | 4.795440 | 0.003096203 |
| $S_5 \leftarrow S_0$ | 253.2 | 4.897542 | 0.031967241 |
| $S_6 \leftarrow S_0$ | 252.9 | 4.902317 | 0.153331912 |
| $S_7 \leftarrow S_0$ | 218.6 | 5.671913 | 0.068681838 |
| $S_8 \leftarrow S_0$ | 214.6 | 5.776550 | 0.001455985 |
| $S_9 \leftarrow S_0$ | 212.4 | 5.836250 | 0.001691298 |
| $S_{10} \leftarrow S_0$ | 210.7 | 5.883702 | 0.004038737 |

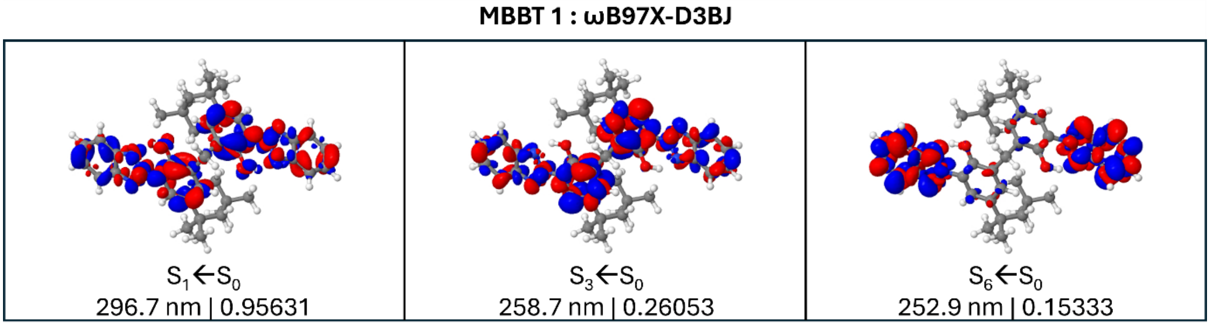


**Figure S31.** Transition densities corresponding to all the non-negligible transitions (oscillator strength > 0.1) in the ten lowest energy singlet-singlet transitions for the FC geometry of MBBT 1, as predicted by LR-TDDFT with the ωB97X-D3BJ functional and def2-TZVP basis set. Below the transition densities are the corresponding absorption wavelengths and oscillator strengths.

**Table S13.** Wavelengths, energies and oscillator strengths corresponding to the ten lowest energy singlet-singlet transitions for the ground state relaxed geometry of MBBT 1. These were calculated in vacuum using the B3LYP functional, def2-TZVP basis set, and D3BJ dispersion correction.

| Transition | Excitation Wavelength (nm) | Excitation Energy (eV) | Oscillator Strength |
|---|---|---|---|
| $S_1 \leftarrow S_0$ | 377.1 | 3.287566 | 0.311698070 |
| $S_2 \leftarrow S_0$ | 372.5 | 3.328873 | 0.029540025 |
| $S_3 \leftarrow S_0$ | 359.4 | 3.449423 | 0.103813926 |
| $S_4 \leftarrow S_0$ | 357.5 | 3.468368 | 0.016087763 |
| $S_5 \leftarrow S_0$ | 321.7 | 3.853707 | 0.030691192 |
| $S_6 \leftarrow S_0$ | 319.1 | 3.885987 | 0.465836633 |
| $S_7 \leftarrow S_0$ | 302.6 | 4.097030 | 0.224616523 |
| $S_8 \leftarrow S_0$ | 300.1 | 4.131818 | 0.018485592 |
| $S_9 \leftarrow S_0$ | 296.1 | 4.186663 | 0.019572691 |
| $S_{10} \leftarrow S_0$ | 296.0 | 4.189323 | 0.089622052 |

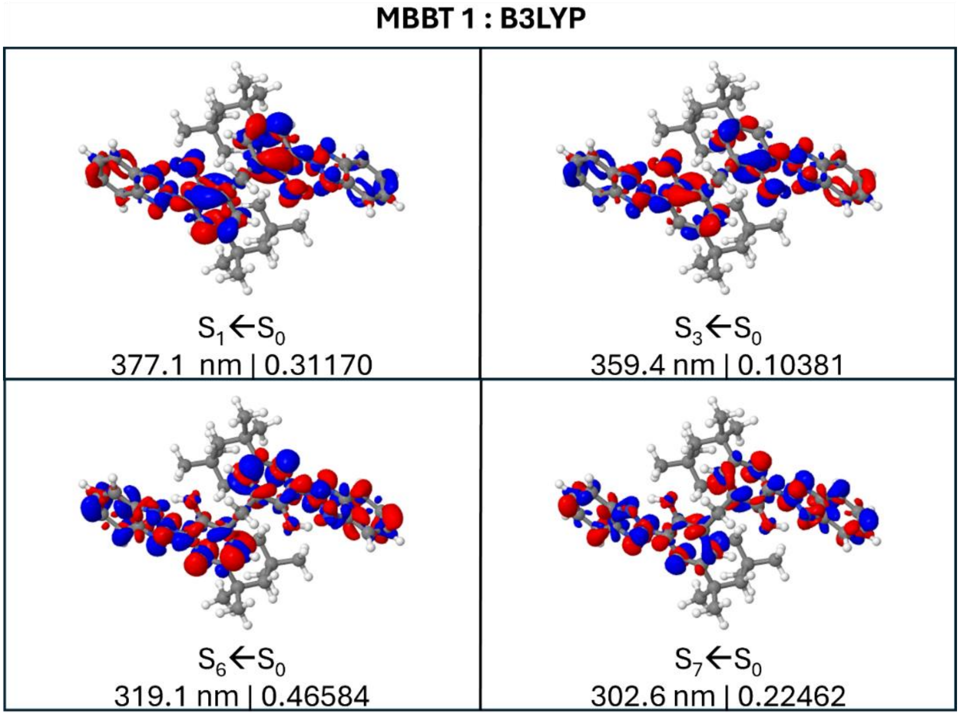


**Figure S32.** Transition densities corresponding to all the non-negligible transitions (oscillator strength > 0.1) in the ten lowest energy singlet-singlet transitions for the FC geometry of MBBT 1, as predicted by LR-TDDFT with the B3LYP functional, def2-TZVP basis set, and D3BJ dispersion corrections. Below the transition densities are the corresponding absorption wavelengths and oscillator strengths.

**Table S14.** Wavelengths, energies and oscillator strengths corresponding to the ten lowest energy singlet-singlet transitions for the ground state relaxed geometry of MBBT 21. These were calculated in vacuum using the CAM-B3LYP functional, def2-TZVP basis set and D3BJ dispersion corrections.

| Transition | Excitation Wavelength (nm) | Excitation Energy (eV) | Oscillator Strength |
|---|---|---|---|
| $S_1 \leftarrow S_0$ | 317.1 | 3.910478 | 0.753622876 |
| $S_2 \leftarrow S_0$ | 309.0 | 4.012688 | 0.142979713 |
| $S_3 \leftarrow S_0$ | 272.3 | 4.553895 | 0.375645372 |
| $S_4 \leftarrow S_0$ | 271.3 | 4.570232 | 0.048250141 |
| $S_5 \leftarrow S_0$ | 263.8 | 4.700382 | 0.057673154 |
| $S_6 \leftarrow S_0$ | 263.0 | 4.713527 | 0.115320502 |
| $S_7 \leftarrow S_0$ | 249.0 | 4.979849 | 0.004113091 |
| $S_8 \leftarrow S_0$ | 244.9 | 5.061770 | 0.007624173 |
| $S_9 \leftarrow S_0$ | 224.4 | 5.525268 | 0.025233995 |
| $S_{10} \leftarrow S_0$ | 223.8 | 5.540650 | 0.086771874 |

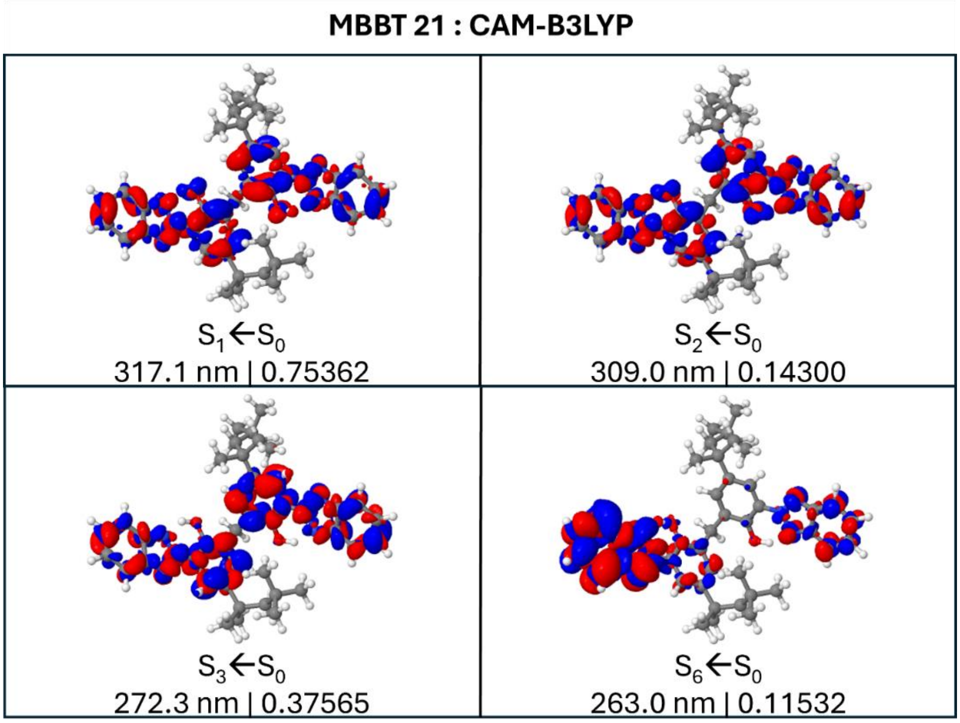


**Figure S33.** Transition densities corresponding to all the non-negligible transitions (oscillator strength > 0.1) in the ten lowest energy singlet-singlet transitions for the FC geometry of MBBT 21, as predicted by LR-TDDFT with the CAM-B3LYP functional, def2-TZVP basis set and D3BJ dispersion corrections. Below the transition densities are the corresponding absorption wavelengths and oscillator strengths.

**Table S15.** Wavelengths, energies and oscillator strengths corresponding to the ten lowest energy singlet-singlet transitions for the ground state relaxed geometry of MBBT 21. These were calculated in vacuum using the ωB97X-D3BJ functional and def2-TZVP basis set.

| Transition | Excitation Wavelength (nm) | Excitation Energy (eV) | Oscillator Strength |
|---|---|---|---|
| $S_1 \leftarrow S_0$ | 297.3 | 4.169938 | 0.876309209 |
| $S_2 \leftarrow S_0$ | 290.5 | 4.267553 | 0.160685640 |
| $S_3 \leftarrow S_0$ | 258.0 | 4.805990 | 0.241974856 |
| $S_4 \leftarrow S_0$ | 256.5 | 4.833717 | 0.024422889 |
| $S_5 \leftarrow S_0$ | 253.0 | 4.900288 | 0.065758297 |
| $S_6 \leftarrow S_0$ | 252.4 | 4.911624 | 0.135391562 |
| $S_7 \leftarrow S_0$ | 217.1 | 5.710424 | 0.039216772 |
| $S_8 \leftarrow S_0$ | 214.1 | 5.790182 | 0.000721453 |
| $S_9 \leftarrow S_0$ | 211.8 | 5.854189 | 0.003735203 |
| $S_{10} \leftarrow S_0$ | 210.5 | 5.889122 | 0.004009237 |

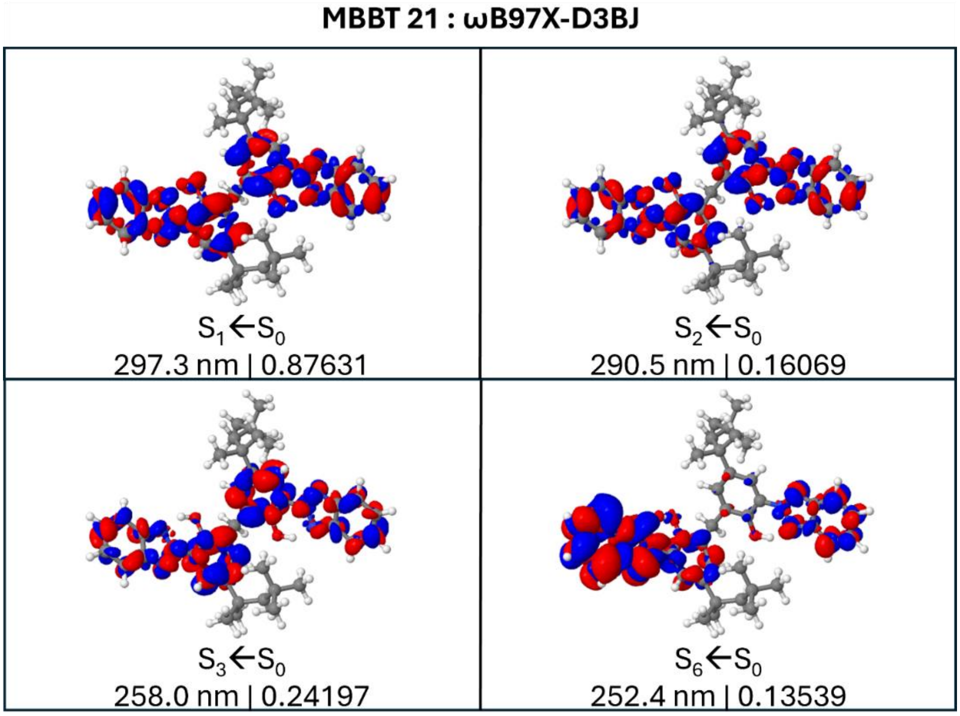


**Figure S34.** Transition densities corresponding to all the non-negligible transitions (oscillator strength > 0.1) in the ten lowest energy singlet-singlet transitions for the FC geometry of MBBT 21, as predicted by LR-TDDFT with the ωB97X-D3BJ functional and def2-TZVP basis set. Below the transition densities are the corresponding absorption wavelengths and oscillator strengths.

**Table S16.** Wavelengths, energies and oscillator strengths corresponding to the ten lowest energy singlet-singlet transitions for the ground state relaxed geometry of MBBT 21. These were calculated in vacuum using the B3LYP functional, def2-TZVP basis set and D3BJ dispersion correction.

| Transition | Excitation Wavelength (nm) | Excitation Energy (eV) | Oscillator Strength |
|---|---|---|---|
| $S_1 \leftarrow S_0$ | 375.2 | 3.304075 | 0.379276431 |
| $S_2 \leftarrow S_0$ | 372.7 | 3.327023 | 0.021371020 |
| $S_3 \leftarrow S_0$ | 363.9 | 3.406672 | 0.066862984 |
| $S_4 \leftarrow S_0$ | 355.3 | 3.489489 | 0.025982710 |
| $S_5 \leftarrow S_0$ | 323.5 | 3.832009 | 0.049516583 |
| $S_6 \leftarrow S_0$ | 319.4 | 3.881632 | 0.486418427 |
| $S_7 \leftarrow S_0$ | 302.6 | 4.097509 | 0.215528368 |
| $S_8 \leftarrow S_0$ | 297.8 | 4.163268 | 0.037043797 |
| $S_9 \leftarrow S_0$ | 296.2 | 4.185732 | 0.051173800 |
| $S_{10} \leftarrow S_0$ | 295.3 | 4.198201 | 0.060920549 |

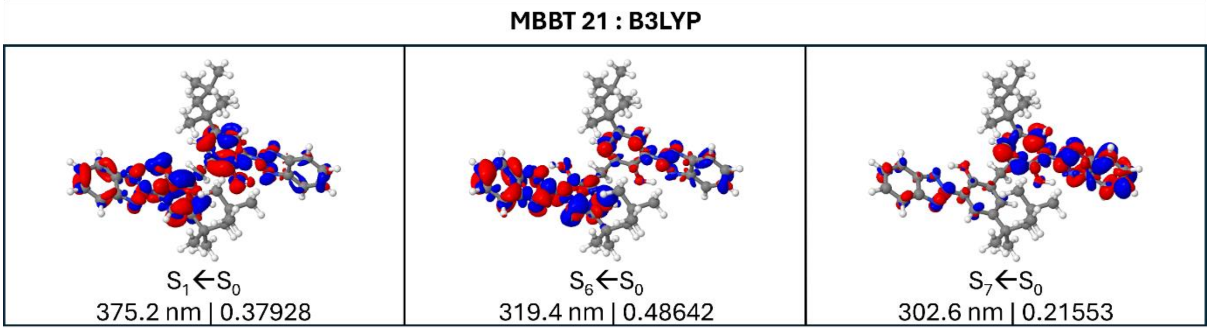


**Figure S35.** Transition densities corresponding to all the non-negligible transitions (oscillator strength > 0.1) in the ten lowest energy singlet-singlet transitions for the FC geometry of MBBT 21, as predicted by LR-TDDFT with the B3LYP functional, def2-TZVP basis set and D3BJ dispersion corrections. Below the transition densities are the corresponding absorption wavelengths and oscillator strengths.

### 2.3.2.2. Discussion of gas phase calculations and chosen exchange-correlation functionals

For BEMT, the lowest energy peak is assigned to the $S_1 \leftarrow S_0$ and $S_2 \leftarrow S_0$ transitions, with the excitation to the first excited state being the dominant transition for the molecule (see Tables S8-10). The higher energy peak is dominated by the $S_5 \leftarrow S_0$ and $S_6 \leftarrow S_0$ (ωB97X)/$S_7 \leftarrow S_0$ (CAM-B3LYP) transitions. $S_6$ and $S_7$ are near-degenerate in the calculations with both functionals and the transition densities associated with $S_6 \leftarrow S_0$ (ωB97X) and $S_7 \leftarrow S_0$ (CAM-B3LYP) are very similar, suggesting this slight difference in assignment results solely from a different energetic ordering of states, rather than the occupation of a state with significantly different electronic character. The hybrid B3LYP functional shows poor agreement with the range-separated functionals, identifying significant bright transitions from the ground state to the $S_3$, $S_4$, $S_6$, and $S_7$ states. Notably, the transition densities associated with the $S_1 \leftarrow S_0$ transition, as calculated by ωB97X and CAM-B3LYP, bear great similarity to the $S_3 \leftarrow S_0$ transition as calculated with B3LYP, indicating that a similar redistribution of charge occurs upon excitation to each of these theoretical adiabatic states (see Figures S27-29).

For MBBT, ωB97X and CAM-B3LYP calculations indicate that the lowest energy experimental peak corresponds almost purely to the $S_1 \leftarrow S_0$ transition, which has the highest calculated oscillator strength of all transitions in MBBT (see Tables S11-16). The higher energy peak is predominantly assigned to the $S_3 \leftarrow S_0$, with weak yet consistent contributions from the $S_6 \leftarrow S_0$ transition. Conversely, B3LYP calculations identify the $S_6 \leftarrow S_0$ as the transition with the strongest oscillator strength and find negligible contributions from the occupation of the $S_3$ state, instead suggesting that the $S_7 \leftarrow S_0$ transition contributes non-negligibly to the higher energy peak. Of the transition densities obtained using ωB97X and CAM-B3LYP, the $S_6 \leftarrow S_0$ are predominantly localised on the benzotriazole moieties, and the $S_3 \leftarrow S_0$ are spread over the benzotriazole and phenolic groups. It is noteworthy that the $S_6 \leftarrow S_0$ and $S_7 \leftarrow S_0$ transition densities calculated with B3LYP also extend across the benzotriazole and phenolic moieties; despite the functionals assigning different dominant transitions, the predicted movements of charge upon excitation are very similar, suggesting that all corresponding excited states have similar electronic structure (see Figures S30-35).

Despite B3LYP identifying sets of bright transitions with wavelengths closer to the experimentally observed absorption spectra peaks than the CAM-B3LYP and ωB97X functional, we do not consider it to be the optimal functional for studying these systems. While the range-separated hybrid functionals both over-estimate the $S_1 \leftarrow S_0$ vertical excitation energies, they do reproduce the spacing between the experimental peaks with similar accuracy to B3LYP. Hence, while there appears to be a systematic overestimation of $S_n \leftarrow S_0$ excitation energies with CAM-B3LYP and ωB97X, this does not raise concern regarding the accuracy of each state's calculated electronic structure. In isolation, successfully

reproducing experimental wavelengths at calculated FC geometries does not ensure that B3LYP will accurately calculate properties of the first excited state away from these relaxed geometries. In fact, the disagreement of B3LYP with both CAM-B3LYP and ωB97X suggests that the accuracy with which B3LYP calculates the electronic structure of MBBT and BEMT is potentially limited by its global treatment of exchange energy. The close agreement in assigned states and corresponding transition densities between two range-separated hybrid functionals—which belong to different 'families' of functionals—imparts confidence that both functionals permit the accurate calculation of the electronic structures of MBBT and BEMT. While two different functionals calculating similar properties for systems does not necessarily confirm the accuracy of the properties, a consistent agreement across relevant regions of phase space would certainly add credence to the results. Hence, in the following section, we deviate from the well-established norm of studying the excited state behaviours of these molecules using B3LYP.[8, 32-35] Instead, we use both CAM-B3LYP and ωB97X, with any significant deviation in calculated trends across phase space indicating potential inaccuracy.

## 2.4. Radial distribution functions

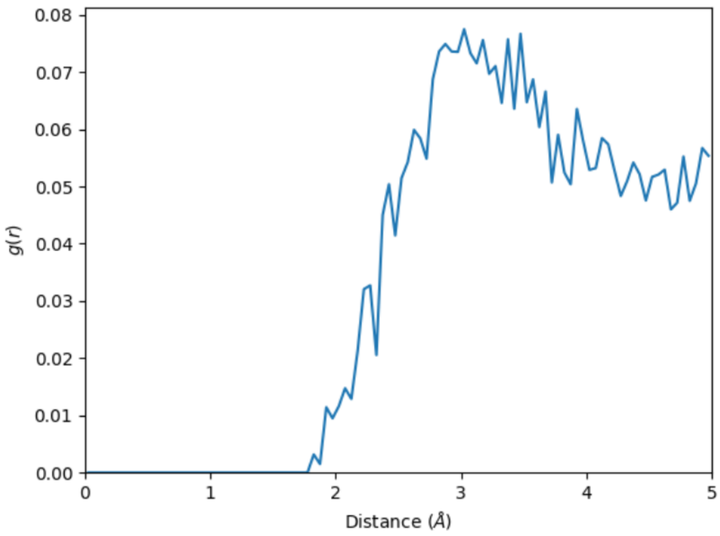


**Figure S36**. Radial distribution function of ethanolic -OH hydrogens around the proton acceptor nitrogen atom of MBBT 21 over 2500 snapshots of explicitly solvated molecular dynamics at 300 K.

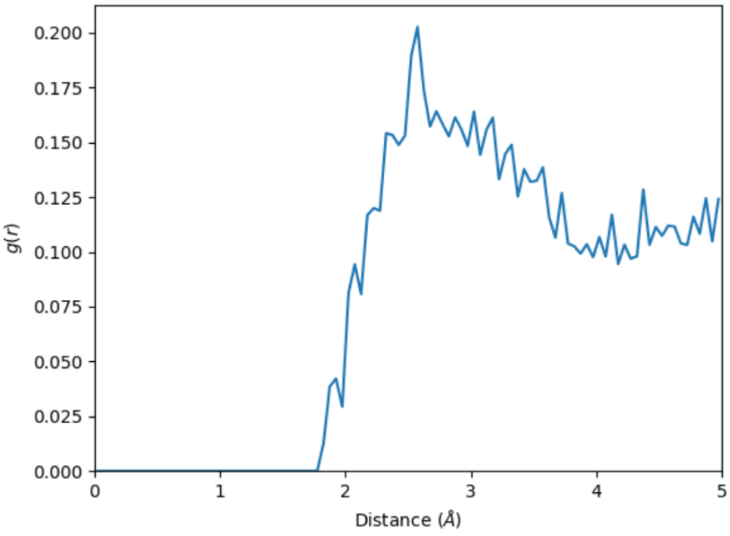


**Figure S37**. Radial distribution function of ethanolic -OH hydrogens around the proton acceptor nitrogen atom of Tinuvin P over 2500 snapshots of explicitly solvated molecular dynamics at 300 K.

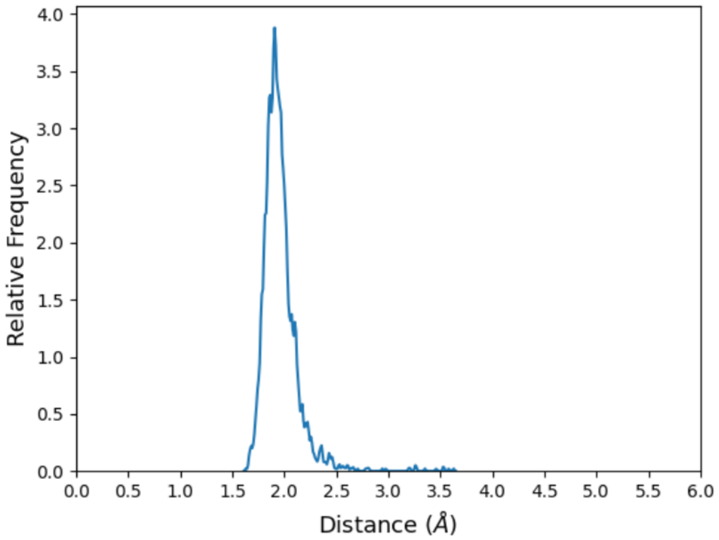


**Figure S38**.Kernel density estimate of the distances between the acceptor nitrogen and the phenolic hydrogen within MBBT 21 over 2500 snapshots of explicitly solvated (ethanol) molecular dynamics at 300 K. The peak around 1.9 Å corresponds to the acceptor hydrogen pointing at the donor nitrogen.

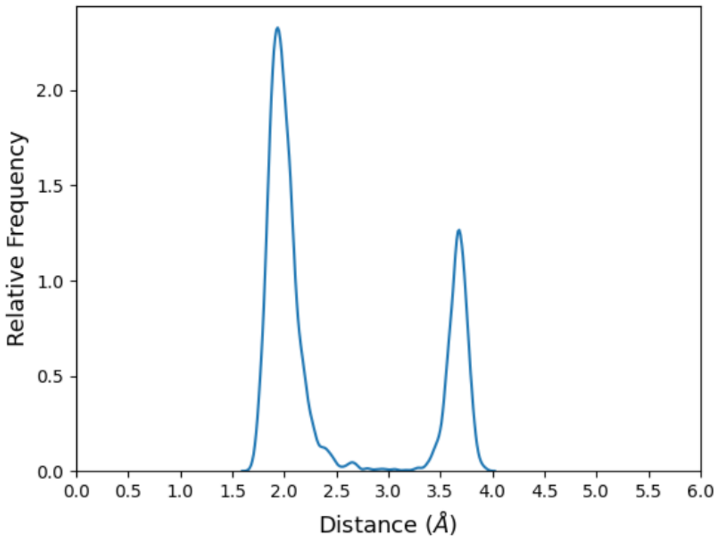


**Figure S39**. Kernel density estimate of the distances between the acceptor nitrogen and the phenolic hydrogen within Tinuvin P over 2500 snapshots of explicitly solvated (ethanol) molecular dynamics at 300 K. The peak around 1.9 Å corresponds to the acceptor hydrogen pointing at the donor nitrogen, and the peak around 3.6 Å results from the acceptor hydrogen rotating away from the donor nitrogen.

### 2.5. Elucidation of relaxation mechanisms

#### 2.5.1. Generation of trajectories

##### 2.5.1.1. BEMT:

When relaxing the FC geometry using CAM-B3LYP, ESPT was observed. Hence, to generate the CAM-B3LYP ESPT trajectory, we carried out a 20-step linear interpolation of internal coordinates between the FC geometry and this $S_1$ relaxed geometry. No such ESPT was observed when relaxing the ωB97X FC geometry in the $S_1$ state. Therefore, a post-ESPT geometry was generated, using the FC geometry as a starting point. This was achieved by transplanting the proton to the accepting nitrogen, fixing the resulting CNH angle to match the COH angle (α in Figure S40) in the FC geometry, and fixing the N-H bond length to 0.95 Å. The resulting geometry then underwent a constrained ωB97X LR-TDDFT relaxation in $S_1$ in the gas phase, with the $\phi_S$ (Figure 3 of the main text) being fixed to the value in the FC geometry. A linear interpolation was performed between the ωB97X FC geometry and the resulting post-ESPT geometry to obtain a 20-step trajectory. Iterative rotations of the $\phi_S$ dihedral angles were then applied to the post-ESPT geometries, moving with steps of 10° from the post-ESPT geometries (same $\phi_S$ as the FC geometries) to the $\phi_S$ angles observed in the relevant CI geometries (Table S17).

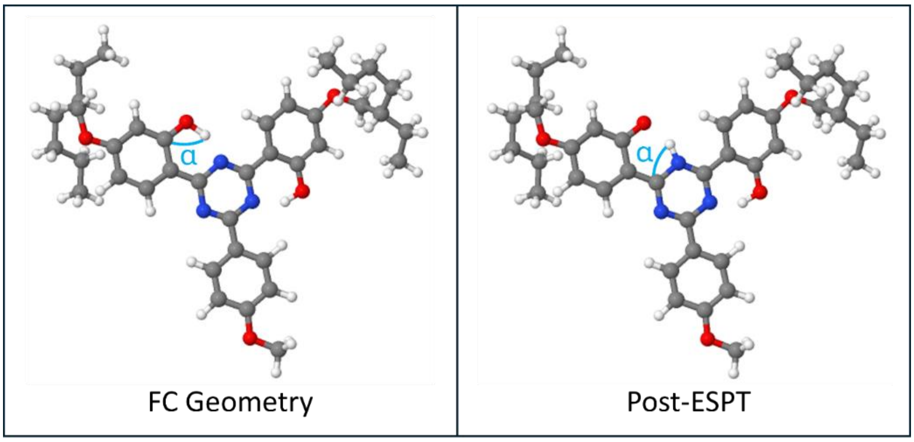


**Figure S40.** FC structure and generated post-ESPT geometry (pre-relaxation) for ωB97X trajectory. The CNH angle in the post-ESPT geometry was set to match the COH angle in the FC geometry (α). This post-ESPT geometry then underwent a constrained relaxation as discussed above.

**Table S17.** $\phi_S$ values (Figure 3 of the main text) for the FC and CI geometries of BEMT calculated with *ω*B97X and CAM-B3LYP.

| Functional | FC Geometry $\phi_S$ | CI Geometry $\phi_S$ |
|---|---|---|
| *ω*B97X | 1.4 | 287.3 |
| CAM-B3LYP | 1.8 | 286.1 |

### 2.5.1.2. MBBT:

None of the MBBT FC geometry relaxations in the first excited state resulted in a proton transferred geometry. Thus, post-ESPT geometries were generated from the four FC geometries: CAM-B3LYP MBBT 1, ωB97X MBBT 1, CAM-B3LYP MBBT 21 and ωB97X MBBT 21. This was achieved for each geometry by moving the transferring proton to the accepting nitrogen, fixing the resulting CNH angle to match the COH angle (β in Figure S41) in the FC geometry, and fixing N-H bond length to 0.95 Å. The resulting geometries then underwent constrained LR-TDDFT relaxations in $S_1$ in the gas phase using the same functionals as the earlier first excited state relaxations, with the $\phi_M$ angles (Figure 4 of the main text) being fixed to the values in the corresponding FC geometries. A linear interpolation was performed between the FC and resulting post-ESIPT geometries to obtain 20-step trajectories.

For the rotate-then-kink trajectories, the post-ESPT geometries underwent an initial dihedral rotation ($\phi_M$). The angle that changed most significantly between the FC and CI geometry for each system was chosen to rotate; the dihedral angles rotated for each of the above systems is specified in Figure S42 and Table S18. The $\phi_M$ values were adjusted iteratively, resulting in a 20-step trajectory rotating from the $\phi_M$ value in the FC geometries to the $\phi_M$ angle found in the CI geometries. $\theta_1$ and $\theta_2$ (Figure 4 of the main text) were fixed to the values in the FC geometries throughout this rotation. Next, $\theta_1$ and $\theta_2$ were iteratively adjusted over 20 steps from the values found in the FC geometries to the values found in the CI geometries, keeping $\phi_M$ values fixed to those found in CI geometries throughout.

For the kink-then-rotate trajectories, the same procedure was followed, except with the kinking (altering $\theta_1$ and $\theta_2$) occurring before the rotation (altering $\phi_M$).

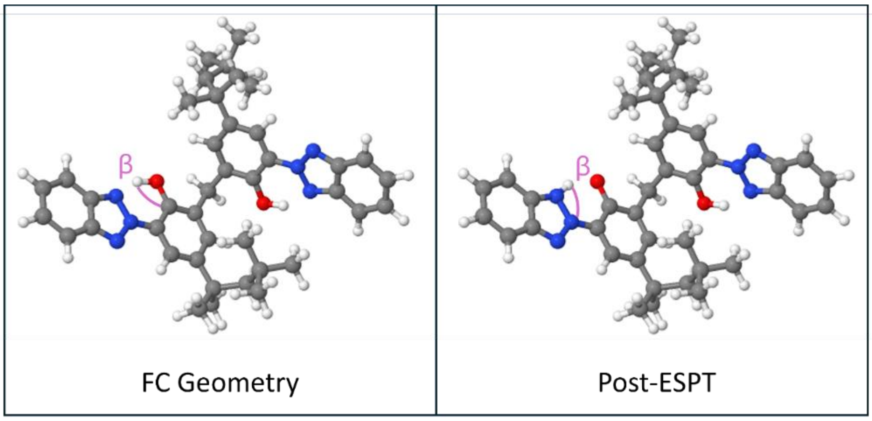


**Figure S41.** FC structure and generated post-ESPT geometry (pre-relaxation) for ωB97X trajectory. The NNH angle in the post-ESPT geometry was set to match the COH angle in the FC geometry (β). This post-ESPT geometry then underwent a constrained relaxation as discussed above.

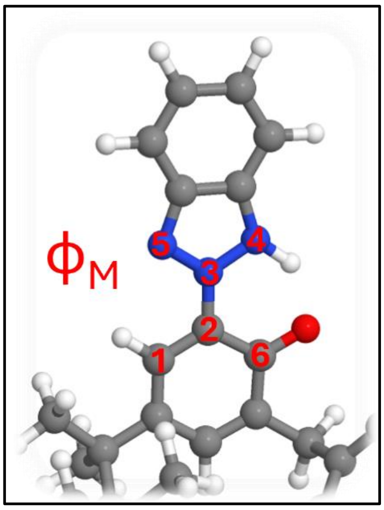


**Figure S42.** Labelled atoms involved in the dihedral angles that were adjusted to control the 'rotation' section of the MBBT trajectories.

**Table S18.** $\theta_1$, $\theta_2$ and $\phi_M$ (Figure 4 of the main text) values for the FC and CI geometries of BEMT calculated with $\omega$B97X and CAM-B3LYP. Lists encased in '[...]' correspond to the labelled atoms in Figure S42, specifying the atoms involved in $\phi_M$.

| System | Functional | Target Angle / [$\phi_M$ atoms (Fig. S42)] | FC Angle | CI Angle |
|---|---|---|---|---|
| MBBT 1 | $\omega$B97X | $\phi_M$ / [6,2,3,5] | 184.7 | 128.5 |
| MBBT 1 | $\omega$B97X | $\theta_1$ | 154.4 | 121.1 |
| MBBT 1 | $\omega$B97X | $\theta_2$ | 157.0 | 126.0 |
| MBBT 1 | CAM-B3LYP | $\phi_M$ / [6,2,3,5] | 179.1 | 129.3 |
| MBBT 1 | CAM-B3LYP | $\theta_1$ | 154.8 | 121.0 |
| MBBT 1 | CAM-B3LYP | $\theta_2$ | 156.6 | 125.9 |
| MBBT 21 | $\omega$B97X | $\phi_M$ / [1,2,3,4] | 177.1 | 115.5 |
| MBBT 21 | $\omega$B97X | $\theta_1$ | 154.2 | 113.2 |
| MBBT 21 | $\omega$B97X | $\theta_2$ | 157.0 | 107.3 |
| MBBT 21 | CAM-B3LYP | $\phi_M$ / [1,2,3,4] | 178.1 | 116.3 |
| MBBT 21 | CAM-B3LYP | $\theta_1$ | 154.5 | 112.6 |
| MBBT 21 | CAM-B3LYP | $\theta_2$ | 156.8 | 106.7 |

### 2.5.2. Potential energy surfaces

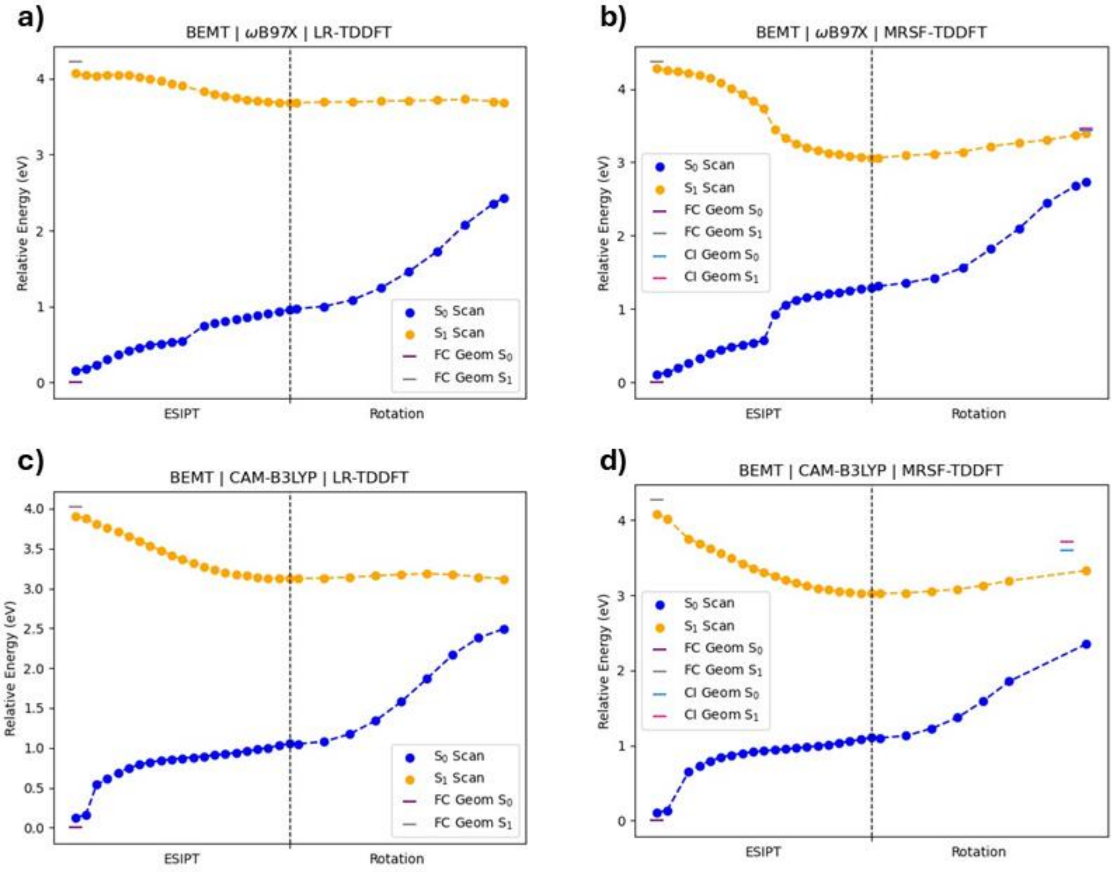


**Figure S43.** Evaluation of conformations obtained through constrained $S_1$ relaxations of interpolation geometries for BEMT at various levels of theory. Data points are represented by dots, connected with dashed lines; where data points are sparse, issues with calculation convergence precluded the procurement of valid energies. **a)** Conformations obtained through LR-TDDFT constrained relaxations with ωB97X, evaluated using DFT and LR-TDDFT with ωB97X. **b)** Conformations obtained through LR-TDDFT constrained relaxations with ωB97X, evaluated using MRSF-TDDFT with ωB97X. **c)** Conformations obtained through LR-TDDFT constrained relaxations with CAM-B3LYP, evaluated using DFT and LR-TDDFT with CAM-B3LYP. **d)** Conformations obtained through LR-TDDFT constrained relaxations with CAM-B3LYP, evaluated using MRSF-TDDFT with CAM-B3LYP.

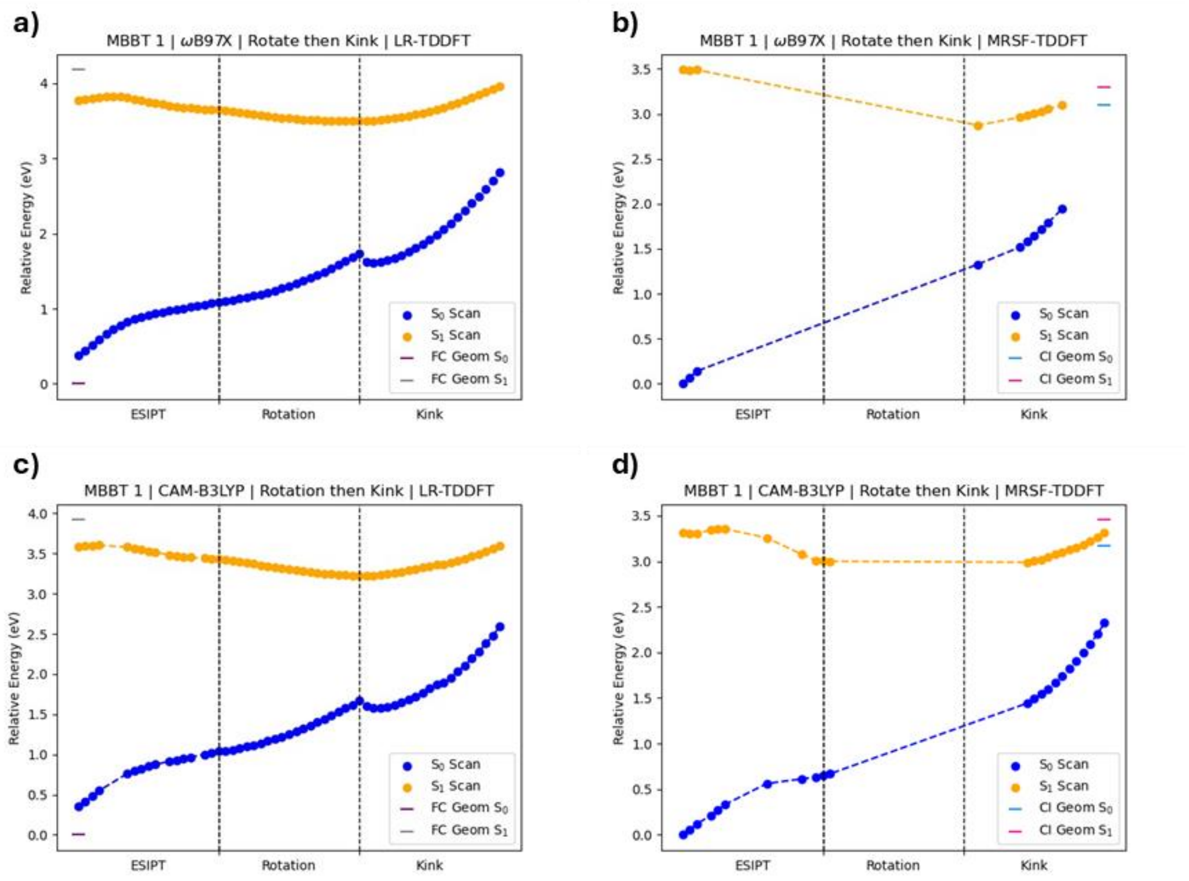


**Figure S44.** Evaluation of conformations obtained through constrained $S_1$ relaxations of interpolation geometries—following the ESIPT-rotation-kink pathway—for MBBT 1 at various levels of theory. Data points are represented by dots, connected with dashed lines; where data points are sparse, issues with calculation convergence precluded the procurement of valid energies. **a)** Conformations obtained through LR-TDDFT constrained relaxations with ωB97X, evaluated using DFT and LR-TDDFT with ωB97X. **b)** Conformations obtained through LR-TDDFT constrained relaxations with ωB97X, evaluated using MRSF-TDDFT with ωB97X. **c)** Conformations obtained through LR-TDDFT constrained relaxations with CAM-B3LYP, evaluated using DFT and LR-TDDFT with CAM-B3LYP. **d)** Conformations obtained through LR-TDDFT constrained relaxations with CAM-B3LYP, evaluated using MRSF-TDDFT with CAM-B3LYP.

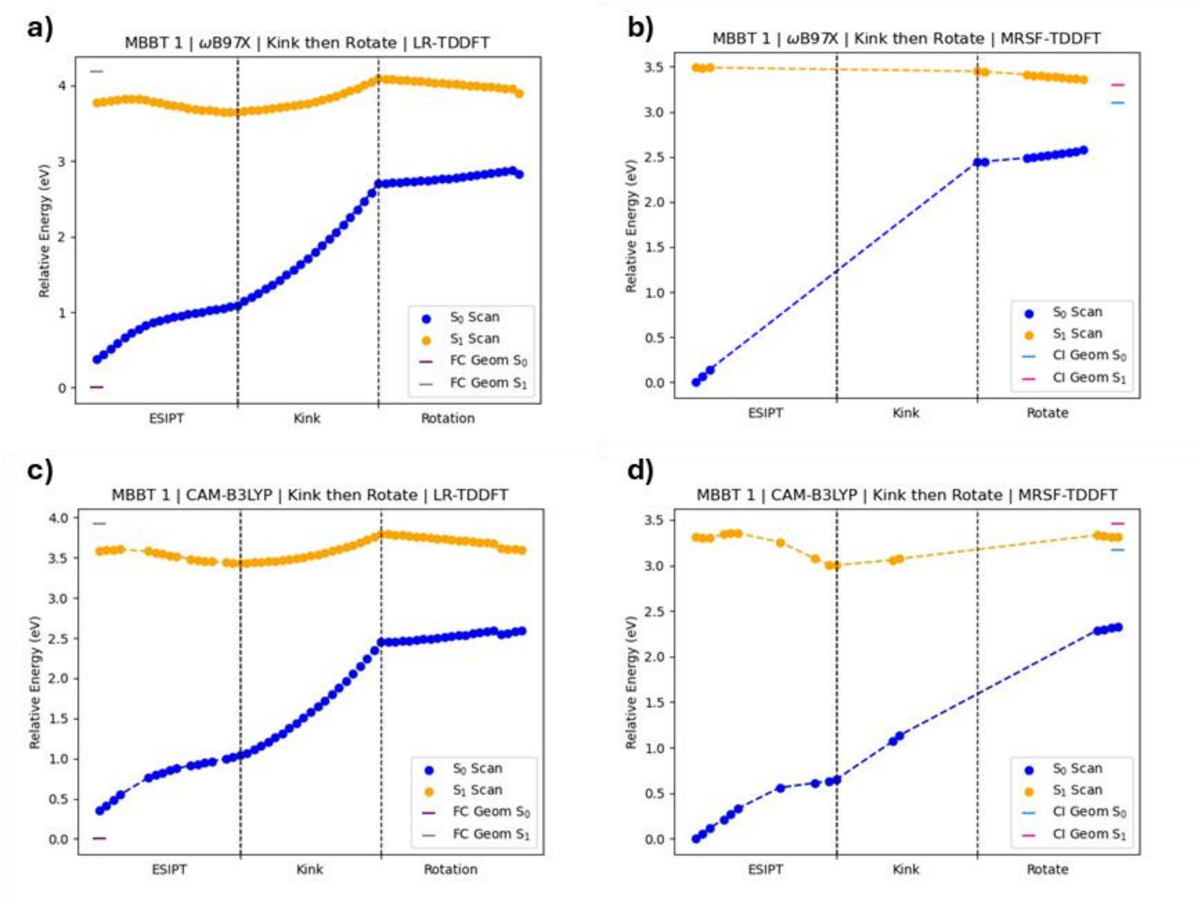


**Figure S45.** Evaluation of conformations obtained through constrained $S_1$ relaxations of interpolation geometries—following the ESIPT-kink-rotation pathway— for MBBT 1 at various levels of theory. Data points are represented by dots, connected with dashed lines; where data points are sparse, issues with calculation convergence precluded the procurement of valid energies. **a)** Conformations obtained through LR-TDDFT constrained relaxations with ωB97X, evaluated using DFT and LR-TDDFT with ωB97X. **b)** Conformations obtained through LR-TDDFT constrained relaxations with ωB97X, evaluated using MRSF-TDDFT with ωB97X. **c)** Conformations obtained through LR-TDDFT constrained relaxations with CAM-B3LYP, evaluated using DFT and LR-TDDFT with CAM-B3LYP. **d)** Conformations obtained through LR-TDDFT constrained relaxations with CAM-B3LYP, evaluated using MRSF-TDDFT with CAM-B3LYP.

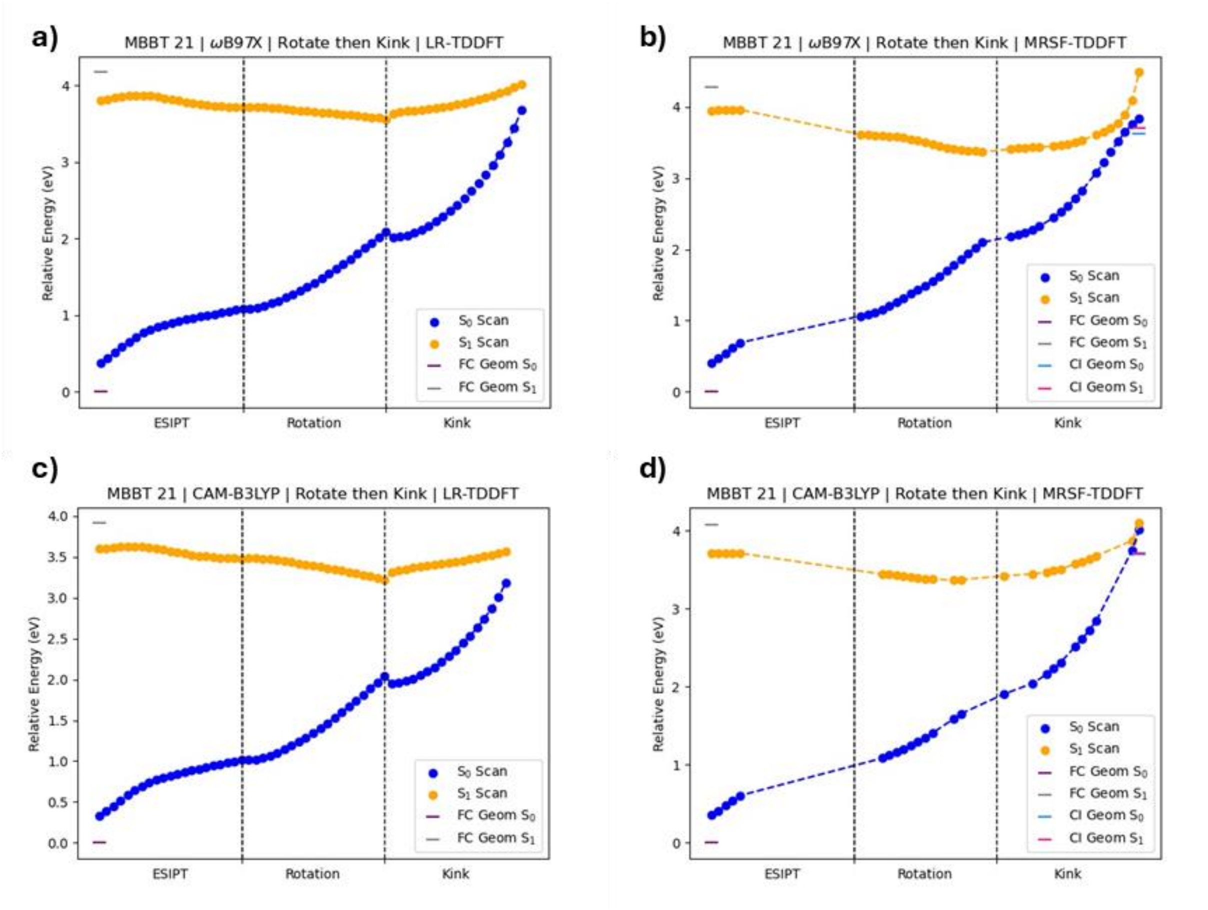


**Figure S46.** Evaluation of conformations obtained through constrained $S_1$ relaxations of interpolation geometries—following the ESIPT-rotation-kink pathway— for MBBT 21 at various levels of theory. Data points are represented by dots, connected with dashed lines; where data points are sparse, issues with calculation convergence precluded the procurement of valid energies. **a)** Conformations obtained through LR-TDDFT constrained relaxations with ωB97X, evaluated using DFT and LR-TDDFT with ωB97X. **b)** Conformations obtained through LR-TDDFT constrained relaxations with ωB97X, evaluated using MRSF-TDDFT with ωB97X. **c)** Conformations obtained through LR-TDDFT constrained relaxations with CAM-B3LYP, evaluated using DFT and LR-TDDFT with CAM-B3LYP. **d)** Conformations obtained through LR-TDDFT constrained relaxations with CAM-B3LYP, evaluated using MRSF-TDDFT with CAM-B3LYP.

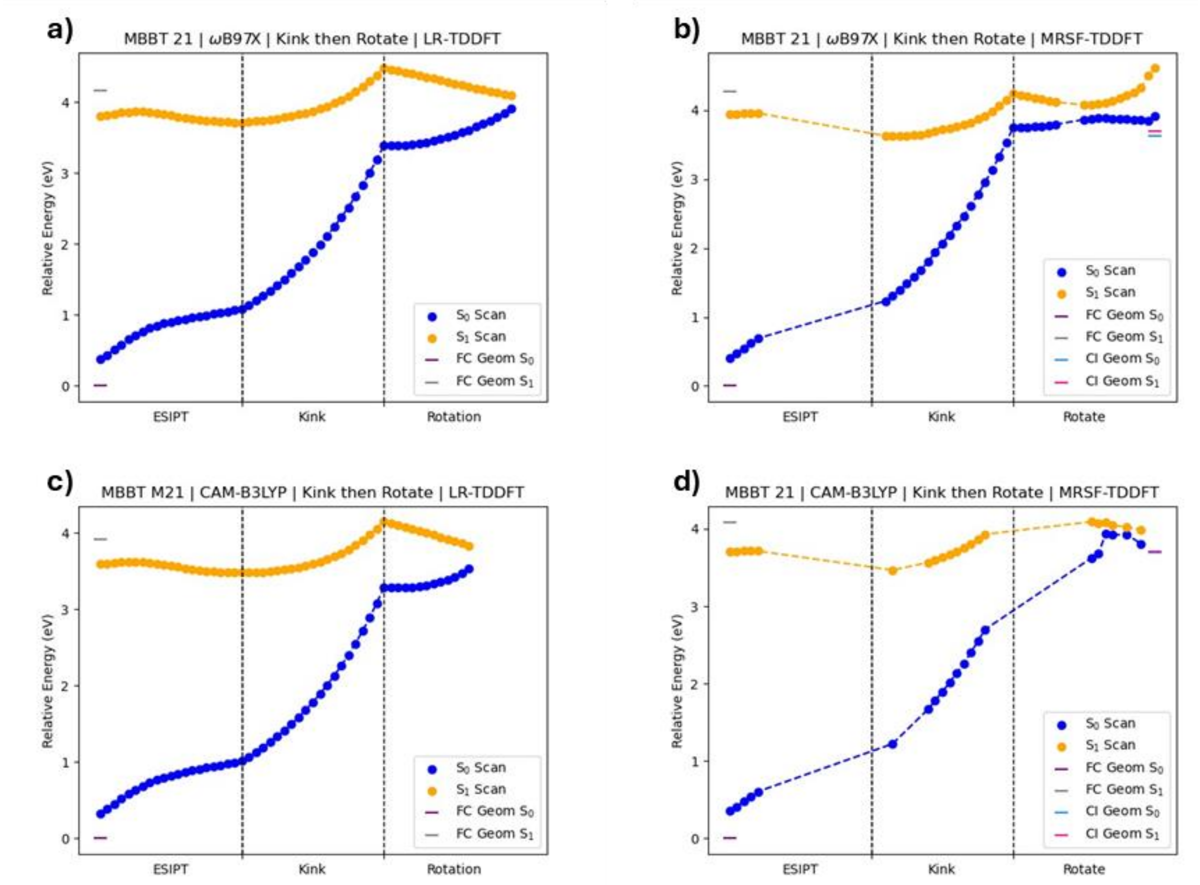


**Figure S47.** Evaluation of conformations obtained through constrained $S_1$ relaxations of interpolation geometries—following the ESIPT-kink-rotation pathway— for MBBT 21 at various levels of theory. Data points are represented by dots, connected with dashed lines; where data points are sparse, issues with calculation convergence precluded the procurement of valid energies. **a)** Conformations obtained through LR-TDDFT constrained relaxations with ωB97X, evaluated using DFT and LR-TDDFT with ωB97X. **b)** Conformations obtained through LR-TDDFT constrained relaxations with ωB97X, evaluated using MRSF-TDDFT with ωB97X. **c)** Conformations obtained through LR-TDDFT constrained relaxations with CAM-B3LYP, evaluated using DFT and LR-TDDFT with CAM-B3LYP. **d)** Conformations obtained through LR-TDDFT constrained relaxations with CAM-B3LYP, evaluated using MRSF-TDDFT with CAM-B3LYP.

**Table S19.** MRSF-TDDFT calculated energies of important geometries of BEMT.

| Functional | Geometry | $S_0$ Energy (eV) | $S_1$ Energy (eV) | Excitation Energy (eV)/ Wavelength (nm) |
|---|---|---|---|---|
| ωB97X | FC point | -54919.0196 | -54914.6542 | 4.3655/284.0117 |
| ωB97X | Post-ESPT | -54917.7222 | -54915.9557 | 1.7665/701.8635 |
| ωB97X | CI | -54915.5786 | -54915.5601 | 0.0185/66891.7529 |
| CAM-B3LYP | FC point | -54904.7385 | -54900.4721 | 4.2664/290.6061 |
| CAM-B3LYP | Post-ESPT | -54903.6324 | -54901.7159 | 1.9165/646.9303 |
| CAM-B3LYP | CI | -54901.1423 | -54901.0206 | 0.1216/10196.06850 |

**Table S20.** MRSF-TDDFT calculated energies of important geometries of MBBT 21. Where '*' follows a value, a converged calculation was not possible for the specified geometry; the quoted energy values correspond to the next geometry (along the translation coordinates) with a converged energy calculation.

| Functional | Geometry | $S_0$ Energy (eV) | $S_1$ Energy (eV) | Excitation Energy (eV)/ Wavelength (nm) |
|---|---|---|---|---|
| ωB97X | FC point | -56372.9161 | -56368.6416 | 4.2745/290.0554 |
| ωB97X | Post-ESPT | -56371.8507 * | -56369.3045 * | 2.5462 */486.9382 * |
| ωB97X | Rotated | -56370.7384 * | -56369.5096 * | 1.2288 */1008.9859 * |
| ωB97X | CI | -56369.2930 | -56369.2164 | 0.0765/16207.0841 |
| CAM-B3LYP | FC point | -56356.9326 | -56352.8526 | 4.0800/303.8829 |
| CAM-B3LYP | Post-ESPT | -56355.8474 * | -56353.4887 * | 2.3587 */525.6463 * |
| CAM-B3LYP | Rotated | -56355.0272 * | -56353.5110 * | 1.5162 */817.7298 * |
| CAM-B3LYP | CI | -56353.2380 | -56353.2252 | 0.01287/96335.8143 |

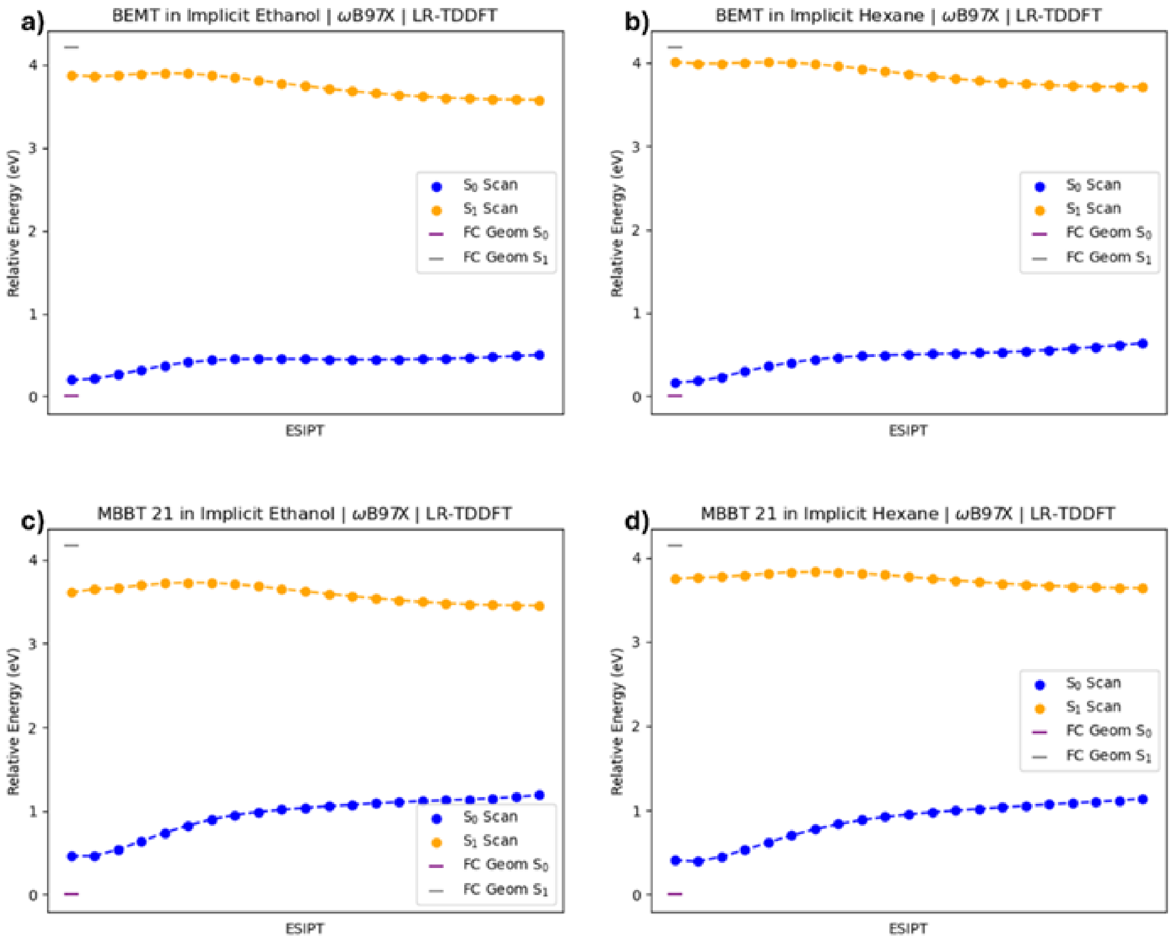


**Figure S48**. Ground and first excited state energies of conformations of BEMT and MBBT 21 obtained through implicitly solvated constrained LR-TDDFT (ωB97X) $S_1$ relaxations of interpolation geometries tracing ESIPT coordinates. Data points are represented by dots, connected with dashed lines. **a)** BEMT in implicit ethanol. **b)** BEMT in implicit hexane. **c)** MBBT 21 in implicit ethanol. **d)** MBBT 21 in implicit hexane.

## References

1. M. Bydder, A. Rahal, G. D. Fullerton and G. M. Bydder, *J. Magn. Reson. Imaging*, 2007, **25**, 290-300.
2. J. J. Snellenburg, S. Laptenok, R. Seger, K. M. Mullen and I. H. M. van Stokkum, *J. Stat. Softw.*, 2012, **49**, 1 - 22.
3. M. P. Grubb, A. J. Orr-Ewing and M. N. R. Ashfold, *Rev. Sci. Instrum.*, 2014, **85**.
4. H. Gonzalez, N. Tarras-Wahlberg, B. Strömdahl, A. Juzeniene, J. Moan, O. Larkö, A. Rosén and A. M. Wennberg, *BMC Dermatol.*, 2007, **7**, 1.
5. J. Kockler, M. Oelgemöller, S. Robertson and B. D. Glass, *J. Photochem. Photobiol., C: Photochem. Rev.*, 2012, **13**, 91-110.

6. J. Dalton, N. d. N. Rodrigues, D. Berndt and V. G. Stavros, *ACS Phys. Chem. Au.*, 2024, **4**, 750-760.
7. A. L. Whittock, A. M. Cowden, M. Wills and V. G. Stavros, *Phys. Chem. Chem. Phys.*, 2023, **25**, 7401-7406.
8. B. Herzog, D. Hüglin, E. Borsos, A. Stehlin and H. Luther, *Chimia*, 2004, **58**, 554-559.
9. J. T. Taylor, D. J. Tozer and B. F. E. Curchod, *J. Chem. Phys.*, 2023, **159**.
10. D. P. Chong, *Recent Advances in Density Functional Methods*, World Scientific, 1995.
11. J. Schirmer, *Phys. Chem. Chem. Phys.*, 2025, **27**, 4992-5005.
12. F. Neese, *WIREs Comput. Mol. Sci.*, 2012, **2**, 73-78.
13. F. Neese, *WIREs Comput. Molec. Sci.*, 2025, **15**, e70019.
14. V. Barone and M. Cossi, *J. Phys. Chem. A*, 1998, **102**, 1995-2001.
15. V. Mironov, K. Komarov, J. Li, I. Gerasimov, H. Nakata, M. Mazaherifar, K. Ishimura, W. Park, A. Lashkaripour, M. Oh, M. Huix-Rotllant, S. Lee and C. H. Choi, *J. Chem. Theory Comput.*, 2024, **20**, 9464-9477.
16. D. Weininger, *J. Chem. Inf. Comput. Sci.*, 1988, **28**, 31-36.
17. N. M. O'Boyle, M. Banck, C. A. James, C. Morley, T. Vandermeersch and G. R. Hutchison, *J. Cheminform.*, 2011, **3**, 33.
18. C. Adamo and V. Barone, *J. Chem. Phys.*, 1999, **110**, 6158-6170.
19. F. Weigend and R. Ahlrichs, *Phys. Chem. Chem. Phys.*, 2005, **7**, 3297-3305.
20. F. Weigend, *Phys. Chem. Chem. Phys.*, 2006, **8**, 1057-1065.
21. S. Grimme, S. Ehrlich and L. Goerigk, *J. Comput. Chem.*, 2011, **32**, 1456-1465.
22. S. Grimme, *J. Chem. Theory Comput.*, 2019, **15**, 2847-2862.
23. P. Pracht, F. Bohle and S. Grimme, *Phys. Chem. Chem. Phys.*, 2020, **22**, 7169-7192.
24. C. Bannwarth, S. Ehlert and S. Grimme, *J. Chem. Theory Comput.*, 2019, **15**, 1652-1671.
25. J. Eller and N. Hine, *Predicting Spectroscopic Properties of Solvated Nile Red with Automated Workflows for Machine Learned Interatomic Potentials*, 2025.
26. T. J. Zuehlsdorff, P. D. Haynes, M. C. Payne and N. D. M. Hine, *J. Chem. Phys.*, 2017, **146**.
27. M. A. P. Turner, M. D. Horbury, V. G. Stavros and N. D. M. Hine, *J. Phys. Chem. A*, 2019, **123**, 873-880.
28. T. Yanai, D. P. Tew and N. C. Handy, *Chem. Phys. Lett.*, 2004, **393**, 51-57.
29. N. Mardirossian and M. Head-Gordon, *Phys. Chem. Chem. Phys.*, 2014, **16**, 9904-9924.
30. A. D. Becke, *J. Chem. Phys.*, 1993, **98**, 1372-1377.
31. D. A. Case, H. M. Aktulga, K. Belfon, D. S. Cerutti, G. A. Cisneros, V. W. D. Cruzeiro, N. Forouzesh, T. J. Giese, A. W. Götz, H. Gohlke, S. Izadi, K. Kasavajhala, M. C. Kaymak, E. King, T. Kurtzman,

T.-S. Lee, P. Li, J. Liu, T. Luchko, R. Luo, M. Manathunga, M. R. Machado, H. M. Nguyen, K. A. O'Hearn, A. V. Onufriev, F. Pan, S. Pantano, R. Qi, A. Rahnamoun, A. Risheh, S. Schott-Verdugo, A. Shajan, J. Swails, J. Wang, H. Wei, X. Wu, Y. Wu, S. Zhang, S. Zhao, Q. Zhu, T. E. Cheatham, III, D. R. Roe, A. Roitberg, C. Simmerling, D. M. York, M. C. Nagan and K. M. Merz, Jr., *J. Chem. Inf. Model.*, 2023, **63**, 6183-6191.

32. B. A. M. C. Santos, A. C. P. da Silva, M. L. Bello, A. S. Gonçalves, T. A. Gouvêa, R. F. Rodrigues, L. M. Cabral and C. R. Rodrigues, *J. Photochem. Photobiol., A*, 2018, **356**, 219-229.
33. J. V. Teixeira Gomes, A. Cherem Peixoto da Silva, M. Lamim Bello, C. Rangel Rodrigues and B. Aloise Maneira Corrêa Santos, *J. Mol. Model.*, 2019, **25**, 362.
34. B. Herzog, J. Giesinger and V. Settels, *Photochem. Photobiol. Sci.*, 2020, **19**, 1636-1649.
35. Y. Li, Z. Tang, X. Zhou, J. Zhang, X. Song, K. Li, W. Liu and Z. Zhang, *J. Bioresour. Bioprod.*, 2024, **9**, 534-547.
